# Supplementary material for: Development of an affirming and customizable electronic survey of sexual and reproductive health experiences for transgender and gender nonbinary people
Source: PLoS One. 2020 May 4;15(5):e0232154. doi: 10.1371/journal.pone.0232154 (PMC7197812; doi:10.1371/journal.pone.0232154)
Supplement: S1 File — (DOCX) [file pone.0232154.s001.docx]

**S1 File. Form of interest posted to recruit Community Advisory Team members**

We are seeking 3-5 individuals for a Community Advisory Team to support a research study being conducted in the United States. The study will focus on barriers to contraception and abortion care for Transgender and Gender Expansive (TGE) people assigned female at birth. Results from this study will inform the development of evidence-based recommendations for increasing access to and improving quality of contraceptive and abortion care for TGE individuals. More broadly, we aim to support high-quality, gender affirming and trans-inclusive sexual and reproductive health care across the country.

Members of the Community Advisory Team will contribute approximately 25 hours total over several months on a variety of study support activities. Specific tasks will include assistance in recruitment of individuals who identify as TGE; feedback on research tools (for example, survey questions) with a focus on inclusive language; and contributions to interpretation of research findings. We hope to create a Community Advisory Team that includes TGE individuals from around the country who represent a range of gender identities, racial identities, and ages.

All Community Advisory Team members will be paid for their time and insight. If you are interested in participating, please fill out the form below. Thank you!

- 1. What is your name?
  2. How would you describe your gender identity?
  3. How would you describe your racial identity?
  4. What are your pronouns?
  5. How old are you?
  6. What interests you about being involved in this project?
  7. Where do you currently live?
  8. What is the best way for us to contact you about being involved in this project? Please provide either email or phone number.
