# Supplementary material for: Development of an affirming and customizable electronic survey of sexual and reproductive health experiences for transgender and gender nonbinary people
Source: PLoS One. 2020 May 4;15(5):e0232154. doi: 10.1371/journal.pone.0232154 (PMC7197812; doi:10.1371/journal.pone.0232154)
Supplement: S2 File — Exported to Word from Qualtrics. (PDF) [file pone.0232154.s002.pdf]

Pop B - Sexual and Reproductive Health Survey

Survey Flow

EmbeddedData

pidValue will be set from Panel or URL.
statusValue will be set from Panel or URL.
testuserValue will be set from Panel or URL.
fnameValue will be set from Panel or URL.
email1Value will be set from Panel or URL.
email1_verifiedValue will be set from Panel or URL.
email_allowedValue will be set from Panel or URL.
telephone1Value will be set from Panel or URL.
telephone1_verifiedValue will be set from Panel or URL.
sms_allowedValue will be set from Panel or URL.
dobValue will be set from Panel or URL.
zipValue will be set from Panel or URL.
asexualValue will be set from Panel or URL.
bisexualValue will be set from Panel or URL.
gayValue will be set from Panel or URL.
lesbianValue will be set from Panel or URL.
queerValue will be set from Panel or URL.
questioningValue will be set from Panel or URL.
straightValue will be set from Panel or URL.
samegenderlovingValue will be set from Panel or URL.
pansexualValue will be set from Panel or URL.
so_otherValue will be set from Panel or URL.
genderqueerValue will be set from Panel or URL.
manValue will be set from Panel or URL.
transmanValue will be set from Panel or URL.
womanValue will be set from Panel or URL.
transwomanValue will be set from Panel or URL.
gi_otherValue will be set from Panel or URL.
intersexValue will be set from Panel or URL.
maleValue will be set from Panel or URL.
amerindianValue will be set from Panel or URL.
asianValue will be set from Panel or URL.
blackValue will be set from Panel or URL.
hawaiianValue will be set from Panel or URL.
whiteValue will be set from Panel or URL.
race_otherValue will be set from Panel or URL.
menaValue will be set from Panel or URL.
immigrantValue will be set from Panel or URL.
hispanicValue will be set from Panel or URL.
languageValue will be set from Panel or URL.
educationValue will be set from Panel or URL.
weight_unitValue will be set from Panel or URL.
height1_unitValue will be set from Panel or URL.

height2_unitValue will be set from Panel or URL.
refluxValue will be set from Panel or URL.
cadValue will be set from Panel or URL.
liverdzValue will be set from Panel or URL.
anemiaValue will be set from Panel or URL.
depressionValue will be set from Panel or URL.
sleValue will be set from Panel or URL.
anginaValue will be set from Panel or URL.
dmValue will be set from Panel or URL.
anxietyValue will be set from Panel or URL.
dm_borderlineValue will be set from Panel or URL.
migraineValue will be set from Panel or URL.
asthmaValue will be set from Panel or URL.
edValue will be set from Panel or URL.
osaValue will be set from Panel or URL.
afibValue will be set from Panel or URL.
glaucomaValue will be set from Panel or URL.
pvdValue will be set from Panel or URL.
bphValue will be set from Panel or URL.
miValue will be set from Panel or URL.
psoriasisValue will be set from Panel or URL.
bipolarValue will be set from Panel or URL.
murmurValue will be set from Panel or URL.
peValue will be set from Panel or URL.
cancerValue will be set from Panel or URL.
hldValue will be set from Panel or URL.
epilepsyValue will be set from Panel or URL.
cataractValue will be set from Panel or URL.
hivValue will be set from Panel or URL.
cvaValue will be set from Panel or URL.
ckdValue will be set from Panel or URL.
htnValue will be set from Panel or URL.
thyroidValue will be set from Panel or URL.
copdValue will be set from Panel or URL.
ibdValue will be set from Panel or URL.
ulcerValue will be set from Panel or URL.
coagValue will be set from Panel or URL.
ibsValue will be set from Panel or URL.
chfValue will be set from Panel or URL.
stoneValue will be set from Panel or URL.
no_pmhValue will be set from Panel or URL.
uiValue will be set from Panel or URL.
cppValue will be set from Panel or URL.
lbpValue will be set from Panel or URL.

arthritisValue will be set from Panel or URL.
no_sxValue will be set from Panel or URL.
stentValue will be set from Panel or URL.
ccyValue will be set from Panel or URL.
cabgValue will be set from Panel or URL.
appyValue will be set from Panel or URL.
valveValue will be set from Panel or URL.
ppmValue will be set from Panel or URL.
icdValue will be set from Panel or URL.
bmtValue will be set from Panel or URL.
sotValue will be set from Panel or URL.
no_pshValue will be set from Panel or URL.
hearttxValue will be set from Panel or URL.
lungtxValue will be set from Panel or URL.
oltValue will be set from Panel or URL.
panctxValue will be set from Panel or URL.
kidneytxValue will be set from Panel or URL.
smboweltxValue will be set from Panel or URL.
sot_otherValue will be set from Panel or URL.
gasValue will be set from Panel or URL.
browValue will be set from Panel or URL.
chinValue will be set from Panel or URL.
foreValue will be set from Panel or URL.
jawValue will be set from Panel or URL.
lipliftValue will be set from Panel or URL.
rhinoValue will be set from Panel or URL.
scalpValue will be set from Panel or URL.
trachValue will be set from Panel or URL.
vocalValue will be set from Panel or URL.
breast_augValue will be set from Panel or URL.
breast_redValue will be set from Panel or URL.
top_infValue will be set from Panel or URL.
top_keyValue will be set from Panel or URL.
ooph_gasValue will be set from Panel or URL.
salping_gasValue will be set from Panel or URL.
sch_gasValue will be set from Panel or URL.
total_hyst_gasValue will be set from Panel or URL.
vaginectValue will be set from Panel or URL.
metaValue will be set from Panel or URL.
phalloValue will be set from Panel or URL.
phallo_impValue will be set from Panel or URL.
scrotoValue will be set from Panel or URL.
testi_impValue will be set from Panel or URL.
orchiValue will be set from Panel or URL.

labioplastValue will be set from Panel or URL.
vaginoplast_cValue will be set from Panel or URL.
vaginoplast_pValue will be set from Panel or URL.
fatgraftValue will be set from Panel or URL.
fillerValue will be set from Panel or URL.
gas_otherValue will be set from Panel or URL.
email2Value will be set from Panel or URL.
menopauseValue will be set from Panel or URL.
fibroidValue will be set from Panel or URL.
aubValue will be set from Panel or URL.
imbValue will be set from Panel or URL.
csectionValue will be set from Panel or URL.
schValue will be set from Panel or URL.
total_hystValue will be set from Panel or URL.
oophValue will be set from Panel or URL.
stateValue will be set from Panel or URL.
weightValue will be set from Panel or URL.
height1Value will be set from Panel or URL.
height2Value will be set from Panel or URL.
telephone2Value will be set from Panel or URL.
breastcaValue will be set from Panel or URL.
coloncaValue will be set from Panel or URL.
rccValue will be set from Panel or URL.
lungcaValue will be set from Panel or URL.
leukValue will be set from Panel or URL.
ovariancaValue will be set from Panel or URL.
pancreascaValue will be set from Panel or URL.
prostatecaValue will be set from Panel or URL.
melanomaValue will be set from Panel or URL.
skincaValue will be set from Panel or URL.
othercaValue will be set from Panel or URL.

Standard: Age Screening Block (2 Questions)

Branch: New Branch

If

If Thank you for your interest in this study about your sexual and reproductive health needs and exp... Text Response Is Less Than 18

Or Thank you for your interest in this study about your sexual and reproductive health needs and exp... Text Response Is Greater Than 45

EndSurvey: Advanced

Standard: Gender Identity Screening Block (3 Questions)

Branch: New Branch

If

If If you had to choose from the list below, although we acknowledge that these categories may not b... Cisgender Woman (a person that identifies as a woman and was assigned female sex at birth) Is Selected

EndSurvey: Advanced

Branch: New Branch

If

If What sex were you assigned at birth, for example on your original birth certificate? Male Is Selected

Or What sex were you assigned at birth, for example on your original birth certificate? Prefer not to say Is Selected

EndSurvey: Advanced

Block: Intersex Screening Block (3 Questions)
Standard: Country Screening Block (1 Question)

Branch: New Branch

If

If Which country do you currently live in? Another country (please specify): Is Selected

Or Which country do you currently live in? Prefer not to say Is Selected

EndSurvey: Advanced

Standard: PRIDE Study Participant Screening Block (1 Question)

Branch: New Branch

If

If This study is a collaboration between Ibis Reproductive Health, The PRIDE Study of Stanford Unive... Yes Is Selected

EndSurvey: Advanced

Standard: Consent Screening Block (1 Question)

Branch: New Branch

If

If Details about study participation: informed consent materials This is a research study about th... No, I do not give my consent to participate in this study Is Selected

EndSurvey: Advanced

Standard: Introduction (1 Question)
Standard: Introductory questions/preferred language (11 Questions)
Standard: Gender affirmation and sexual behavior (102 Questions)

Standard: Birth control (26 Questions)
Standard: Current pregnancy (4 Questions)
Standard: Fertility desires (10 Questions)

Branch: New Branch
If
If How many times have you been pregnant? Text Response Is Greater Than or Equal to 1

Standard: Pregnancy history (5 Questions)

Branch: New Branch
If
If How many times have you been ${age8/ChoiceTextEntryValue/2}? Text Response Is Greater Than or Equal to 1

Block: Pregnancy history w piped in language (5 Questions)

Standard: Pregnancy and health care (23 Questions)
Standard: Sexual and reproductive health care (95 Questions)
Standard: Quality of health care (8 Questions)
Standard: Sociodemographic characteristics (22 Questions)
Standard: Email Block (1 Question)

Branch: New Branch
If
If Thank you so much for taking the time to complete this survey! If you know of people that may be... Yes, I want to share my email with the study team Is Selected

EndSurvey: Advanced

EndSurvey: Advanced

Page Break

Start of Block: Age Screening Block

Q328
Depending on the browser you are using, you may need to scroll up to the top of each survey screen to see all the questions.
Some browsers will auto-scroll to the top of each survey screen, while others do not. We recommend using Chrome.
We apologize for the inconvenience.

Please advance to the next screen to start this survey.

Page Break

age Thank you for your interest in this study about your sexual and reproductive health needs and experiences. This study has been designed by a diverse team. Members of our team identify as transgender, gender-expansive, and cisgender, and express a range of sexual orientations. The team includes experts in sexual and reproductive health. This survey is designed for transgender and gender expansive (TGE) people assigned female or another sex at birth.
We anticipate this study will take 15-45 minutes to complete.

How old are you (in years)?

____

End of Block: Age Screening Block

Start of Block: Gender Identity Screening Block

gid_string
A person's gender identity is an important, inner concept of self which can include girl/woman, boy/man, a blend of both, neither, and many more. Gender identity is how each person thinks of themselves and what they call themselves. One's gender identity can be consistent with, or different than, their sex assigned at birth. The words a person uses to talk about their gender identity can change over time.

What is your gender identity? In other words, how do you describe your gender?

____

____

____

____

____

gid_mc If you had to choose from the list below, although we acknowledge that these categories may not be ideal, what best describes your current gender identity at this time? **Select all that apply.**

- ☐ Agender (1)
- ☐ Cisgender Man (a person that identifies as a man and was assigned male sex at birth) (2)
- ☐ Cisgender Woman (a person that identifies as a woman and was assigned female sex at birth) (3)
- ☐ Genderqueer (4)
- ☐ Man (5)
- ☐ Non-binary (6)
- ☐ Transgender Man (7)
- ☐ Transgender Woman (8)
- ☐ Two-Spirit (feel free to include your tribe's specific language for your identity, if you would like) (9) ____
- ☐ Woman (10)
- ☐ Additional gender category, please specify: (11) ____
- ☐ ☐ Prefer not to say (12)

saab What sex were you assigned at birth, for example on your original birth certificate?

- ◯ Female (1)
- ◯ Male (2)
- ◯ Not listed (please specify) (3) ____
- ◯ Prefer not to say (4)

End of Block: Gender Identity Screening Block

Start of Block: Intersex Screening Block

intersex Do you identify as intersex?

- ◯ No (0)
- ◯ Yes (1)
- ◯ Prefer not to say (2)

---

intersex_hcp Has a health care provider ever told you that you are intersex, or that you have what is sometimes referred to as either a "difference of sex development" or a "disorder of sex development" (DSD)?

- ◯ No (0)
- ◯ Yes (1)
- ◯ I don't know (2)
- ◯ Prefer not to say (3)

Display This Question:

If intersex_hcp = Yes

intersex_string Can you tell us more about what your health care provider told you about your specific diagnosis? (The diagnosis of being intersex or having a DSD)

____

____

____

____

____

End of Block: Intersex Screening Block

Start of Block: Country Screening Block

country Which country do you currently live in?

- ◯ The United States (or a United States territory) (1)
- ◯ Another country (please specify): (2)
- ◯ Prefer not to say (3)

End of Block: Country Screening Block

Start of Block: PRIDE Study Participant Screening Block

tps This study is a collaboration between Ibis Reproductive Health, The PRIDE Study of Stanford University, and many community members and researchers. To reach as many people as possible, we are releasing this study in different ways. One way is the survey you have in front of you and another way is through The PRIDE Study. If you are a participant of The PRIDE Study (pridestudy.org) we want you to continue the survey there.
Are you a participant in The PRIDE Study? The PRIDE Study is a national online general health study of sexual and gender minority people which includes people who identify as LGBTQ+, more info at pridestudy.org.

○ No (0)

○ Yes (1)

End of Block: PRIDE Study Participant Screening Block

Start of Block: Consent Screening Block

consent
Details about study participation: informed consent materials
This is a research study about the sexual and reproductive health needs and experiences of transgender and gender expansive (TGE) people assigned female or another sex at birth. This study is led by Dr. Juno Obedin-Maliver (Co-Director of The PRIDE Study) at Stanford University. Research studies include only people who choose to take part. Please take your time to make your decision about participating, and discuss your decision with your family or friends if you wish. If you have any questions, you may contact the researchers.

You are being asked to take part in this study because you are or have indicated that you identify as transgender or gender expansive and were assigned female or another sex at birth. The purposes of this study are to improve our understanding of people's needs for and experiences with family planning care (particularly pregnancy prevention and abortion services), so that we can improve the quality, relevance, and gender- and sexuality-affirming nature of those services for all people. This study is funded by the Society for Family Planning, a 501c3 (not-for-profit) organization based in the United States.

What will happen if I take part in this research study?
If you agree to participate in this study, you will be asked to complete a 15-45 minute survey about your lived experiences with gender and sexual identity, sexual practices related to pregnancy, preferred language for various sexual and reproductive health topics, desire for and experiences of seeking pregnancy prevention and abortion care, and recommendations for improving the quality of sexual and reproductive health care for transgender and gender expansive (TGE) people. Some survey questions may ask about sensitive information including sexual practices and experiences of discrimination.

RISKS AND BENEFITS:

Are there benefits to taking part in the study?
You will not receive any direct health benefits from participating in this study. However, the information that you provide may help health professionals learn more about the influence of sexual orientation and gender identity on sexual and reproductive health, and health care. The study team will create documents from the things we learn from this study, which we may make available to you as a participant. We hope that society will benefit from your participation. By participating, you will help us have a better understanding of health issues that affect transgender and gender expansive people.

What side effects or risks can I expect from being in the study?
Some of the survey questions may make you uncomfortable or upset, but you are free to not answer any questions (or select "Prefer not to say") if you do not wish to answer. For more information about risks and side effects, contact one of the researchers.

TIME INVOLVEMENT:
If you agree to participate in this study, you will be asked to complete a 15-45 minute survey. You will be in the study only as long as it takes you to complete the survey (less than one day). We will not contact you again, unless you give express permission to do so. Even then, you will only be contacted if another research study is developed for which you are eligible, and you will be able to ignore and/or decline participation at your discretion.

PAYMENTS/REIMBURSEMENTS:
You will not be paid for taking part in this study. We will, however, hold a raffle for multiple $50 gift cards as a thank you to interested individuals. If you provide your contact information, you will be entered into a raffle to be conducted on or before September 15, 2019 (after the survey is closed) for a gift card valued at $50. The drawing will be conducted by The PRIDE Study of Stanford University in Palo Alto, California. Participation in the study is not required in order to participate in the raffle. You can enter the raffle if you do not start or complete the study task. The chance of winning a prize will vary depending on the number of people who express interest, and we estimate that it will never be worse than 1 in 100. The winner will be notified immediately by email and provided with information on how to receive the prize.

PARTICIPANT'S RIGHTS: If you have read this form and have decided to participate in this project, please understand your participation is voluntary and you have the right to withdraw your consent or discontinue participation at any time without penalty or loss of benefits to which you are otherwise entitled. You have the right to refuse to answer particular questions. The results of this research study may be presented at scientific or professional meetings or published in scientific journals. However, your identity will not be disclosed. You have the right to refuse to answer particular questions.

Authorization To Use Your Health Information For Research Purposes

Because information about you and your health is personal and private, it generally cannot be

used in this research study without your written authorization. If you sign this form, it will provide that authorization. The form is intended to inform you about how your health information will be used or disclosed in the study. Your information will only be used in accordance with this authorization form and the informed consent form and as required or allowed by law. Please read it carefully before signing it.

What is the purpose of this research study and how will my health information be utilized in the study?

The purposes of this study are to improve our understanding of people's needs for and experiences with family planning care (particularly pregnancy prevention and abortion services), so that we can improve the quality, relevance, and gender- and sexuality-affirming nature of those services for all people.

Do I have to sign this authorization form?

You do not have to sign this authorization form. But if you do not, you will not be able to participate in this research study. Signing the form is not a condition for receiving any medical care outside the study.

If I sign, can I revoke it or withdraw from the research later?

If you decide to participate, you are free to withdraw your authorization regarding the use and disclosure of your health information (and to discontinue any other participation in the study) at any time. Unfortunately, because this survey is anonymous and saved screen-by-screen after each entry, we almost certainly cannot delete your survey responses after you have submitted them unless you know the date, precise start and stop time of the survey, and the internet protocol (IP) address you were using at the time of survey data entry. Without this information, it may be impossible for us to differentiate your data from those of other participants' and therefore very difficult to delete. However, if you would like your data removed we will work with you to try to delete your data given those stipulations above. If you wish to revoke your authorization for the research use or disclosure of your health information in this study, you must write to: Juno Obedin-Maliver, MD, MPH, MAS by mail at The PRIDE Study at Stanford University, 1701 Page Mill Road, Palo Alto, CA, 94304, or by telephone at 1-855-421-9991 (toll-free), or by e-mail at junoom@stanford.edu.

What Personal Information Will Be Obtained, Used or Disclosed?

Your health information related to this study, may be used or disclosed in connection with this research study. **We will not access your medical records**. All health information is provided directly by you to us for research purposes. All information you provide to us will be obtained only through information you enter in the electronic survey you complete, with the exception of your IP address, which is automatically stored by the Qualtrics platform. We will use your IP address only to check for duplicate survey entries. After duplicate survey entries have been assessed, we will delete all IP address data. You will only be asked for your contact information for two reasons, 1) if you would like us to be in touch with you about future research and / or the results of this research, and 2) to enter your name into a drawing for compensation for your time and effort. The contact information is not linked to your survey responses and therefor the

surveys themselves are anonymous. This anonymity provides you an additional layer of protection and helps prevent the information you provide on your survey answers from being linked to you and your name. However, because this survey is anonymous and saved screen-by-screen after each entry, we cannot delete your survey responses after you have submitted them unless you know the date, precise start and stop time of the survey, and the internet protocol (IP) address you were using at the time of survey data entry. Without this information, it may be impossible for us to differentiate your data from those of other participants' and therefore very difficult to delete.

Who May Use or Disclose the Information?
The following parties are authorized to use and/or disclose your health information in connection with this research study: The Protocol Directors Juno Obedin-Maliver, MD, MPH, MAS (The PRIDE Study - Stanford), The Stanford University Administrative Panel on Human Subjects in Medical Research and any other unit of Stanford University as necessary, Research Staff.

Who May Receive or Use the Information?
The parties listed in the preceding paragraph may disclose your health information to the following persons and organizations for their use in connection with this research study: The Office for Human Research Protections in the U.S.; Department of Health and Human Services; The Society for Family Planning. Your information may be re-disclosed by the recipients described above, if they are not required by law to protect the privacy of the information.

When will my authorization expire?
Your authorization for the use and/or disclosure of your health information will end on December 31, 2038 or when the research project ends, whichever is earlier.

WITHDRAWAL FROM STUDY
The Protocol Directors may also withdraw you from the study without your consent for one or more of the following reasons: Failure to follow the instructions of the Protocol Director(s) and study staff; The Protocol Director decides that continuing your participation could be harmful to you; the study is cancelled; Other administrative reasons; or Unanticipated circumstances.

Contact Information:
If you have any questions, concerns or complaints about this research study, its procedures, risks and benefits, or alternative courses of treatment, you should ask the Protocol Directors: Juno Obedin-Maliver, MD, MPH, MAS by mail at The PRIDE Study at Stanford University 1701 Page Mill Road, Palo Alto, CA, 94304, by telephone at 1-855-421-9991 (toll-free), or by e-mail at junoom@stanford.edu. You should also contact them at any time if you feel you have been harmed by being a part of this study.

Independent Contact: If you are not satisfied with how this study is being conducted, or if you have any concerns, complaints, or general questions about the research or your rights as a participant, please contact the Stanford Institutional Review Board (IRB) to speak to someone

independent of the research team at (650)-723-5244 or toll free at 1-866-680-2906. You can also write to the Stanford IRB, Stanford University, 3000 El Camino Real, Five Palo Alto Square, 4th Floor, Palo Alto, CA 94306.

CONSENT
Please print a copy of this page for your records. Should you be unable to print a copy and wish to obtain a printed copy of the consent for your records, please contact us by telephone at 1-855-421-9991 (toll-free), or by e-mail at contact@pridestudy.org.

PARTICIPATION IN RESEARCH IS VOLUNTARY. <u>You have the right to decline to be in this study, or to withdraw from it at any point without penalty or loss of benefits to which you are otherwise entitled.</u>

<u>**If you wish to participate in this study, please check the box that says “Yes, I have read, understand, and agree to the consent above.”**</u>

○ Yes, I have read, understood, and agree to the consent above (1)

○ No, I do not give my consent to participate in this study (2)

End of Block: Consent Screening Block

Start of Block: Introduction

intro Thank you for taking the time to participate in this study. We will be asking questions about pregnancy prevention with an emphasis on birth control (contraception) and abortion. We will ask you questions about your experience with health care providers which might include medical doctors, midwives, nurse practitioners, physician assistants, medical assistants, pharmacists, and other health care professionals involved in your sexual and reproductive health care.

Please feel free to skip any question that makes you uncomfortable. For some questions, you are not able to skip the question - but you CAN select the answer choice "Prefer not to say" to move on to the next question. We are grateful for any information you are willing to share.

We want to acknowledge up front that some of the wording in this survey is more medical/clinical than the words that you may normally use. This is to ensure that the results are as specific and helpful as possible for improving the care that healthcare providers offer to all patients.

This is NOT a test; there are no “right” or “wrong” answers. Please answer as honestly as you can. Your responses will be kept strictly confidential.

End of Block: Introduction

Start of Block: Introductory questions/preferred language

pronouns What pronouns do you use? **Select all that apply.**

- ☐ He/him (1)
- ☐ She/her (2)
- ☐ They/them (3)
- ☐ Ze/hir (4)
- ☐ No pronouns. I only use my name. (5)
- ☐ Pronouns not listed above (please describe): (6) ____
- ☐ ☐ Unsure (7)

---

wordintro Next is a list of medical words for various body parts and experiences related to sex and fertility (the ability to get pregnant). We may ask you about these body parts in reference to your own body or to another person's body, such as a sexual partner. For each word, please let us know if you use the word listed. If you use another word, please write it in.
To improve your overall survey experience, we will use your preferred words for each of the following items whenever possible in this survey, beginning AFTER this section. We will not be able to display your own words until AFTER this section is completed.

---

word_uterus A **uterus** is an organ that is about the size of a fist, and it sits in the pelvic area. Monthly bleeding comes from the uterus, and it is where a pregnancy grows.

After reading this definition, please tell us if you use this word, or what word you use instead.

○ Yes, I use the word "uterus". (1)

○ No, I use a different word. The word I use instead of "uterus" is: (0)
____

○ Prefer not to say (2)

---

word_vagina A **vagina** is a frontal genital opening, used by some people for sexual activity, and also by some people for releasing menstrual blood or giving birth.

After reading this definition, please tell us if you use this word, or what word you use instead.

○ Yes, I use the word "vagina". (1)

○ No, I use a different word. The word I use instead of "vagina" is: (0)
____

○ Prefer not to say (2)

---

word_period A **period** is the bleeding from the frontal genital opening that often occurs about every 3 to 5 weeks when the uterus sheds its lining.

After reading this definition, please tell us if you use this word, or what word you use instead.

○ Yes, I use the word "period". (1)

○ No, I use a different word. The word I use instead of "period" is: (0)
____

○ Prefer not to say (2)

---

word_breasts The **breasts** are fleshy organs on the chest of many teenagers and adults.

After reading this definition, please tell us if you use this word, or what word you use instead.

- ○ Yes, I use the word "breasts". (1)
- ○ No, I use a different word. The word I use instead of "breasts" is: (0) ________________
- ○ Prefer not to say (2)

Page Break

word_penis The **penis** is a phallus that is a part of the body near the groin, used by some people for sexual activity, and also by some people for releasing urine and sperm from the body.

After reading this definition, please tell us if you use this word, or what word you use instead.

- ◯ Yes, I use the word "penis". (1)
- ◯ No, I use a different word. The word I use instead of "penis" is: (0) ____
- ◯ Prefer not to say (2)

---

word_sperm **Sperm** are reproductive cells that are carried in a whitish fluid (called semen). For some people, sperm comes out of the penis.

After reading this definition, please tell us if you use this word, or what word you use instead.

- ◯ Yes, I use the word "sperm". (1)
- ◯ No, I use a different word. The word I use instead of "sperm" is: (0) ____
- ◯ Prefer not to say (2)

---

word_preg To be **pregnant** is to have cells growing/dividing in the uterus that could turn into a baby.

After reading this definition, please tell us if you use this word, or what word you use instead.

- ○ Yes, I use the word "pregnant". (1)
- ○ No, I use a different word. The word I use instead of "pregnant" is: (0) ____
- ○ Prefer not to say (2)

X→

word_bc **Birth control** can be used for more than one reason. Some people use birth control to avoid getting pregnant. It can include using a condom, not having any sex (abstinence), taking a pill every day to prevent pregnancy, having been sterilized, having an implant put in your arm, or many other medications, devices, and/or practices. Some people use birth control for non-pregnancy related reasons, like gender-affirmation, clearing up their skin, reducing body hair, or preventing sexually transmitted infections.

After reading this definition, please tell us if you use this word, or what word you use instead.

- ○ Yes, I use the words "birth control". (1)
- ○ No, I use a different word. The word(s) I use instead of "birth control" is: (0) ____
- ○ Prefer not to say (2)

X→

word_abortion An **abortion** is anything someone does to end a pregnancy, which usually involves taking pills or having a procedure to remove the pregnancy from the body. A successful abortion means the person is no longer pregnant.

After reading this definition, please tell us if you use this word, or what word you use instead.

- ○ Yes, I use the word "abortion". (1)
- ○ No, I use a different word. The word(s) I use instead of "abortion" is: (0) ____
- ○ Prefer not to say (2)

End of Block: Introductory questions/preferred language

Start of Block: Gender affirmation and sexual behavior

gasb_0 In this section, depending on the information you have provided, we may ask you several questions about your lived experience with gender identity and sexual orientation, as well as your experience (if any) with gender-affirming treatment and/or procedures, sexual identity, attraction and behavior, and desires for and beliefs about your own fertility.

out How out as a transgender / nonbinary / genderqueer / gender-expansive person do you consider yourself to be?

- I do not identify as transgender / nonbinary / genderqueer / gender-expansive so this question is not relevant for me (7)
- I am out to myself, but have not shared my gender identity with others (1)
- I choose to share my gender identity with some people, but NOT with my health care providers (2)
- I choose to share my gender identity with some people, INCLUDING my health care providers (3)
- I share my gender identity with all people that I interact with, including new people that I meet (4)
- It varies (5)
- I don't know (6)

legal Have you legally changed your gender on any of the following official documents? **Select all that apply.**

- ☐ ☐ I do not identify as transgender / nonbinary / genderqueer / gender-expansive so this question is not relevant for me (8)
- ☐ My driver's license (1)
- ☐ My passport (2)
- ☐ My birth certificate (3)
- ☐ My health insurance (4)
- ☐ Not listed (please specify): (5) ____
- ☐ ☐ I have not changed my gender on any legal documents, and am not in the process of doing so (7)
- ☐ I am in the process of doing so (6)

ga_1 Have you used any of the following to support gender affirmation (sometimes called gender transition)? Select all that apply.

- [ ] I do not identify as transgender / nonbinary / genderqueer / gender-expansive so this question is not relevant for me (9)
- [ ] Medications to stop or delay the onset of puberty (often called "puberty blockers", usually used by youth) (1)
- [ ] Hormone Treatment / Hormone Replacement Therapy (HRT) (2)
- [ ] Gender affirming genital surger(ies) / reconstructive surger(ies) or bottom surger(ies) (3)
- [ ] Gender affirming surger(ies) elsewhere in the body (4)
- [ ] Professional counseling and/or emotional/psychological therapy (6)
- [ ] None of the above (6)
- [ ] I have tried to get one or more of the above services but have not been able to get ANY of the above services (either due to cost, lack of availability near me, or some other barrier) (7)
- [ ] Prefer not to say (8)

Display This Question:

If ga_1 = Medications to stop or delay the onset of puberty (often called "puberty blockers", usually used by youth)

ga_2 At what age did you first begin to take medication to help stop or delay the start of puberty?

▼ 1 (1) ... Prefer not to say (47)

Display This Question:

If ga_1 = Hormone Treatment / Hormone Replacement Therapy (HRT)

ga_3 At what age did you first begin hormone treatment / hormone replacement therapy (HRT)?

▼ 1 (1) ... Prefer not to say (47)

Display This Question:

If ga_1 = Hormone Treatment / Hormone Replacement Therapy (HRT)

Or ga_1 = Medications to stop or delay the onset of puberty (often called "puberty blockers", usually used by youth)

ga_4 Which hormones or medications for the purposes of gender affirmation (also called gender transition) have you **EVER** taken? Select all that apply.

☐ Cyproterone acetate (sometimes called: CPA or Cyprostat) (1)

☐ Dutasteride (sometimes called: Adovart) (2)

☐ Depo leuprolide or leuprolide acetate (sometimes called: Lupron) (3)

☐ Depo provera injection (sometimes called: "Depo" or medroxyprogesterone acetate) (4)

☐ Estrogen (any type in any formulation such as: gel, injection, patch, pill) (5)

☐ Estradiol valerate (a specific type of estrogen) (6)

☐ Estradiol cypionate (a specific type of estrogen) (7)

☐ Finasteride (sometimes called: Proscar or Propecia) (8)

☐ Histarelin acetate (sometimes called: Vantas or Supprelin) (9)

☐ Progesterone (sometimes called: progestagen or progestins) (10)

☐ Micronized progesterone (sometimes called: Prometrium or Provera) (11)

☐ Spironolactone (sometimes called: "Spiro" or Aldactone) (12)

☐ Testosterone (any type in any formulation such as: gel, injection, patch) (13)

☐ Testosterone cypionate (a specific type of testosterone) (14)

☐ Testosterone enanthate (a specific type of testosterone) (15)

☐ Testosterone undecanoate (a specific type of testosterone) (16)

☐ Another hormone/medication not listed here (please specify) (17)
____

☐ I have (also) taken some other hormone(s)/medication(s), but I am not sure what it is called. (18)

☐ None of the above (19)

☐ Prefer not to say (20)

Display This Question:

If ga_1 = Hormone Treatment / Hormone Replacement Therapy (HRT)

Or ga_1 = Medications to stop or delay the onset of puberty (often called "puberty blockers", usually used by youth)

And If

ga_4 != None of the above

Carry Forward Selected Choices from "ga_4"

X→

ga_5 Of the hormones or medications for the purposes of gender affirmation (also called gender transition) that you ever took, please indicate the hormones or medications that you are **CURRENTLY** taking. Select all that apply.

- ☐ I am not currently taking any hormones or medications for the purposes of gender affirmation (1)
- ☐ Cyproterone acetate (sometimes called: CPA or Cyprostat) (2)
- ☐ Dutasteride (sometimes called: Adovart) (3)
- ☐ Depo leuprolide or leuprolide acetate (sometimes called: Lupron) (4)
- ☐ Depo provera injection (sometimes called: "Depo" or medroxyprogesterone acetate) (5)
- ☐ Estrogen (any type in any formulation such as: gel, injection, patch, pill) (6)
- ☐ Estradiol valerate (a specific type of estrogen) (7)
- ☐ Estradiol cypionate (a specific type of estrogen) (8)
- ☐ Finasteride (sometimes called: Proscar or Propecia) (9)
- ☐ Histarelin acetate (sometimes called: Vantas or Supprelin) (10)
- ☐ Progesterone (sometimes called: progestagen or progestins) (11)
- ☐ Micronized progesterone (sometimes called: Prometrium or Provera) (12)
- ☐ Spironolactone (sometimes called: "Spiro" or Aldactone) (13)
- ☐ Testosterone (any type in any formulation such as: gel, injection, patch) (14)
- ☐ Testosterone cypionate (a specific type of testosterone) (15)
- ☐ Testosterone enanthate (a specific type of testosterone) (16)
- ☐ Testosterone undecanoate (a specific type of testosterone) (17)
- ☐ Another hormone/medication not listed here (please specify) (18) ____

☐ I have (also) taken some other hormone(s)/medication(s), but I am not sure what it is called. (19)

☐ ☐ None of the above (20)

☐ ☐ Prefer not to say (21)

Display This Question:

If ga_4 = Cyproterone acetate (sometimes called: CPA or Cyprostat)

JS X→

ga_6 Please tell us when you **STARTED** taking **cyproterone acetate (sometimes called: CPA or Cyprostat)** for gender affirmation or gender transition. (If you do not know the precise month and year, please estimate.)

	Month	Year
Please Select: (1)	▼ January (1 ... December (12)	▼ 1965 (1 ... 2019 (55)

Display This Question:

If ga_4 = Cyproterone acetate (sometimes called: CPA or Cyprostat)

And Of the hormones or medications for the purposes of gender affirmation (also called gender transit... Cyproterone acetate (sometimes called: CPA or Cyprostat) Is Not Selected

JS X→

ga_7 Please tell us when you **STOPPED** taking **cyproterone acetate (sometimes called: CPA or Cyprostat)** for gender affirmation or gender transition. (If you do not know the precise month and year, please estimate.)

	Month	Year
Please Select: (1)	▼ January (1 ... December (12)	▼ 1965 (1 ... 2019 (55)

Display This Question:

If ga_4 = Dutasteride (sometimes called: Adovart)

JS X→

ga_8 Please tell us when you **STARTED** taking **dutasteride (sometimes called: Adovart)** for gender affirmation or gender transition. (If you do not know the precise month and year, please estimate.)

	Month	Year
Please Select: (1)	▼ January (1 ... December (12)	▼ 1965 (1 ... 2019 (55)

Display This Question:

If ga_4 = Dutasteride (sometimes called: Adovart)

And Of the hormones or medications for the purposes of gender affirmation (also called gender transit... Dutasteride (sometimes called: Adovart) Is Not Selected

JS X→

ga_9 Please tell us when you **STOPPED** taking **dutasteride (sometimes called: Adovart)** for gender affirmation or gender transition. (If you do not know the precise month and year, please estimate.)

	Month	Year
Please Select: (1)	▼ January (1 ... December (12)	▼ 1965 (1 ... 2019 (55)

Display This Question:

If ga_4 = Depo provera injection (sometimes called: "Depo" or medroxyprogesterone acetate)

JS X→

ga_10 Please tell us when you **STARTED** taking **depo leuprolide or leuprolide acetate (sometimes called: Lupron)** for gender affirmation or gender transition. (If you do not know the precise month and year, please estimate.)

	Month	Year

Please Select: (1)	▼ January (1 ... December (12)	▼ 1965 (1 ... 2019 (55)

Display This Question:

If ga_4 = Depo provera injection (sometimes called: "Depo" or medroxyprogesterone acetate)

And Of the hormones or medications for the purposes of gender affirmation (also called gender transit... Depo provera injection (sometimes called: "Depo" or medroxyprogesterone acetate) Is Not Selected

ga_11 Please tell us when you **STOPPED** taking **depo leuprolide or leuprolide acetate (sometimes called: Lupron)** for gender affirmation or gender transition. (If you do not know the precise month and year, please estimate.)

	Month	Year
Please Select: (1)	▼ January (1 ... December (12)	▼ 1965 (1 ... 2019 (55)

Display This Question:

If ga_4 = Depo provera injection (sometimes called: "Depo" or medroxyprogesterone acetate)

ga_12 Please tell us when you **STARTED** taking **depo provera injection (sometimes called: "Depo" or medroxyprogesterone acetate)** for gender affirmation or gender transition. (If you do not know the precise month and year, please estimate.)

	Month	Year
Please Select: (1)	▼ January (1 ... December (12)	▼ 1965 (1 ... 2019 (55)

Display This Question:

If ga_4 = Depo provera injection (sometimes called: "Depo" or medroxyprogesterone acetate)

And Of the hormones or medications for the purposes of gender affirmation (also called gender transit... Depo provera injection (sometimes called: "Depo" or medroxyprogesterone acetate) Is Not Selected

JS X→

ga_13 Please tell us when you **STOPPED** taking **depo provera injection (sometimes called: "Depo" or medroxyprogesterone acetate)** for gender affirmation or gender transition. (If you do not know the precise month and year, please estimate.)

	Month	Year
Please Select: (1)	▼ January (1 ... December (12)	▼ 1965 (1 ... 2019 (55)

Display This Question:

If ga_4 = Estrogen (any type in any formulation such as: gel, injection, patch, pill)

JS X→

ga_14 Please tell us when you **STARTED** taking **estrogen (any type in any formulation such as: gel, injection, patch, pill)** for gender affirmation or gender transition. (If you do not know the precise month and year, please estimate.)

	Month	Year
Please Select: (1)	▼ January (1 ... December (12)	▼ 1965 (1 ... 2019 (55)

Display This Question:

If ga_4 = Estrogen (any type in any formulation such as: gel, injection, patch, pill)

And Of the hormones or medications for the purposes of gender affirmation (also called gender transit... Estrogen (any type in any formulation such as: gel, injection, patch, pill) Is Not Selected

JS X→

ga_15 Please tell us when you **STOPPED** taking **estrogen (any type in any formulation such as: gel, injection, patch, pill)** for gender affirmation or gender transition. (If you do not know the precise month and year, please estimate.)

	Month	Year
Please Select: (1)	▼ January (1 ... December (12)	▼ 1965 (1 ... 2019 (55)

Display This Question:

If ga_4 = Estradiol valerate (a specific type of estrogen)

JS X→

ga_16 Please tell us when you **STARTED** taking **estradiol valerate (a specific type of estrogen)** for gender affirmation or gender transition. (If you do not know the precise month and year, please estimate.)

	Month	Year
Please Select: (1)	▼ January (1 ... December (12)	▼ 1965 (1 ... 2019 (55)

Display This Question:

If ga_4 = Estradiol valerate (a specific type of estrogen)

And Of the hormones or medications for the purposes of gender affirmation (also called gender transit... Estradiol valerate (a specific type of estrogen) Is Not Selected

JS X→

ga_17 Please tell us when you **STOPPED** taking **estradiol valerate (a specific type of estrogen)** for gender affirmation or gender transition. (If you do not know the precise month and year, please estimate.)

	Month	Year
Please Select: (1)	▼ January (1 ... December (12)	▼ 1965 (1 ... 2019 (55)

Display This Question:

If ga_4 = Estradiol cypionate (a specific type of estrogen)

JS X→

ga_18 Please tell us when you **STARTED** taking estradiol cypionate (a specific type of estrogen) for gender affirmation or gender transition. (If you do not know the precise month and year, please estimate.)

	Month	Year
Please Select: (1)	▼ January (1 ... December (12)	▼ 1965 (1 ... 2019 (55)

Display This Question:

If ga_4 = Estradiol cypionate (a specific type of estrogen)

And Of the hormones or medications for the purposes of gender affirmation (also called gender transit... Estradiol cypionate (a specific type of estrogen) Is Not Selected

JS X→

ga_19 Please tell us when you **STOPPED** taking estradiol cypionate (a specific type of estrogen) for gender affirmation or gender transition. (If you do not know the precise month and year, please estimate.)

	Month	Year
Please Select: (1)	▼ January (1 ... December (12)	▼ 1965 (1 ... 2019 (55)

Display This Question:

If ga_4 = Finasteride (sometimes called: Proscar or Propecia)

JS X→

ga_20 Please tell us when you STARTED taking finasteride (sometimes called: Proscar or Propecia) for gender affirmation or gender transition. (If you do not know the precise month and year, please estimate.)

	Month	Year

Please Select: (1)	▼ January (1 ... December (12)	▼ 1965 (1 ... 2019 (55)

Display This Question:

If ga_4 = Finasteride (sometimes called: Proscar or Propecia)

And Of the hormones or medications for the purposes of gender affirmation (also called gender transit... Finasteride (sometimes called: Proscar or Propecia) Is Not Selected

JS X→

ga_21 Please tell us when you STOPPED taking finasteride (sometimes called: Proscar or Propecia) for gender affirmation or gender transition. (If you do not know the precise month and year, please estimate.)

	Month	Year
Please Select: (1)	▼ January (1 ... December (12)	▼ 1965 (1 ... 2019 (55)

Display This Question:

If ga_4 = Histarelin acetate (sometimes called: Vantas or Supprelin)

JS X→

ga_22 Please tell us when you STARTED taking histarelin acetate (sometimes called: Vantas or Supprelin) for gender affirmation or gender transition. (If you do not know the precise month and year, please estimate.)

	Month	Year
Please Select: (1)	▼ January (1 ... December (12)	▼ 1965 (1 ... 2019 (55)

Display This Question:

If ga_4 = Histarelin acetate (sometimes called: Vantas or Supprelin)

And Of the hormones or medications for the purposes of gender affirmation (also called gender transit... Histarelin acetate (sometimes called: Vantas or Supprelin) Is Not Selected

JS X→

ga_23 Please tell us when you STOPPED taking histarelin acetate (sometimes called: Vantas or Supprelin) for gender affirmation or gender transition. (If you do not know the precise month and year, please estimate.)

	Month	Year
Please Select: (1)	▼ January (1 ... December (12)	▼ 1965 (1 ... 2019 (55)

Display This Question:

If ga_4 = Progesterone (sometimes called: progestagen or progestins)

JS X→

ga_24 Please tell us when you STARTED taking progesterone (sometimes called: progestagen or progestins) for gender affirmation or gender transition. (If you do not know the precise month and year, please estimate.)

	Month	Year
Please Select: (1)	▼ January (1 ... December (12)	▼ 1965 (1 ... 2019 (55)

Display This Question:

If ga_4 = Progesterone (sometimes called: progestagen or progestins)

And Of the hormones or medications for the purposes of gender affirmation (also called gender transit... Progesterone (sometimes called: progestagen or progestins) Is Not Selected

JS X→

ga_25 Please tell us when you STOPPED taking progesterone (sometimes called: progestagen or progestins) for gender affirmation or gender transition. (If you do not know the precise month and year, please estimate.)

	Month	Year
Please Select: (1)	▼ January (1 ... December (12)	▼ 1965 (1 ... 2019 (55)

Display This Question:

If ga_4 = Micronized progesterone (sometimes called: Prometrium or Provera)

ga_26 Please tell us when you **STARTED** taking **micronized progesterone (sometimes called: Prometrium or Provera)** for gender affirmation or gender transition. (If you do not know the precise month and year, please estimate.)

	Month	Year
Please Select: (1)	▼ January (1 ... December (12)	▼ 1965 (1 ... 2019 (55)

Display This Question:

If ga_4 = Micronized progesterone (sometimes called: Prometrium or Provera)

And Of the hormones or medications for the purposes of gender affirmation (also called gender transit... Micronized progesterone (sometimes called: Prometrium or Provera) Is Not Selected

ga_27 Please tell us when you **STOPPED** taking **micronized progesterone (sometimes called: Prometrium or Provera)** for gender affirmation or gender transition. (If you do not know the precise month and year, please estimate.)

	Month	Year
Please Select: (1)	▼ January (1 ... December (12)	▼ 1965 (1 ... 2019 (55)

Display This Question:

If ga_4 = Spironolactone (sometimes called: "Spiro" or Aldactone)

JS X→

ga_28 Please tell us when you STARTED taking spironolactone (sometimes called: "Spiro" or Aldactone) for gender affirmation or gender transition. (If you do not know the precise month and year, please estimate.)

	Month	Year
Please Select: (1)	▼ January (1 ... December (12)	▼ 1965 (1 ... 2019 (55)

Display This Question:

If ga_4 = Spironolactone (sometimes called: "Spiro" or Aldactone)

And Of the hormones or medications for the purposes of gender affirmation (also called gender transit... Spironolactone (sometimes called: "Spiro" or Aldactone) Is Not Selected

JS X→

ga_29 Please tell us when you STOPPED taking spironolactone (sometimes called: "Spiro" or Aldactone) for gender affirmation or gender transition. (If you do not know the precise month and year, please estimate.)

	Month	Year
Please Select: (1)	▼ January (1 ... December (12)	▼ 1965 (1 ... 2019 (55)

Display This Question:

If ga_4 = Testosterone (any type in any formulation such as: gel, injection, patch)

JS X→

ga_30 Please tell us when you STARTED taking testosterone (any type in any formulation such as: gel, injection, patch) for gender affirmation or gender transition. (If you do not know the precise month and year, please estimate.)

	Month	Year

Please Select: (1)	▼ January (1 ... December (12)	▼ 1965 (1 ... 2019 (55)

Display This Question:

If ga_4 = Testosterone (any type in any formulation such as: gel, injection, patch)

And Of the hormones or medications for the purposes of gender affirmation (also called gender transit... Testosterone (any type in any formulation such as: gel, injection, patch) Is Not Selected

JS X→

ga_31 Please tell us when you STOPPED taking testosterone (any type in any formulation such as: gel, injection, patch) for gender affirmation or gender transition. (If you do not know the precise month and year, please estimate.)

	Month	Year
Please Select: (1)	▼ January (1 ... December (12)	▼ 1965 (1 ... 2019 (55)

Display This Question:

If ga_4 = Testosterone cypionate (a specific type of testosterone)

JS X→

ga_32 Please tell us when you STARTED taking testosterone cypionate (a specific type of testosterone) for gender affirmation or gender transition. (If you do not know the precise month and year, please estimate.)

	Month	Year
Please Select: (1)	▼ January (1 ... December (12)	▼ 1965 (1 ... 2019 (55)

Display This Question:

If ga_4 = Testosterone cypionate (a specific type of testosterone)

And Of the hormones or medications for the purposes of gender affirmation (also called gender transit... Testosterone cypionate (a specific type of testosterone) Is Not Selected

JS X→

ga_33 Please tell us when you STOPPED taking testosterone cypionate (a specific type of testosterone) for gender affirmation or gender transition. (If you do not know the precise month and year, please estimate.)

	Month	Year
Please Select: (1)	▼ January (1 ... December (12)	▼ 1965 (1 ... 2019 (55)

Display This Question:

If ga_4 = Testosterone enanthate (a specific type of testosterone)

JS X→

ga_34 Please tell us when you STARTED taking testosterone enanthate (a specific type of testosterone) for gender affirmation or gender transition. (If you do not know the precise month and year, please estimate.)

	Month	Year
Please Select: (1)	▼ January (1 ... December (12)	▼ 1965 (1 ... 2019 (55)

Display This Question:

If ga_4 = Testosterone enanthate (a specific type of testosterone)

And Of the hormones or medications for the purposes of gender affirmation (also called gender transit... Testosterone enanthate (a specific type of testosterone) Is Not Selected

JS X→

ga_35 Please tell us when you STOPPED taking testosterone enanthate (a specific type of testosterone) for gender affirmation or gender transition. (If you do not know the precise month and year, please estimate.)

	Month	Year
Please Select: (1)	▼ January (1 ... December (12)	▼ 1965 (1 ... 2019 (55)

Display This Question:

If ga_4 = Testosterone undecanoate (a specific type of testosterone)

JS X→

ga_36 Please tell us when you STARTED taking testosterone undecanoate (a specific type of testosterone) for gender affirmation or gender transition. (If you do not know the precise month and year, please estimate.)

	Month	Year
Please Select: (1)	▼ January (1 ... December (12)	▼ 1965 (1 ... 2019 (55)

Display This Question:

If ga_4 = Testosterone undecanoate (a specific type of testosterone)

And Of the hormones or medications for the purposes of gender affirmation (also called gender transit... Testosterone undecanoate (a specific type of testosterone) Is Not Selected

JS X→

ga_37 Please tell us when you STOPPED taking testosterone undecanoate (a specific type of testosterone) for gender affirmation or gender transition. (If you do not know the precise month and year, please estimate.)

	Month	Year
Please Select: (1)	▼ January (1 ... December (12)	▼ 1965 (1 ... 2019 (55)

Display This Question:

If ga_4 = Another hormone/medication not listed here (please specify)

JS X→

ga_38 Please tell us when you STARTED taking ${ga_4/ChoiceTextEntryValue/17} for gender affirmation or gender transition. (If you do not know the precise month and year, please estimate.)

	Month	Year
Please Select: (1)	▼ January (1 ... December (12)	▼ 1965 (1 ... 2019 (55)

Display This Question:

If ga_4 = Another hormone/medication not listed here (please specify)

And Of the hormones or medications for the purposes of gender affirmation (also called gender transit... Another hormone/medication not listed here (please specify) Is Not Selected

JS X→

ga_39 Please tell us when you STOPPED taking ${ga_4/ChoiceTextEntryValue/17} for gender affirmation or gender transition. (If you do not know the precise month and year, please estimate.)

	Month	Year
Please Select: (1)	▼ January (1 ... December (12)	▼ 1965 (1 ... 2019 (55)

X→

ga_surg_0 Would you like to respond to a set of questions about your interest in and experiences with gender-affirming surgery?

If you select "Yes", you will be asked a set of questions about your interest in and experiences with a number of gender-affirming surgeries. If you select "No", you will skip these questions and proceed with the survey.

◯ Yes (1)

◯ No (0)

Skip To: sa_1 If ga_surg_0 = No

X→

ga_surg_1 Have you had or do you want to have "top surgery"? (Chest reduction or reconstruction)

◯ Have had it (1)

◯ Procedure is scheduled, but have not yet had it (2)

◯ Want procedure, but have not yet scheduled it (3)

◯ Not sure if I want this (4)

◯ Do not want this (5)

Display This Question:

If ga_surg_1 = Have had it

JS X→

ga_surg_2 When did you have your "top surgery"? (If you do not know the precise month and year, please estimate.)

	Month	Year
Please Select: (1)	▼ January (1 ... December (12)	▼ 1965 (1 ... 2019 (120)

Display This Question:

If word_uterus = Yes, I use the word "uterus".

Or word_uterus = Prefer not to say

ga_surg_3_u0 Have you had or do you want a hysterectomy / "hysto"? (This refers to a procedure that removes the uterus, and potentially the cervix as well)

- Have had it (1)
- Procedure is scheduled, but have not yet had it (2)
- Want procedure, but have not yet scheduled it (3)
- Not sure if I want this (4)
- Do not want this (5)

Display This Question:

If word_uterus = No, I use a different word. The word I use instead of "uterus" is:

ga_surg_3_u1 Have you had or do you want a hysterectomy / "hysto"? (This refers to a procedure that removes the ${word_uterus/ChoiceTextEntryValue/2}, and potentially the cervix as well)

- Have had it (1)
- Procedure is scheduled, but have not yet had it (2)
- Want procedure, but have not yet scheduled it (3)
- Not sure if I want this (4)
- Do not want this (5)

Display This Question:

If ga_surg_3_u0 = Have had it

Or ga_surg_3_u1 = Have had it

JS X→

ga_surg_3_time When did you have your hysterectomy/"hysto"? (If you do not know the precise month and year, please estimate.)

	Month	Year
Please Select: (1)	▼ January (1 ... December (12)	▼ 1965 (1 ... 2019 (55)

Display This Question:

If ga_surg_3_u0 = Have had it

Or ga_surg_3_u1 = Have had it

X→

ga_surg_3_cervix When you had your hysterectomy/"hysto", was your cervix removed?

- No (0)
- Yes (1)
- I don't know (2)

Display This Question:

If word_penis = Yes, I use the word "penis".

Or word_penis = Prefer not to say

X→

ga_surg_4_pen0 Have you had or do you want to have a metoidioplasty / "meta"? (The creation of a penis by clitoral release, with or without scrotum formation or urethral lengthening)

◯ Have had it (1)

◯ Procedure is scheduled, but have not yet had it (2)

◯ Want procedure, but have not yet scheduled it (3)

◯ Not sure if I want this (4)

◯ Do not want this (5)

Display This Question:

If word_penis = No, I use a different word. The word I use instead of "penis" is:

ga_surg_4_pen1 Have you had or do you want to have a metoidioplasty / "meta" ? (The creation of a ${word_penis/ChoiceTextEntryValue/2} by clitoral release, with or without scrotum formation or urethral lengthening)

◯ Have had it (1)

◯ Procedure is scheduled, but have not yet had it (2)

◯ Want procedure, but have not yet scheduled it (3)

◯ Not sure if I want this (4)

◯ Do not want this (5)

Display This Question:

If ga_surg_4_pen0 = Have had it

Or ga_surg_4_pen1 = Have had it

JS

ga_surg_4_time When did you have your metoidioplasty / "meta"? (If you do not know the precise month and year, please estimate.)

	Month	Year

Please Select: (1)	▼ January (1 ... December (12)	▼ 1965 (1 ... 2019 (55)

ga_surg_5 Have you had or do you want to have an oophorectomy? (A procedure to remove the ovaries; the ovary is an organ about the size and shape of a large almond that makes human eggs and also hormones. There are usually two of them, and they are in the pelvic region of the body.)

- Have had one ovary removed (1)
- Have had both ovaries removed (2)
- Procedure to have one or both ovaries is scheduled, but have not yet had it (3)
- Want to have one or both ovaries removed, but have not yet scheduled it (4)
- Not sure if I want this (5)
- Do not want this (6)

Display This Question:

If ga_surg_5 = Have had one ovary removed

Or ga_surg_5 = Have had both ovaries removed

JS X→

ga_surg_5_when When did you have your oophorectomy? (Note, if you had two surgeries to remove first one and then the other ovary, please tell us about your more recent surgery and if you do not know the precise month and year, please estimate.)

	Month	Year
Please Select: (1)	▼ January (1 ... December (12)	▼ 1965 (1 ... 2019 (55)

Display This Question:

If word_penis = Yes, I use the word "penis".

Or word_penis = Prefer not to say

ga_surg_6_pen0 Have you had or do you want to have a phalloplasty? (The creation of a penis)

- Have had it (1)
- Procedure is scheduled, but have not yet had it (2)
- Want procedure, but have not yet scheduled it (3)
- Not sure if I want this (4)
- Do not want this (5)

Display This Question:

If word_penis = No, I use a different word. The word I use instead of "penis" is:

ga_surg_6_pen1 Have you had or do you want to have a phalloplasty? (The creation of a ${word_penis/ChoiceTextEntryValue/2})

- Have had it (1)
- Procedure is scheduled, but have not yet had it (2)
- Want procedure, but have not yet scheduled it (3)
- Not sure if I want this (4)
- Do not want this (5)

Display This Question:

If ga_surg_6_pen0 = Have had it

Or ga_surg_6_pen1 = Have had it

JS X→

ga_surg_6_time When did you have your phalloplasty? (If you do not know the precise month and year, please estimate.)

	Month	Year
Please Select: (1)	▼ January (1 ... December (12)	▼ 1965 (1 ... 2019 (55)

Display This Question:

If word_uterus = Yes, I use the word "uterus".

Or word_uterus = Prefer not to say

X→

ga_surg_7_u0 Have you had or do you want to have a salpingectomy? (A procedure to remove one or both fallopian tubes; the fallopian tubes connect the ovaries—organs that make eggs—to the uterus.)

- ◯ Have had one fallopian tube removed (1)
- ◯ Have had both fallopian tubes removed (2)
- ◯ Procedure to remove one or both fallopian tubes is scheduled, but have not yet had it (3)
- ◯ Want procedure to have one or both fallopian tubes removed, but have not yet scheduled it (4)
- ◯ Not sure if I want this (5)
- ◯ Do not want this (6)

Display This Question:

If word_uterus = No, I use a different word. The word I use instead of "uterus" is:

X→

ga_surg_7_u1 Have you had or do you want to have a salpingectomy? (A procedure to remove one or both fallopian tubes; the fallopian tubes connect the ovaries—organs that make eggs—to the ${word_uterus/ChoiceTextEntryValue/2}.)

- ○ Have had one fallopian tube removed (1)
- ○ Have had both fallopian tubes removed (2)
- ○ Procedure to have one or both fallopian tubes removed is scheduled, but have not yet had it (3)
- ○ Want procedure to have one or both fallopian tubes removed, but have not yet scheduled it (4)
- ○ Not sure if I want this (5)
- ○ Do not want this (6)

Display This Question:

If ga_surg_7_u0 = Have had one fallopian tube removed

Or ga_surg_7_u0 = Have had both fallopian tubes removed

Or ga_surg_7_u1 = Have had one fallopian tube removed

Or ga_surg_7_u1 = Have had both fallopian tubes removed

JS X→

ga_surg_7_time When did you have your salpingectomy? (Note, if you had two surgeries to remove first one and then the other fallopian tube, please tell us about your more recent surgery and if you do not know the precise month and year, please estimate.)

	Month	Year
Please Select: (1)	▼ January (1 ... December (12)	▼ 1965 (1 ... 2019 (55)

---

ga_surg_8 Have you had or do you want to have a scrotoplasty? (A surgery to create a scrotum)

- Have had it (1)
- Procedure is scheduled, but have not yet had it (2)
- Want procedure, but have not yet scheduled it (3)
- Not sure if I want this (4)
- Do not want this (5)

Display This Question:

If ga_surg_8 = Have had it

JS X→

ga_surg_8_time When did you have your scrotoplasty? (If you do not know the precise month and year, please estimate.)

	Month	Year
Please Select: (1)	▼ January (1 ... December (12)	▼ 1965 (1 ... 2019 (55)

Display This Question:

If word_vagina = Yes, I use the word "vagina".

Or word_vagina = Prefer not to say

ga_surg_9_v0 Have you had or do you want to have a vaginectomy? (Surgery to remove all or part of the vagina)

○ Have had it (1)

○ Procedure is scheduled, but have not yet had it (2)

○ Want procedure, but have not yet scheduled it (3)

○ Not sure if I want this (4)

○ Do not want this (5)

Display This Question:

If word_vagina = No, I use a different word. The word I use instead of "vagina" is:

X→

ga_surg_9_v1 Have you had or do you want to have a vaginectomy? (Surgery to remove all or part of the ${word_vagina/ChoiceTextEntryValue/2})

○ Have had it (1)

○ Procedure is scheduled, but have not yet had it (2)

○ Want procedure, but have not yet scheduled it (3)

○ Not sure if I want this (4)

○ Do not want this (5)

Display This Question:

If ga_surg_9_v0 = Have had it

Or ga_surg_9_v1 = Have had it

JS X→

ga_surg_9_time When did you have your vaginectomy? (If you do not know the precise month and year, please estimate.)

	Month	Year

Please Select: (1)	▼ January (1 ... December (12)	▼ 1965 (1 ... 2019 (55)

---

ga_surg_10 Are there other procedures that you **HAVE** had that we have not previously mentioned?

◯ Yes (1)

◯ No (0)

◯ Prefer not to say (2)

Skip To: ga_surg_11 If ga_surg_10 = No

Skip To: ga_surg_11 If ga_surg_10 = Prefer not to say

Display This Question:

If ga_surg_10 = Yes

ga_surg_10_list Please list other procedures you **have had** (List as many as are relevant to you):

- Procedure 1 (1) ____
- Procedure 2 (2) ____
- Procedure 3 (3) ____
- Procedure 4 (4) ____
- Procedure 5 (5) ____
- Procedure 6 (6) ____
- Procedure 7 (7) ____
- Procedure 8 (8) ____
- Procedure 9 (9) ____
- Procedure 10 (10) ____

---

ga_surg_11 Are there other procedures that you **WANT** to have that we have not previously mentioned?

- Yes (1)
- No (0)
- Prefer not to say (2)

Skip To: landing If ga_surg_11 = No

Skip To: landing If ga_surg_11 = Prefer not to say

Display This Question:

If ga_surg_10 = Yes

ga_surg_11_list Please list other procedures you **want to have** (List as many as are relevant to you):

- ○ Procedure 1 (1) ____
- ○ Procedure 2 (2) ____
- ○ Procedure 3 (3) ____
- ○ Procedure 4 (4) ____
- ○ Procedure 5 (5) ____
- ○ Procedure 6 (6) ____
- ○ Procedure 7 (7) ____
- ○ Procedure 8 (8) ____
- ○ Procedure 9 (9) ____
- ○ Procedure 10 (10) ____

---

landing Thank you. Please continue to the next question.

Display This Question:

If word_preg = Yes, I use the word "pregnant".

Or word_preg = Prefer not to say

And If

ga_1 = Medications to stop or delay the onset of puberty (often called "puberty blockers", usually used by youth)

ga_pb_1_preg0 **BEFORE** you started taking **puberty blockers** for gender affirmation, did a health care provider discuss the implications of hormone use on your ability to get pregnant?

- Yes (1)
- No (0)
- I don't remember (2)

Display This Question:

If word_preg = No, I use a different word. The word I use instead of "pregnant" is:

And If

ga_1 = Medications to stop or delay the onset of puberty (often called "puberty blockers", usually used by youth)

ga_pb_1_preg1 **BEFORE** you started taking **puberty blockers** for gender affirmation, did a health care provider discuss the implications of hormone use on your ability to get ${word_preg/ChoiceTextEntryValue/2}?

- Yes (1)
- No (0)
- I don't remember (2)

Display This Question:

If ga_pb_1_preg0 = Yes

ga_pb_2_preg0 What did the health care provider say to you about your ability to get pregnant **before** starting puberty blockers? Select all that apply.

- ☐ That I would be capable of getting pregnant, even after starting puberty blockers (1)
- ☐ That I would not be capable of getting pregnant after starting puberty blockers (2)
- ☐ That they were unsure of whether I would be able to get pregnant after starting puberty blockers (3)
- ☐ Other conversation about ability to get pregnant (please specify): (4) ____
- ☐ They recommended that I look into fertility preservation options if I wanted to have a biological child one day. (5)
- ☐ ☐ I don't remember (6)
- ☐ ☐ None of these (7)

Display This Question:

If ga_pb_1_preg1 = Yes

ga_pb_2_preg1 What did the health care provider say to you about your ability to get ${word_preg/ChoiceTextEntryValue/2} **before** starting puberty blockers? Select all that apply.

☐ That I would be capable of getting ${word_preg/ChoiceTextEntryValue/2}, even after starting puberty blockers (1)

☐ That I would not be capable of getting ${word_preg/ChoiceTextEntryValue/2} after starting puberty blockers (2)

☐ That they were unsure of whether I would be able to get ${word_preg/ChoiceTextEntryValue/2} after starting puberty blockers (3)

☐ Other conversation about ability to get ${word_preg/ChoiceTextEntryValue/2} (please specify): (4) ____

☐ They recommended that I look into fertility preservation options if I wanted to have a biological child one day. (5)

☐ ☐ I don't remember (6)

☐ ☐ None of these (7)

Display This Question:

If ga_1 = Medications to stop or delay the onset of puberty (often called "puberty blockers", usually used by youth)

And If

word_preg = Yes, I use the word "pregnant".

Or word_preg = Prefer not to say

X→

ga_pb_3_preg0 At any point **AFTER** you began **puberty-blockers**, has a health care provider discussed the implications of your puberty-blocker use on your ability to get pregnant?

◯ Yes (1)

◯ No (0)

◯ I don't remember (2)

Display This Question:

If ga_1 = Medications to stop or delay the onset of puberty (often called "puberty blockers", usually used by youth)

And If

word_preg = No, I use a different word. The word I use instead of "pregnant" is:

ga_pb_3_preg1 At any point **AFTER** you began **puberty-blockers**, has a health care provider discussed the implications of your puberty-blocker use on your ability to get ${word_preg/ChoiceTextEntryValue/2}?

- Yes (1)
- No (0)
- I don't remember (2)

Display This Question:

If word_preg = Yes, I use the word "pregnant".

Or word_preg = Prefer not to say

And If

ga_1 = Hormone Treatment / Hormone Replacement Therapy (HRT)

ga_horm_1_preg0 **BEFORE** you started taking **hormones** for gender affirmation, did a health care provider discuss the implications of hormone use on your ability to get pregnant?

- Yes (1)
- No (0)
- I don't remember (2)

Display This Question:

If word_preg = No, I use a different word. The word I use instead of "pregnant" is:

And ga_1 = Hormone Treatment / Hormone Replacement Therapy (HRT)

ga_horm_1_preg1 **BEFORE** you started taking **hormones** for gender affirmation, did a health care provider discuss the implications of hormone use on your ability to get ${word_preg/ChoiceTextEntryValue/2}?

- Yes (1)
- No (0)
- I don't remember (2)

Display This Question:

If ga_horm_1_preg0 = Yes

ga_horm_2_preg0 What did the health care provider say to you about your ability to get pregnant **before** starting hormone use? Select all that apply.

- That I would be capable of getting pregnant, even after starting hormones (1)
- That I would not be capable of getting pregnant after starting hormones (2)
- That they were unsure of whether I would be able to get pregnant after starting hormones (3)
- Other conversation about ability to get pregnant (please specify): (4) ________________
- They recommended that I look into fertility preservation options if I wanted to have a biological child one day. (5)
- I don't remember (6)
- None of these (7)

Display This Question:

If ga_horm_1_preg1 = Yes

ga_horm_2_preg1 What did the health care provider say to you about your ability to get ${word_preg/ChoiceTextEntryValue/2} **before** starting hormone use? Select all that apply.

- That I would be capable of getting ${word_preg/ChoiceTextEntryValue/2} after starting hormone use (1)
- That I would not be capable of getting ${word_preg/ChoiceTextEntryValue/2} after starting hormone use (2)
- That they were unsure of whether I would be able to get ${word_preg/ChoiceTextEntryValue/2} after starting hormone use (3)
- Other conversation about ability to get ${word_preg/ChoiceTextEntryValue/2} (please specify): (4) ____
- They recommended that I look into fertility preservation options if I wanted to have a biological child one day. (5)
- I don't remember (6)
- None of these (7)

Display This Question:

If ga_1 = Hormone Treatment / Hormone Replacement Therapy (HRT)

And If

word_preg = Yes, I use the word "pregnant".

Or word_preg = Prefer not to say

ga_horm_3_preg0 At any point **AFTER** you began gender-affirming hormones, has a health care provider discussed the implications of your gender-affirming hormone use on your ability to get pregnant?

- Yes (1)
- No (0)
- I don't remember (2)

Display This Question:

If ga_1 = Hormone Treatment / Hormone Replacement Therapy (HRT)

And If

word_preg = No, I use a different word. The word I use instead of "pregnant" is:

ga_horm_3_preg1 At any point **AFTER** you began gender-affirming hormones, has a health care provider discussed the implications of your gender-affirming hormone use on your ability to get ${word_preg/ChoiceTextEntryValue/2}?

- Yes (1)
- No (0)
- I don't remember (2)

Display This Question:

If word_preg = Yes, I use the word "pregnant".

Or word_preg = Prefer not to say

And If

ga_1 = Gender affirming genital surger(ies) / reconstructive surger(ies) or bottom surger(ies)

ga_surg_12_preg0 **BEFORE** you had **surgery(ies)** for gender affirmation, did you a health care provider discuss the implications of gender-affirming surgery on your ability to get pregnant?

- Yes (1)
- No (0)
- I don't remember (2)

Display This Question:

If word_preg = No, I use a different word. The word I use instead of "pregnant" is:

And If

ga_1 = Gender affirming genital surger(ies) / reconstructive surger(ies) or bottom surger(ies)

ga_surg_12_preg1 **BEFORE** you had **surgery(ies)** for gender affirmation, did a health care provider discuss the implications of gender-affirming surgery on your ability to get ${word_preg/ChoiceTextEntryValue/2}?

- Yes (1)
- No (0)
- I don't remember (2)

Display This Question:

If ga_surg_12_preg0 = Yes

ga_surg_13_preg0 What did the health care provider say to you about your ability to get pregnant **before** your gender affirming surgery(ies)? Select all that apply.

- That I would be capable of getting pregnant, even after surgery (1)
- That I would not be capable of getting pregnant after surgery (2)
- That they were unsure of whether I would be able to get pregnant after surgery (3)
- Other conversation about ability to get pregnant (please specify): (4) ____
- They recommended that I look into fertility preservation options if I wanted to have a biological child one day. (5)
- I don't remember (6)
- None of these (7)

Display This Question:

If ga_surg_12_preg1 = Yes

ga_surg_13_preg1 What did the health care provider say to you about your ability to get ${word_preg/ChoiceTextEntryValue/2} **before** your gender affirming surgery(ies)? Select all that apply.

- That I would be capable of getting ${word_preg/ChoiceTextEntryValue/2} after surgery (1)
- That I would not be capable of getting ${word_preg/ChoiceTextEntryValue/2} after surgery (2)
- That they were unsure of whether I would be able to get ${word_preg/ChoiceTextEntryValue/2} after surgery (3)
- Other conversation about ability to get ${word_preg/ChoiceTextEntryValue/2} (please specify): (4) ____
- They recommended that I look into fertility preservation options if I wanted to have a biological child one day. (5)
- I don't remember (6)
- None of these (7)

Display This Question:

If ga_1 = Gender affirming genital surger(ies) / reconstructive surger(ies) or bottom surger(ies)

And If

word_preg = Yes, I use the word "pregnant".

Or word_preg = Prefer not to say

ga_surg_14_preg0 At any point **AFTER** you had gender-affirming surgery(ies), has a health care provider discussed the implications of your gender-affirming surgery(ies) on your ability to get pregnant?

- Yes (1)
- No (0)
- I don't remember (2)

Display This Question:

If ga_1 = Gender affirming genital surger(ies) / reconstructive surger(ies) or bottom surger(ies)

And If

word_preg = No, I use a different word. The word I use instead of "pregnant" is:

X→

ga_surg_14_preg1 At any point **AFTER** you had gender-affirming surgery(ies), has a health care provider discussed the implications of your gender-affirming surgery(ies) on your ability to get ${word_preg/ChoiceTextEntryValue/2}?

- Yes (1)
- No (0)
- I don't remember (2)

Page Break

sa_0 The next questions are related to sexual attraction and activity.

sa_1 Which best describes your feelings of sexual attraction to other people? Select all that apply.

- Attracted to women (1)
- Attracted to men (2)
- Attracted to people with non-binary identities (3)
- Not attracted to people of any gender (4)
- Not sure (5)
- Attracted to people of another gender(s) (please specify): (6) ________________

sa_2 Do you consider yourself to be: (Select all that apply)

- ☐ Asexual (1)
- ☐ Bisexual (2)
- ☐ Gay (3)
- ☐ Lesbian (4)
- ☐ Pansexual (5)
- ☐ Queer (6)
- ☐ Questioning (7)
- ☐ Same-gender loving (8)
- ☐ Straight/heterosexual (9)
- ☐ Another sexual orientation (please specify) (10) ____

---

sa_3 We define a "sexual partner" to be anyone with who you are sexually active, which can include kissing, masturbation, oral sex, non-penetrative sex, penetrative sex, or other things.

How many sexual partners have you had in the **LAST 12 MONTHS**?

▼ 0 (1) ... Prefer not to say (104)

Skip To: End of Block If sa_3 = 0

Display This Question:

If sa_3 != 0

sa_4
Thinking about all of your sexual partner(s) from the **LAST 12 MONTHS**, what is/are the gender(s) of your sexual partner(s)? Select all that apply.

- ▢ I didn't have any sexual partners in the LAST 12 MONTHS (1)
- ▢ Agender (2)
- ▢ Cisgender Man (a person that identifies as a man and was assigned male sex at birth) (3)
- ▢ Cisgender Woman (a person that identifies as a woman and was assigned female sex at birth) (4)
- ▢ Genderqueer (5)
- ▢ Non-binary (6)
- ▢ Transgender Man (a person that identifies as a man but was not assigned male sex at birth) (7)
- ▢ Transgender Woman (a person that identifies as a woman but was not assigned female sex at birth) (8)
- ▢ Two-Spirit (9)
- ▢ Additional gender category (please specify): (10) ____
- ▢ ▢ I don't know (11)

Skip To: End of Block If sa_4 = I didn't have any sexual partners in the LAST 12 MONTHS

sa_5 What was the sex assigned at birth for your sexual partner(s) in the **LAST 12 MONTHS**, for instance, on their original birth certificate? Select all that apply.

- ☐ Female (1)
- ☐ Male (2)
- ☐ I don't know (3)

Display This Question:

If word_penis = Yes, I use the word "penis".

And word_vagina = Yes, I use the word "vagina".

And word_sperm = Yes, I use the word "sperm".

Or If

word_penis = Yes, I use the word "penis".

And word_vagina = Yes, I use the word "vagina".

And word_sperm = Prefer not to say

Or If

word_penis = Yes, I use the word "penis".

And word_vagina = Prefer not to say

And word_sperm = Prefer not to say

Or If

word_penis = Prefer not to say

And word_vagina = Prefer not to say

And word_sperm = Prefer not to say

Or If

word_penis = Prefer not to say

And word_vagina = Yes, I use the word "vagina".

And word_sperm = Yes, I use the word "sperm".

Or If

word_penis = Prefer not to say

And word_vagina = Prefer not to say

And word_sperm = Yes, I use the word "sperm".

Or If

word_penis = Prefer not to say

And word_vagina = Yes, I use the word "vagina".

And word_sperm = Prefer not to say

Or If

word_penis = Yes, I use the word "penis".

And word_vagina = Prefer not to say

And word_sperm = Yes, I use the word "sperm".

sa_6_pen0_s0_v0 In the past year, have you had penis-in-vagina sex with anyone who produces sperm?

◯ Yes (1)

◯ No (0)

◯ I don't know (2)

Display This Question:

If word_penis = Yes, I use the word "penis".

And word_vagina = No, I use a different word. The word I use instead of "vagina" is:

And word_sperm = Yes, I use the word "sperm".

Or If

word_penis = Yes, I use the word "penis".

And word_vagina = No, I use a different word. The word I use instead of "vagina" is:

And word_sperm = Prefer not to say

Or If

word_penis = Prefer not to say

And word_vagina = No, I use a different word. The word I use instead of "vagina" is:

And word_sperm = Yes, I use the word "sperm".

Or If

word_penis = Prefer not to say

And word_vagina = No, I use a different word. The word I use instead of "vagina" is:

And word_sperm = Prefer not to say

X→

sa_6_pen0_s0_v1 In the past year, have you had penis-in-${word_vagina/ChoiceTextEntryValue/2} sex with anyone who produces sperm?

◯ Yes (1)

◯ No (2)

◯ I don't know (3)

Display This Question:

If word_penis = No, I use a different word. The word I use instead of "penis" is:

And word_vagina = Yes, I use the word "vagina".

And word_sperm = Yes, I use the word "sperm".

Or If

word_penis = No, I use a different word. The word I use instead of "penis" is:

And word_vagina = Prefer not to say

And word_sperm = Yes, I use the word "sperm".

Or If

word_penis = No, I use a different word. The word I use instead of "penis" is:

And word_vagina = Yes, I use the word "vagina".

And word_sperm = Prefer not to say

Or If

word_penis = No, I use a different word. The word I use instead of "penis" is:

And word_vagina = Prefer not to say

And word_sperm = Prefer not to say

X→

sa_6_pen1_s0_v0 In the past year, have you had ${word_penis/ChoiceTextEntryValue/2}-in-vagina sex with anyone who produces sperm?

- Yes (1)
- No (2)
- I don't know (3)

Display This Question:

If word_penis = Yes, I use the word "penis".

And word_vagina = No, I use a different word. The word I use instead of "vagina" is:

And word_sperm = No, I use a different word. The word I use instead of "sperm" is:

Or If

word_penis = Prefer not to say

And word_vagina = No, I use a different word. The word I use instead of "vagina" is:

And word_sperm = No, I use a different word. The word I use instead of "sperm" is:

X→

sa_6_pen0_s1_v1 In the past year, have you had penis-in-${word_vagina/ChoiceTextEntryValue/2} sex with anyone who produces ${word_sperm/ChoiceTextEntryValue/2}?

- Yes (1)
- No (2)
- I don't know (3)

Display This Question:

If word_penis = No, I use a different word. The word I use instead of "penis" is:

And word_vagina = No, I use a different word. The word I use instead of "vagina" is:

And word_sperm = No, I use a different word. The word I use instead of "sperm" is:

X→

sa_6_pen1_s1_v1 In the past year, have you had ${word_penis/ChoiceTextEntryValue/2}-in-${word_vagina/ChoiceTextEntryValue/2} sex with anyone who produces ${word_sperm/ChoiceTextEntryValue/2}?

- Yes (1)
- No (2)
- I don't know (3)

Display This Question:

If word_penis = No, I use a different word. The word I use instead of "penis" is:

And word_vagina = No, I use a different word. The word I use instead of "vagina" is:

And word_sperm = Yes, I use the word "sperm".

Or If

word_penis = No, I use a different word. The word I use instead of "penis" is:

And word_vagina = No, I use a different word. The word I use instead of "vagina" is:

And word_sperm = Prefer not to say

X→

sa_6_pen1_s0_v1 In the past year, have you had ${word_penis/ChoiceTextEntryValue/2}-in-${word_vagina/ChoiceTextEntryValue/2} sex with anyone who produces sperm?

- Yes (1)
- No (2)
- I don't know (3)

Display This Question:

If word_penis = No, I use a different word. The word I use instead of "penis" is:

And word_vagina = Yes, I use the word "vagina".

And word_sperm = No, I use a different word. The word I use instead of "sperm" is:

Or If

word_penis = No, I use a different word. The word I use instead of "penis" is:

And word_vagina = Prefer not to say

And word_sperm = No, I use a different word. The word I use instead of "sperm" is:

X→

sa_6_pen1_s1_v0 In the past year, have you had ${word_penis/ChoiceTextEntryValue/2}-in-vagina sex with anyone who produces ${word_sperm/ChoiceTextEntryValue/2}?

- Yes (1)
- No (2)
- I don't know (3)

Display This Question:

If word_penis = Yes, I use the word "penis".

And word_vagina = Yes, I use the word "vagina".

And word_sperm = No, I use a different word. The word I use instead of "sperm" is:

Or If

word_penis = Yes, I use the word "penis".

And word_vagina = Prefer not to say

And word_sperm = No, I use a different word. The word I use instead of "sperm" is:

Or If

word_penis = Prefer not to say

And word_vagina = Prefer not to say

And word_sperm = No, I use a different word. The word I use instead of "sperm" is:

Or If

word_penis = Prefer not to say

And word_vagina = Yes, I use the word "vagina".

And word_sperm = No, I use a different word. The word I use instead of "sperm" is:

X→

sa_6_pen0_s1_v0 In the past year, have you had penis-in-vagina sex with anyone who produces ${word_sperm/ChoiceTextEntryValue/2}?

◯ Yes (1)

◯ No (2)

◯ I don't know (3)

End of Block: Gender affirmation and sexual behavior

Start of Block: Birth control

Display This Question:

If word_bc = Yes, I use the words "birth control".

And word_preg = Yes, I use the word "pregnant".

Or If

word_bc = Yes, I use the words "birth control".

And word_preg = Prefer not to say

Or If

word_bc = Prefer not to say

And word_preg = Prefer not to say

Or If

word_bc = Prefer not to say

And word_preg = Yes, I use the word "pregnant".

bc_1_bc0_preg0 Now we would like to ask you a few questions about birth control. As a reminder, "birth control" can be used by people for different things. Some people use birth control to avoid getting pregnant. Other reasons people use birth control include things like gender affirmation, clearing up their skin, reducing body hair, or preventing sexually transmitted infections.

Have you ever used a method of birth control, for any reason?

- ◯ Yes (1)
- ◯ No (0)
- ◯ I don't know (2)

Skip To: End of Block If bc_1_bc0_preg0 = I don't know

Skip To: End of Block If bc_1_bc0_preg0 = No

Display This Question:

If word_bc = Yes, I use the words "birth control".

And word_preg = No, I use a different word. The word I use instead of "pregnant" is:

Or If

word_bc = Prefer not to say

And word_preg = No, I use a different word. The word I use instead of "pregnant" is:

bc_1_bc0_preg1 Now we would like to ask you a few questions about birth control. As a reminder, "birth control" can be used by people for different things. Some people use birth control to avoid getting ${word_preg/ChoiceTextEntryValue/2}. Other reasons people use birth control include things like gender-affirmation, clearing up their skin, reducing body hair, or preventing sexually transmitted infections.

Have you ever used a method of birth control, for any reason?

- Yes (1)
- No (0)
- I don't know (2)

Skip To: End of Block If bc_1_bc0_preg1 = No

Skip To: End of Block If bc_1_bc0_preg1 = I don't know

Display This Question:

If word_bc = No, I use a different word. The word(s) I use instead of "birth control" is:

And word_preg = Yes, I use the word "pregnant".

Or If

word_bc = No, I use a different word. The word(s) I use instead of "birth control" is:

And word_preg = Prefer not to say

X→

bc_1_bc1_preg0 Now we would like to ask you a few questions about ${word_bc/ChoiceTextEntryValue/2}. As a reminder, "${word_bc/ChoiceTextEntryValue/2}" can be used by people for different things. Some people use ${word_bc/ChoiceTextEntryValue/2} to avoid getting pregnant. Other reasons people use ${word_bc/ChoiceTextEntryValue/2} include things like gender-affirmation, clearing up their skin, reducing body hair, or preventing sexually transmitted infections.

Have you ever used a method of ${word_bc/ChoiceTextEntryValue/2}, for any reason?

- Yes (1)
- No (0)
- I don't know (2)

Skip To: End of Block If bc_1_bc1_preg0 = No

Skip To: End of Block If bc_1_bc1_preg0 = I don't know

Display This Question:

If word_bc = No, I use a different word. The word(s) I use instead of "birth control" is:

And word_preg = No, I use a different word. The word I use instead of "pregnant" is:

bc_1_bc1_preg1 Now we would like to ask you a few questions about ${word_bc/ChoiceTextEntryValue/2}. As a reminder, "${word_bc/ChoiceTextEntryValue/2}" can be used by people for different things. Some people use ${word_bc/ChoiceTextEntryValue/2} to avoid getting ${word_preg/ChoiceTextEntryValue/2}. Other reasons people use ${word_bc/ChoiceTextEntryValue/2} include things like gender-affirmation, clearing up their skin, reducing body hair, or preventing sexually transmitted infections.

Have you ever used a method of ${word_bc/ChoiceTextEntryValue/2}, for any reason?

- Yes (1)
- No (0)
- I don't know (2)

Skip To: End of Block If bc_1_bc1_preg1 = No

Skip To: End of Block If bc_1_bc1_preg1 = I don't know

Display This Question:

If word_bc = Yes, I use the words "birth control".

And word_period = Yes, I use the word "period".

And word_preg = Yes, I use the word "pregnant".

Or If

word_bc = Yes, I use the words "birth control".

And word_period = Yes, I use the word "period".

And word_preg = Prefer not to say

Or If

word_bc = Yes, I use the words "birth control".

And word_period = Prefer not to say

And word_preg = Prefer not to say

Or If

word_bc = Prefer not to say

And word_period = Prefer not to say

And word_preg = Prefer not to say

Or If

word_bc = Prefer not to say

And word_period = Yes, I use the word "period".

And word_preg = Yes, I use the word "pregnant".

Or If

word_bc = Prefer not to say

And word_period = Prefer not to say

And word_preg = Yes, I use the word "pregnant".

Or If

word_bc = Prefer not to say

And word_period = Yes, I use the word "period".

And word_preg = Prefer not to say

Or If

word_bc = Yes, I use the words "birth control".

And word_period = Prefer not to say

And word_preg = Yes, I use the word "pregnant".

bc_2_bc0_per0_preg0 What are the reasons that you have used birth control? Select all that apply.

- ☐ To affirm my gender (1)
- ☐ To avoid getting a sexually transmitted infection (STI) from someone else (2)
- ☐ To avoid spreading a sexually transmitted infection (STI) that I have (3)
- ☐ To avoid symptoms associated with my period like: chest tenderness, bloating, acne, pain from cramping, heavy bleeding (sometimes referred to as pre-menstrual syndrome or PMS) (4)
- ☐ To stop having a period (5)
- ☐ To prevent pregnancy (6)
- ☐ Prevent hair growth (hirsutism) (7)
- ☐ To reduce chronic pelvic pain (including endometriosis) (8)
- ☐ To treat another medical condition (9)
- ☐ Not listed (please specify): (10) ____
- ☐ ☐ None of these (11)

Display This Question:

If word_bc = No, I use a different word. The word(s) I use instead of "birth control" is:

And word_period = Yes, I use the word "period".

And word_preg = Yes, I use the word "pregnant".

Or If

word_bc = No, I use a different word. The word(s) I use instead of "birth control" is:

And word_period = Prefer not to say

And word_preg = Yes, I use the word "pregnant".

Or If

word_bc = No, I use a different word. The word(s) I use instead of "birth control" is:

And word_period = Yes, I use the word "period".

And word_preg = Prefer not to say

Or If

word_bc = No, I use a different word. The word(s) I use instead of "birth control" is:

And word_period = Prefer not to say

And word_preg = Prefer not to say

bc_2_bc1_per0_preg0 What are the reasons that you have used ${word_bc/ChoiceTextEntryValue/2}? Select all that apply.

- To affirm my gender (1)
- To avoid getting a sexually transmitted infection (STI) from someone else (2)
- To avoid spreading a sexually transmitted infection (STI) that I have (3)
- To avoid symptoms associated with my period like: chest tenderness, bloating, acne, pain from cramping, heavy bleeding (sometimes referred to as pre-menstrual syndrome or PMS) (4)
- To stop having a period (5)
- To prevent pregnancy (6)
- Prevent hair growth (hirsutism) (7)
- To reduce chronic pelvic pain (including endometriosis) (8)
- To treat another medical condition (9)
- Not listed (please specify): (10) ____
- None of these (11)

Display This Question:

If word_bc = No, I use a different word. The word(s) I use instead of "birth control" is:

And word_period = No, I use a different word. The word I use instead of "period" is:

And word_preg = Yes, I use the word "pregnant".

Or If

word_bc = No, I use a different word. The word(s) I use instead of "birth control" is:

And word_period = No, I use a different word. The word I use instead of "period" is:

And word_preg = Prefer not to say

bc_2_bc1_per1_preg0 What are the reasons that you have used ${word_bc/ChoiceTextEntryValue/2}? Select all that apply.

- [ ] To affirm my gender (1)
- [ ] To avoid getting a sexually transmitted infection (STI) from someone else (2)
- [ ] To avoid spreading a sexually transmitted infection (STI) that I have (3)
- [ ] To avoid symptoms associated with my ${word_period/ChoiceTextEntryValue/2} like: chest tenderness, bloating, acne, pain from cramping, heavy bleeding (sometimes referred to as pre-menstrual syndrome or PMS) (4)
- [ ] To stop having a ${word_period/ChoiceTextEntryValue/2} (5)
- [ ] To prevent pregnancy (6)
- [ ] Prevent hair growth (hirsutism) (7)
- [ ] To reduce chronic pelvic pain (including endometriosis) (8)
- [ ] To treat another medical condition (9)
- [ ] Not listed (please specify): (10) ____
- [ ] ☐ None of these (11)

Display This Question:

If word_bc = No, I use a different word. The word(s) I use instead of "birth control" is:

And word_period = No, I use a different word. The word I use instead of "period" is:

And word_preg = No, I use a different word. The word I use instead of "pregnant" is:

bc_2_bc1_per1_preg1 What are the reasons that you have used ${word_bc/ChoiceTextEntryValue/2}? Select all that apply.

- ☐ To affirm my gender (1)
- ☐ To avoid getting a sexually transmitted infection (STI) from someone else (2)
- ☐ To avoid spreading a sexually transmitted infection (STI) that I have (3)
- ☐ To avoid symptoms associated with my ${word_period/ChoiceTextEntryValue/2} like: chest tenderness, bloating, acne, pain from cramping, heavy bleeding (sometimes referred to as pre-menstrual syndrome or PMS) (4)
- ☐ To stop having a ${word_period/ChoiceTextEntryValue/2} (5)
- ☐ To prevent ${word_preg/ChoiceTextEntryValue/2} (6)
- ☐ To prevent hair growth (hirsutism) (7)
- ☐ To reduce chronic pelvic pain (including endometriosis) (8)
- ☐ To treat another medical condition (9)
- ☐ Not listed (please specify): (10) ____
- ☐ ☐ None of these (11)

Display This Question:

If word_bc = No, I use a different word. The word(s) I use instead of "birth control" is:

And word_period = Yes, I use the word "period".

And word_preg = No, I use a different word. The word I use instead of "pregnant" is:

Or If

word_bc = No, I use a different word. The word(s) I use instead of "birth control" is:

And word_period = Prefer not to say

And word_preg = No, I use a different word. The word I use instead of "pregnant" is:

bc_2_bc1_per0_preg1 What are the reasons that you have used ${word_bc/ChoiceTextEntryValue/2}? Select all that apply.

☐ To affirm my gender (1)

☐ To avoid getting a sexually transmitted infection (STI) from someone else (2)

☐ To avoid spreading a sexually transmitted infection (STI) that I have (3)

☐ To avoid symptoms associated with my period like: chest tenderness, bloating, acne, pain from cramping, heavy bleeding (sometimes referred to as pre-menstrual syndrome or PMS) (4)

☐ To stop having a period (5)

☐ To prevent ${word_preg/ChoiceTextEntryValue/2} (6)

☐ To prevent hair growth (hirsutism) (7)

☐ To reduce chronic pelvic pain (including endometriosis) (8)

☐ To treat another medical condition (9)

☐ Not listed (please specify): (10)

____

☐ ☐ None of these (11)

Display This Question:

If word_bc = Yes, I use the words "birth control".

And word_period = Yes, I use the word "period".

And word_preg = No, I use a different word. The word I use instead of "pregnant" is:

Or If

word_bc = Yes, I use the words "birth control".

And word_period = Prefer not to say

And word_preg = No, I use a different word. The word I use instead of "pregnant" is:

Or If

word_bc = Prefer not to say

And word_period = Prefer not to say

And word_preg = No, I use a different word. The word I use instead of "pregnant" is:

Or If

word_bc = Prefer not to say

And word_period = Yes, I use the word "period".

And word_preg = No, I use a different word. The word I use instead of "pregnant" is:

X→

bc_2_bc0_per0_preg1 What are the reasons that you have used birth control? Select all that apply.

- ☐ To affirm my gender (1)
- ☐ To avoid getting a sexually transmitted infection (STI) from someone else (2)
- ☐ To avoid spreading a sexually transmitted infection (STI) that I have (3)
- ☐ To avoid symptoms associated with my period like: chest tenderness, bloating, acne, pain from cramping, heavy bleeding (sometimes referred to as pre-menstrual syndrome or PMS) (4)
- ☐ To stop having a period (5)
- ☐ To prevent ${word_preg/ChoiceTextEntryValue/2} (6)
- ☐ To prevent hair growth (hirsutism) (7)
- ☐ To reduce chronic pelvic pain (including endometriosis) (8)
- ☐ To treat another medical condition (9)
- ☐ Not listed (please specify): (10) ____
- ☐ ☐ None of these (11)

Display This Question:

If word_bc = Yes, I use the words "birth control".

And word_period = No, I use a different word. The word I use instead of "period" is:

And word_preg = No, I use a different word. The word I use instead of "pregnant" is:

Or If

word_bc = Prefer not to say

And word_period = No, I use a different word. The word I use instead of "period" is:

And word_preg = No, I use a different word. The word I use instead of "pregnant" is:

bc_2_bc0_per1_preg1 What are the reasons that you have used birth control? Select all that apply.

- [ ] To affirm my gender (1)
- [ ] To avoid getting a sexually transmitted infection (STI) from someone else (2)
- [ ] To avoid spreading a sexually transmitted infection (STI) that I have (3)
- [ ] To avoid symptoms associated with my ${word_period/ChoiceTextEntryValue/2} like: chest tenderness, bloating, acne, pain from cramping, heavy bleeding (sometimes referred to as pre-menstrual syndrome or PMS) (4)
- [ ] To stop having a ${word_period/ChoiceTextEntryValue/2} (5)
- [ ] To prevent ${word_preg/ChoiceTextEntryValue/2} (6)
- [ ] To prevent hair growth (hirsutism) (7)
- [ ] To reduce chronic pelvic pain (including endometriosis) (8)
- [ ] To treat another medical condition (9)
- [ ] Not listed (please specify): (10) ____
- [ ] ☐ None of these (11)

Display This Question:

If word_bc = Yes, I use the words "birth control".

And word_period = No, I use a different word. The word I use instead of "period" is:

And word_preg = Yes, I use the word "pregnant".

Or If

word_bc = Yes, I use the words "birth control".

And word_period = No, I use a different word. The word I use instead of "period" is:

And word_preg = Prefer not to say

Or If

word_bc = Prefer not to say

And word_period = No, I use a different word. The word I use instead of "period" is:

And word_preg = Yes, I use the word "pregnant".

Or If

word_bc = Prefer not to say

And word_period = No, I use a different word. The word I use instead of "period" is:

And word_preg = Prefer not to say

bc_2_bc0_per1_preg0 What are the reasons that you have used birth control? Select all that apply.

☐ To affirm my gender (1)

☐ To avoid getting a sexually transmitted infection (STI) from someone else (2)

☐ To avoid spreading a sexually transmitted infection (STI) that I have (3)

☐ To avoid symptoms associated with my ${word_period/ChoiceTextEntryValue/2} like: chest tenderness, bloating, acne, pain from cramping, heavy bleeding (sometimes referred to as pre-menstrual syndrome or PMS) (4)

☐ To stop having a ${word_period/ChoiceTextEntryValue/2} (5)

☐ To prevent pregnancy (6)

☐ To prevent hair growth (hirsutism) (7)

☐ To reduce chronic pelvic pain (including endometriosis) (8)

☐ To treat another medical condition (9)

☐ Not listed (please specify): (10) ____

☐ ☐ None of these (11)

Page Break

Display This Question:

If bc_2_bc0_per0_preg0 = To prevent pregnancy

Or bc_2_bc1_per0_preg0 = To prevent pregnancy

Or bc_2_bc1_per1_preg0 = To prevent pregnancy

Or bc_2_bc1_per1_preg1 = To prevent ${q://QID28/ChoiceTextEntryValue/2}

Or bc_2_bc1_per0_preg1 = To prevent ${q://QID28/ChoiceTextEntryValue/2}

Or bc_2_bc0_per0_preg1 = To prevent ${q://QID28/ChoiceTextEntryValue/2}

Or bc_2_bc0_per1_preg1 = To prevent ${q://QID28/ChoiceTextEntryValue/2}

Or bc_2_bc0_per1_preg0 = To prevent pregnancy

X→

bc_3 Due to the limitations in electronic survey design and the many options below, we are unable to use your preferred words for this question and will instead use medical terms.

Which methods of birth control/pregnancy prevention have you **EVER** used **FOR THE PURPOSE OF PREGNANCY PREVENTION**? **Select all that apply.**

▢ **Abstinence** (not having sexual contact that could result in pregnancy) (1)

▢ **Cervical Cap** (silicone cup inserted into the vagina to cover the cervix and prevent sperm from entering the uterus) (2)

▢ **Condom** (external, worn on the penis) (3)

▢ **Condom** (internal, worn in the vagina) (4)

▢ **Diaphragm** (shallow, dome-shaped silicone cup inserted into the vagina to cover the cervix and prevent sperm from entering the uterus) (5)

▢ **Emergency Contraception** ("the morning after pill", or Plan B) (6)

▢ **Fertility awareness** (such as the calendar method or ovulation tracking) (7)

▢ **IUD (copper)** (also called ParaGard; a small T-shaped device inserted into the uterus to prevent sperm from fertilizing an egg; effective for up to 10 years) (8)

▢ **IUD (hormonal)** (some examples are Mirena, Skyla, Liletta, and Kyleena; a small T-shaped plastic device inserted into the uterus that releases small amounts of progestin hormone to thicken cervical mucus to prevent sperm from entering the uterus; lasts between 3-5 years) (9)

▢ **Spermicide** (cream, film, foam, gel, or suppository inserted into the vagina that contains chemicals to prevent sperm from moving and reaching the uterus) (10)

▢ **Sterilization (I'm sterilized, or I've had my tubes tied, ovaries removed, and/or uterus removed or other procedure which makes getting pregnant impossible)** (sterilization involves a surgical procedure that closes or blocks the fallopian tubes so eggs and sperm cannot meet and result in pregnancy) (11)

☐ **Sterilization (My partner(s) is/are sterilized)** (sterilization involves a surgical procedure called a "vasectomy" that blocks the tubes that carry sperm, preventing them from reaching the seminal fluid) (12)

☐ **The Implant** (also called Nexplanon, Implanon, or "the rod", is a very small rod that is inserted under the skin of the upper arm that lasts for up to 3 years; it releases a hormone, progestin, that stops the ovaries from releasing eggs) (13)

☐ **The Patch** (a thin, bandage-like piece of sticky plastic that gets placed on the skin and replaced once a week; it releases hormones that thicken cervical mucus and prevent the ovaries from releasing eggs) (14)

☐ **The Pill (combined)** (a pill taken daily by mouth that releases estrogen and progestin types of hormones to stop the ovaries from releasing eggs) (15)

☐ **The Pill (progestin-only)** (also called the "mini-pill"; it is a pill taken daily by the mouth that does not contain estrogen, and instead releases only one type of hormone, progestin, that works by stopping the ovaries from releasing eggs) (16)

☐ **The Ring** (also called NuvaRing; it is a small flexible ring that gets inserted into the vagina that releases hormones that prevent ovaries from releasing eggs) (17)

☐ **The Shot** (also called Depo or Depo-Provera; it is a dose of a hormone, progestin, that lasts for three months and prevents ovaries from releasing eggs) (18)

☐ **The Sponge** (a small, round, piece of plastic foam that you insert into the vagina before sex; it blocks the cervix to prevent sperm from entering and also releases a substance called spermicide that prevents sperm from moving so that they cannot reach the uterus) (19)

☐ **Withdrawal/Pull-Out** (the practice of removing the penis from the vagina before ejaculation occurs so that sperm do not enter the vagina and reach the cervix or uterus) (20)

☐ **Not listed (please specify):** (21) ____

Page Break

Display This Question:

If bc_3 = Abstinence (not having sexual contact that could result in pregnancy)

Or bc_3 = Cervical Cap (silicone cup inserted into the vagina to cover the cervix and prevent sperm from entering the uterus)

Or bc_3 = Condom (external, worn on the penis)

Or bc_3 = Condom (internal, worn in the vagina)

Or bc_3 = Diaphragm (shallow, dome-shaped silicone cup inserted into the vagina to cover the cervix and prevent sperm from entering the uterus)

Or bc_3 = Emergency Contraception ("the morning after pill", or Plan B)

Or bc_3 = Fertility awareness (such as the calendar method or ovulation tracking)

Or bc_3 = IUD (copper) (also called ParaGard; a small T-shaped device inserted into the uterus to prevent sperm from fertilizing an egg; effective for up to 10 years)

Or bc_3 = IUD (hormonal) (some examples are Mirena, Skyla, Liletta, and Kyleena; a small T-shaped plastic device inserted into the uterus that releases small amounts of progestin hormone to thicken cervical mucus to prevent sperm from entering the uterus; lasts between 3-5 years)

Or bc_3 = Spermicide (cream, film, foam, gel, or suppository inserted into the vagina that contains chemicals to prevent sperm from moving and reaching the uterus)

Or bc_3 = Sterilization (I'm sterilized, or I've had my tubes tied, ovaries removed, and/or uterus removed or other procedure which makes getting pregnant impossible) (sterilization involves a surgical procedure that closes or blocks the fallopian tubes so eggs and sperm cannot meet and result in pregnancy)

Or bc_3 = Sterilization (My partner(s) is/are sterilized) (sterilization involves a surgical procedure called a "vasectomy" that blocks the tubes that carry sperm, preventing them from reaching the seminal fluid)

Or bc_3 = The Implant (also called Nexplanon, Implanon, or "the rod", is a very small rod that is inserted under the skin of the upper arm that lasts for up to 3 years; it releases a hormone, progestin, that stops the ovaries from releasing eggs)

Or bc_3 = The Patch (a thin, bandage-like piece of sticky plastic that gets placed on the skin and replaced once a week; it releases hormones that thicken cervical mucus and prevent the ovaries from releasing eggs)

Or bc_3 = The Pill (combined) (a pill taken daily by mouth that releases estrogen and progestin types of hormones to stop the ovaries from releasing eggs)

Or bc_3 = The Pill (progestin-only) (also called the "mini-pill"; it is a pill taken daily by the mouth that does not contain estrogen, and instead releases only one type of hormone, progestin, that works by stopping the ovaries from releasing eggs)

Or bc_3 = The Ring (also called NuvaRing; it is a small flexible ring that gets inserted into the vagina that releases hormones that prevent ovaries from releasing eggs)

Or bc_3 = The Shot (also called Depo or Depo-Provera; it is a dose of a hormone, progestin, that lasts for three months and prevents ovaries from releasing eggs)

Or bc_3 = The Sponge (a small, round, piece of plastic foam that you insert into the vagina before sex; it blocks the cervix to prevent sperm from entering and also releases a substance called spermicide that prevents sperm from moving so that they cannot reach the uterus)

Or bc_3 = Withdrawal/Pull-Out (the practice of removing the penis from the vagina before ejaculation occurs so that sperm do not enter the vagina and reach the cervix or uterus)

Or bc_3 = Not listed (please specify):

Carry Forward Selected Choices from "bc_3"

bc_4 Of the birth control methods you have ever used **FOR THE PURPOSE OF PREGNANCY PREVENTION**, please select below the method(s) you are **CURRENTLY** using to prevent pregnancy. **Select all that apply.**

Due to the limitations in electronic survey design and the many options below, we are unable to use your preferred words for this question and will instead use medical terms.

☐ ☐ I am not currently using any method of birth control for PREGNANCY PREVENTION (1)

☐ **Abstinence** (not having sexual contact that could result in pregnancy) (2)

☐ **Cervical Cap** (silicone cup inserted into the vagina to cover the cervix and prevent sperm from entering the uterus) (3)

☐ **Condom** (external, worn on the penis) (4)

☐ **Condom** (internal, worn in the vagina) (5)

☐ **Diaphragm** (shallow, dome-shaped silicone cup inserted into the vagina to cover the cervix and prevent sperm from entering the uterus) (6)

☐ **Emergency Contraception** ("the morning after pill", or Plan B) (7)

☐ **Fertility awareness** (such as the calendar method or ovulation tracking) (8)

☐ **IUD (copper)** (also called ParaGard; a small T-shaped device inserted into the uterus to prevent sperm from fertilizing an egg; effective for up to 10 years) (9)

☐ **IUD (hormonal)** (some examples are Mirena, Skyla, Liletta, and Kyleena; a small T-shaped plastic device inserted into the uterus that releases small amounts of progestin hormone to thicken cervical mucus to prevent sperm from entering the uterus; lasts between 3-5 years) (10)

☐ **Spermicide** (cream, film, foam, gel, or suppository inserted into the vagina that contains chemicals to prevent sperm from moving and reaching the uterus) (11)

☐ **Sterilization (I'm sterilized, or I've had my tubes tied, ovaries removed, and/or uterus removed or other procedure which makes getting pregnant impossible)** (sterilization involves a surgical procedure that closes or blocks the fallopian tubes so eggs and sperm cannot meet and result in pregnancy) (12)

☐ **Sterilization (My partner(s) is/are sterilized)** (sterilization involves a surgical procedure called a "vasectomy" that blocks the tubes that carry sperm, preventing them from reaching the seminal fluid) (13)

☐ **The Implant** (also called Nexplanon, Implanon, or "the rod", is a very small rod that is inserted under the skin of the upper arm that lasts for up to 3 years; it releases a hormone, progestin, that stops the ovaries from releasing eggs) (14)

☐ **The Patch** (a thin, bandage-like piece of sticky plastic that gets placed on the skin and replaced once a week; it releases hormones that thicken cervical mucus and prevent the ovaries from releasing eggs) (15)

☐ **The Pill (combined)** (a pill taken daily by mouth that releases estrogen and progestin types of hormones to stop the ovaries from releasing eggs) (16)

☐ **The Pill (progestin-only)** (also called the "mini-pill"; it is a pill taken daily by the mouth that does not contain estrogen, and instead releases only one type of hormone, progestin, that works by stopping the ovaries from releasing eggs) (17)

☐ **The Ring** (also called NuvaRing; it is a small flexible ring that gets inserted into the vagina that releases hormones that prevent ovaries from releasing eggs) (18)

☐ **The Shot** (also called Depo or Depo-Provera; it is a dose of a hormone, progestin, that lasts for three months and prevents ovaries from releasing eggs) (19)

☐ **The Sponge** (a small, round, piece of plastic foam that you insert into the vagina before sex; it blocks the cervix to prevent sperm from entering and also releases a substance called spermicide that prevents sperm from moving so that they cannot reach the uterus) (20)

☐ **Withdrawal/Pull-Out** (the practice of removing the penis from the vagina before ejaculation occurs so that sperm do not enter the vagina and reach the cervix or uterus) (21)

☐ **Not listed (please specify):** (22)

____

Page Break

bc_5

Due to the limitations in electronic survey design and the many options below, we are unable to use your preferred words for the response options to this question and will instead use medical terms.

Which methods of "birth control" have you **EVER** used for **ANY** reason? Note, if you used a method for birth control AND another reason, please complete this question. **Select all that apply.**

- ☐ ☐ **I have only ever used birth control for pregnancy prevention** (1)
- ☐ **Abstinence** (not having sexual contact that could result in pregnancy) (2)
- ☐ **Cervical Cap** (silicone cup inserted into the vagina to cover the cervix and prevent sperm from entering the uterus) (3)
- ☐ **Condom** (external, worn on the penis) (4)
- ☐ **Condom** (internal, worn in the vagina) (5)
- ☐ **Diaphragm** (shallow, dome-shaped silicone cup inserted into the vagina to cover the cervix and prevent sperm from entering the uterus) (6)
- ☐ **Emergency Contraception** ("the morning after pill", or Plan B) (7)
- ☐ **Fertility awareness** (such as the calendar method or ovulation tracking) (8)
- ☐ **IUD (copper)** (also called ParaGard; a small T-shaped device inserted into the uterus to prevent sperm from fertilizing an egg; effective for up to 10 years) (9)
- ☐ **IUD (hormonal)** (some examples are Mirena, Skyla, Liletta, and Kyleena; a small T-shaped plastic device inserted into the uterus that releases small amounts of progestin hormone to thicken cervical mucus to prevent sperm from entering the uterus; lasts between 3-5 years) (10)
- ☐ **Spermicide** (cream, film, foam, gel, or suppository inserted into the vagina that contains chemicals to prevent sperm from moving and reaching the uterus) (11)
- ☐ **Sterilization (I'm sterilized, or I've had my tubes tied, ovaries removed, and/or uterus removed or other procedure which makes getting pregnant impossible)**

(sterilization involves a surgical procedure that closes or blocks the fallopian tubes so eggs and sperm cannot meet and result in pregnancy) (12)

☐ **Sterilization (My partner(s) is/are sterilized)** (sterilization involves a surgical procedure called a "vasectomy" that blocks the tubes that carry sperm, preventing them from reaching the seminal fluid) (13)

☐ **The Implant** (also called Nexplanon, Implanon, or "the rod", is a very small rod that is inserted under the skin of the upper arm that lasts for up to 3 years; it releases a hormone, progestin, that stops the ovaries from releasing eggs) (14)

☐ **The Patch** (a thin, bandage-like piece of sticky plastic that gets placed on the skin and replaced once a week; it releases hormones that thicken cervical mucus and prevent the ovaries from releasing eggs) (15)

☐ **The Pill (combined)** (a pill taken daily by mouth that releases estrogen and progestin types of hormones to stop the ovaries from releasing eggs) (16)

☐ **The Pill (progestin-only)** (also called the "mini-pill"; it is a pill taken daily by the mouth that does not contain estrogen, and instead releases only one type of hormone, progestin, that works by stopping the ovaries from releasing eggs) (17)

☐ **The Ring** (also called NuvaRing; it is a small flexible ring that gets inserted into the vagina that releases hormones that prevent ovaries from releasing eggs) (18)

☐ **The Shot** (also called Depo or Depo-Provera; it is a dose of a hormone, progestin, that lasts for three months and prevents ovaries from releasing eggs) (19)

☐ **The Sponge** (a small, round, piece of plastic foam that you insert into the vagina before sex; it blocks the cervix to prevent sperm from entering and also releases a substance called spermicide that prevents sperm from moving so that they cannot reach the uterus) (20)

☐ **Withdrawal/Pull-Out** (the practice of removing the penis from the vagina before ejaculation occurs so that sperm do not enter the vagina and reach the cervix or uterus) (21)

☐ **Not listed (please specify):** (22) ____

Skip To: bc_7_bc0 If bc_5 = I have only ever used birth control for pregnancy prevention

Page Break

Carry Forward Selected Choices from "bc_5"

bc_6

Due to the limitations in electronic survey design and the many options below, we are unable to use your preferred words for this question and will instead use medical terms.

Of the birth control methods you have ever used for **ANY** reason, please select below the method(s) you are **CURRENTLY** using. **Select all that apply.**

- ☐ ☐ I am not currently using any method of birth control (1)
- ☐ ☐ **I have only ever used birth control for pregnancy prevention** (2)
- ☐ **Abstinence** (not having sexual contact that could result in pregnancy) (3)
- ☐ **Cervical Cap** (silicone cup inserted into the vagina to cover the cervix and prevent sperm from entering the uterus) (4)
- ☐ **Condom** (external, worn on the penis) (5)
- ☐ **Condom** (internal, worn in the vagina) (6)
- ☐ **Diaphragm** (shallow, dome-shaped silicone cup inserted into the vagina to cover the cervix and prevent sperm from entering the uterus) (7)
- ☐ **Emergency Contraception** ("the morning after pill", or Plan B) (8)
- ☐ **Fertility awareness** (such as the calendar method or ovulation tracking) (9)
- ☐ **IUD (copper)** (also called ParaGard; a small T-shaped device inserted into the uterus to prevent sperm from fertilizing an egg; effective for up to 10 years) (10)
- ☐ **IUD (hormonal)** (some examples are Mirena, Skyla, Liletta, and Kyleena; a small T-shaped plastic device inserted into the uterus that releases small amounts of progestin hormone to thicken cervical mucus to prevent sperm from entering the uterus; lasts between 3-5 years) (11)
- ☐ **Spermicide** (cream, film, foam, gel, or suppository inserted into the vagina that contains chemicals to prevent sperm from moving and reaching the uterus) (12)
- ☐ **Sterilization (I'm sterilized, or I've had my tubes tied, ovaries removed, and/or uterus removed or other procedure which makes getting pregnant impossible)** (sterilization involves a surgical procedure that closes or blocks the fallopian tubes so eggs and sperm cannot meet and result in pregnancy) (13)

☐ **Sterilization (My partner(s) is/are sterilized)** (sterilization involves a surgical procedure called a "vasectomy" that blocks the tubes that carry sperm, preventing them from reaching the seminal fluid) (14)

☐ **The Implant** (also called Nexplanon, Implanon, or "the rod", is a very small rod that is inserted under the skin of the upper arm that lasts for up to 3 years; it releases a hormone, progestin, that stops the ovaries from releasing eggs) (15)

☐ **The Patch** (a thin, bandage-like piece of sticky plastic that gets placed on the skin and replaced once a week; it releases hormones that thicken cervical mucus and prevent the ovaries from releasing eggs) (16)

☐ **The Pill (combined)** (a pill taken daily by mouth that releases estrogen and progestin types of hormones to stop the ovaries from releasing eggs) (17)

☐ **The Pill (progestin-only)** (also called the "mini-pill"; it is a pill taken daily by the mouth that does not contain estrogen, and instead releases only one type of hormone, progestin, that works by stopping the ovaries from releasing eggs) (18)

☐ **The Ring** (also called NuvaRing; it is a small flexible ring that gets inserted into the vagina that releases hormones that prevent ovaries from releasing eggs) (19)

☐ **The Shot** (also called Depo or Depo-Provera; it is a dose of a hormone, progestin, that lasts for three months and prevents ovaries from releasing eggs) (20)

☐ **The Sponge** (a small, round, piece of plastic foam that you insert into the vagina before sex; it blocks the cervix to prevent sperm from entering and also releases a substance called spermicide that prevents sperm from moving so that they cannot reach the uterus) (21)

☐ **Withdrawal/Pull-Out** (the practice of removing the penis from the vagina before ejaculation occurs so that sperm do not enter the vagina and reach the cervix or uterus) (22)

☐ **Not listed (please specify):** (23) ____

Page Break

Page Break

Display This Question:

If word_bc = Yes, I use the words "birth control".

Or word_bc = Prefer not to say

And If

ga_1 = Hormone Treatment / Hormone Replacement Therapy (HRT)

bc_7_bc0 Have you ever relied on testosterone ("T") as your primary method of birth control?

- Yes, I am currently relying on testosterone as my primary method of birth control (1)
- Yes, I have relied on testosterone as my primary method of birth control in the past (2)
- No (3)

Display This Question:

If word_bc = No, I use a different word. The word(s) I use instead of "birth control" is:

And If

ga_1 = Hormone Treatment / Hormone Replacement Therapy (HRT)

bc_7_bc1 Have you ever relied on testosterone ("T") as your primary method of ${word_bc/ChoiceTextEntryValue/2}?

- Yes, I am currently relying on testosterone as my primary method of ${word_bc/ChoiceTextEntryValue/2} (1)
- Yes, I have relied on testosterone as my primary method of ${word_bc/ChoiceTextEntryValue/2} in the past (2)
- No (3)

Display This Question:

If word_bc = Yes, I use the words "birth control".

And word_sperm = Yes, I use the word "sperm".

And word_vagina = Yes, I use the word "vagina".

Or If

word_bc = Yes, I use the words "birth control".

And word_sperm = Yes, I use the word "sperm".

And word_vagina = Prefer not to say

Or If

word_bc = Yes, I use the words "birth control".

And word_sperm = Prefer not to say

And word_vagina = Prefer not to say

Or If

word_bc = Prefer not to say

And word_sperm = Prefer not to say

And word_vagina = Prefer not to say

Or If

word_bc = Prefer not to say

And word_sperm = Yes, I use the word "sperm".

And word_vagina = Yes, I use the word "vagina".

Or If

word_bc = Prefer not to say

And word_sperm = Prefer not to say

And word_vagina = Yes, I use the word "vagina".

Or If

word_bc = Prefer not to say

And word_sperm = Yes, I use the word "sperm".

And word_vagina = Prefer not to say

Or If

word_bc = Yes, I use the words "birth control".

And word_sperm = Prefer not to say

And word_vagina = Prefer not to say

bc_8_bc0_s0_v0 How consistently do you use birth control when having sex where sperm is released in/near the vagina?

- I do not have sex where sperm is released in/near the vagina (1)
- Every time (2)
- Most of the time (3)
- Some of the time (4)
- Rarely (5)
- Never (6)
- I or my partner(s) have been sterilized (7)
- I or my partner have been deemed infertile after diagnostic testing (8)
- I don't know (9)

Display This Question:

If word_bc = No, I use a different word. The word(s) I use instead of "birth control" is:

And word_sperm = Yes, I use the word "sperm".

And word_vagina = Yes, I use the word "vagina".

Or If

word_bc = No, I use a different word. The word(s) I use instead of "birth control" is:

And word_sperm = Prefer not to say

And word_vagina = Yes, I use the word "vagina".

Or If

word_bc = No, I use a different word. The word(s) I use instead of "birth control" is:

And word_sperm = Yes, I use the word "sperm".

And word_vagina = Prefer not to say

Or If

word_bc = No, I use a different word. The word(s) I use instead of "birth control" is:

And word_sperm = Prefer not to say

And word_vagina = Prefer not to say

bc_8_bc1_s0_v0 How consistently do you use ${word_bc/ChoiceTextEntryValue/2} when having sex where sperm is released in or near the vagina?

- I do not have sex where sperm is released in/near the vagina (1)
- Every time (2)
- Most of the time (3)
- Some of the time (4)
- Rarely (5)
- Never (6)
- I or my partner(s) have been sterilized (7)
- I or my partner have been deemed infertile after diagnostic testing (8)
- I don't know (9)

Display This Question:

If word_bc = No, I use a different word. The word(s) I use instead of "birth control" is:

And word_sperm = Yes, I use the word "sperm".

And word_vagina = No, I use a different word. The word I use instead of "vagina" is:

Or If

word_bc = No, I use a different word. The word(s) I use instead of "birth control" is:

And word_sperm = Prefer not to say

And word_vagina = No, I use a different word. The word I use instead of "vagina" is:

bc_8_bc1_s0_v1 How consistently do you use ${word_bc/ChoiceTextEntryValue/2} when having sex where sperm is released in or near the ${word_vagina/ChoiceTextEntryValue/2}?

- I do not have sex where sperm is released in/near the ${word_vagina/ChoiceTextEntryValue/2} (1)
- Every time (2)
- Most of the time (3)
- Some of the time (4)
- Rarely (5)
- Never (6)
- I or my partner(s) have been sterilized (7)
- I or my partner have been deemed infertile after diagnostic testing (8)
- I don't know (9)

Display This Question:

If word_bc = No, I use a different word. The word(s) I use instead of "birth control" is:

And word_vagina = No, I use a different word. The word I use instead of "vagina" is:

And word_sperm = No, I use a different word. The word I use instead of "sperm" is:

bc_8_bc1_s1_v1 How consistently do you use ${word_bc/ChoiceTextEntryValue/2} when having sex where ${word_sperm/ChoiceTextEntryValue/2} is released in or near the ${word_vagina/ChoiceTextEntryValue/2}?

- I do not have sex where ${word_sperm/ChoiceTextEntryValue/2} is released in/near the ${word_vagina/ChoiceTextEntryValue/2} (1)
- Every time (2)
- Most of the time (3)
- Some of the time (4)
- Rarely (5)
- Never (6)
- I or my partner(s) have been sterilized (7)
- I or my partner have been deemed infertile after diagnostic testing (8)
- I don't know (9)

Display This Question:

If word_bc = No, I use a different word. The word(s) I use instead of "birth control" is:

And word_vagina = Yes, I use the word "vagina".

And word_sperm = No, I use a different word. The word I use instead of "sperm" is:

Or If

word_bc = No, I use a different word. The word(s) I use instead of "birth control" is:

And word_sperm = No, I use a different word. The word I use instead of "sperm" is:

And word_vagina = Prefer not to say

X→

bc_8_bc1_s1_v0 How consistently do you use ${word_bc/ChoiceTextEntryValue/2} when sex where ${word_sperm/ChoiceTextEntryValue/2} is released in or near the vagina?

- I do not have sex where ${word_sperm/ChoiceTextEntryValue/2} is released in/near the vagina (1)
- Every time (2)
- Most of the time (3)
- Some of the time (4)
- Rarely (5)
- Never (6)
- I or my partner(s) have been sterilized (7)
- I or my partner have been deemed infertile after diagnostic testing (8)
- I don't know (9)

Display This Question:

If word_bc = Yes, I use the words "birth control".

And word_sperm = No, I use a different word. The word I use instead of "sperm" is:

And word_vagina = Yes, I use the word "vagina".

Or If

word_bc = Yes, I use the words "birth control".

And word_sperm = No, I use a different word. The word I use instead of "sperm" is:

And word_vagina = Prefer not to say

Or If

word_bc = Prefer not to say

And word_sperm = No, I use a different word. The word I use instead of "sperm" is:

And word_vagina = Yes, I use the word "vagina".

Or If

word_bc = Prefer not to say

And word_sperm = No, I use a different word. The word I use instead of "sperm" is:

And word_vagina = Prefer not to say

bc_8_bc0_s1_v0 How consistently do you use birth control when having sex where ${word_sperm/ChoiceTextEntryValue/2} is released in or near the vagina?

- I do not have sex where ${word_sperm/ChoiceTextEntryValue/2} is released in/near the vagina (1)
- Every time (2)
- Most of the time (3)
- Some of the time (4)
- Rarely (5)
- Never (6)
- I or my partner(s) have been sterilized (7)
- I or my partner have been deemed infertile after diagnostic testing (8)
- I don't know (9)

Display This Question:

If word_bc = Yes, I use the words "birth control".

And word_vagina = No, I use a different word. The word I use instead of "vagina" is:

And word_sperm = No, I use a different word. The word I use instead of "sperm" is:

Or If

word_bc = Prefer not to say

And word_sperm = No, I use a different word. The word I use instead of "sperm" is:

And word_vagina = No, I use a different word. The word I use instead of "vagina" is:

bc_8_bc0_s1_v1 How consistently do you use birth control when having sex where ${word_sperm/ChoiceTextEntryValue/2} is released in or near the ${word_vagina/ChoiceTextEntryValue/2}?

- I do not have sex where ${word_sperm/ChoiceTextEntryValue/2} is released in/near the ${word_vagina/ChoiceTextEntryValue/2} (1)
- Every time (2)
- Most of the time (3)
- Some of the time (4)
- Rarely (5)
- Never (6)
- I or my partner(s) have been sterilized (7)
- I or my partner have been deemed infertile after diagnostic testing (8)
- I don't know (9)

Display This Question:

If word_bc = Yes, I use the words "birth control".

And word_vagina = No, I use a different word. The word I use instead of "vagina" is:

And word_sperm = Yes, I use the word "sperm".

Or If

word_bc = Yes, I use the words "birth control".

And word_sperm = Prefer not to say

And word_vagina = No, I use a different word. The word I use instead of "vagina" is:

Or If

word_bc = Prefer not to say

And word_sperm = Prefer not to say

And word_vagina = No, I use a different word. The word I use instead of "vagina" is:

Or If

word_bc = Prefer not to say

And word_sperm = Yes, I use the word "sperm".

And word_vagina = No, I use a different word. The word I use instead of "vagina" is:

X→

bc_8_bc0_s0_v1 How consistently do you use birth control when having sex where sperm is released in or near the ${word_vagina/ChoiceTextEntryValue/2}?

- I do not have sex where sperm is released in/near the ${word_vagina/ChoiceTextEntryValue/2} (1)
- Every time (2)
- Most of the time (3)
- Some of the time (4)
- Rarely (5)
- Never (6)
- I or my partner(s) have been sterilized (7)
- I or my partner have been deemed infertile after diagnostic testing (8)
- I don't know (9)

End of Block: Birth control

Start of Block: Current pregnancy

Display This Question:

If word_preg = Yes, I use the word "pregnant".

Or word_preg = Prefer not to say

*

preg_1_preg0 How many times have you been pregnant?

____

Skip To: End of Block If preg_1_preg0 = 0

Display This Question:

If word_preg = No, I use a different word. The word I use instead of "pregnant" is:

*

preg_1_preg1 How many times have you been ${word_preg/ChoiceTextEntryValue/2}?

____

Skip To: End of Block If preg_1_preg1 = 0

Skip To: End of Block If preg_1_preg1 Is Empty

Display This Question:

If word_preg = Yes, I use the word "pregnant".

Or word_preg = Prefer not to say

X→

preg_2_preg0 Are you currently pregnant?

○ Yes (1)

○ No (0)

○ I don't know (2)

Display This Question:

If word_preg = No, I use a different word. The word I use instead of "pregnant" is:

preg_2_preg1 Are you currently ${word_preg/ChoiceTextEntryValue/2}?

- Yes (1)
- No (0)
- I don't know (2)

End of Block: Current pregnancy

Start of Block: Fertility desires

Display This Question:

If word_preg = Yes, I use the word "pregnant".

Or word_preg = Prefer not to say

And If

ga_surg_3_u0 != Have had it

And ga_surg_3_u1 != Have had it

fert_1_preg0 Would you like to get pregnant at some time in the future? If you are currently pregnant, please think about whether you would like to get pregnant again after this pregnancy.

- Yes (1)
- No (0)
- I don't know (2)

Skip To: fert_4_preg0 If fert_1_preg0 = No

Skip To: fert_4_preg0 If fert_1_preg0 = I don't know

Display This Question:

If word_preg = No, I use a different word. The word I use instead of "pregnant" is:

And If

ga_surg_3_u0 != Have had it

And ga_surg_3_u1 != Have had it

fert_1_preg1 Would you like to get ${word_preg/ChoiceTextEntryValue/2} at some time in the future? If you are currently ${word_preg/ChoiceTextEntryValue/2}, please think about whether you would like to get ${word_preg/ChoiceTextEntryValue/2} again after this ${word_preg/ChoiceTextEntryValue/2}.

◯ Yes (1)

◯ No (0)

◯ I don't know (2)

Skip To: fert_4_preg1 If fert_1_preg1 = No

Skip To: fert_4_preg1 If fert_1_preg1 = I don't know

Display This Question:

If word_preg = Yes, I use the word "pregnant".

Or word_preg = Prefer not to say

And If

ga_surg_3_u0 != Have had it

And ga_surg_3_u1 != Have had it

fert_2_preg0 Would you like to get pregnant in the next year?

◯ Yes (1)

◯ No (0)

◯ I don't know (2)

Skip To: fert_4_preg0 If fert_2_preg0 = Yes

Skip To: fert_4_preg0 If fert_2_preg0 = I don't know

Display This Question:

If word_preg = No, I use a different word. The word I use instead of "pregnant" is:

And If

ga_surg_3_u0 != Have had it

And ga_surg_3_u1 != Have had it

fert_2_preg1 Would you like to get ${word_preg/ChoiceTextEntryValue/2} in the next year?

- Yes (1)
- No (0)
- I don't know (2)

Skip To: fert_4_preg1 If fert_2_preg1 = Yes

Skip To: fert_4_preg1 If fert_2_preg1 = I don't know

Display This Question:

If word_preg = Yes, I use the word "pregnant".

Or word_preg = Prefer not to say

And If

ga_surg_3_u0 != Have had it

And ga_surg_3_u1 != Have had it

fert_3_preg0 In how many years would you like to get pregnant?

- Within the next five years (1)
- Six to ten years from now (2)
- More than ten years from now (3)
- I don't know (4)

Display This Question:

If word_preg = No, I use a different word. The word I use instead of "pregnant" is:

And If

ga_surg_3_u1 != Have had it

And ga_surg_3_u0 != Have had it

fert_3_preg1 In how many years would you like to get ${word_preg/ChoiceTextEntryValue/2}?

- Within the next five years (1)
- Six to ten years from now (2)
- More than ten years from now (3)
- I don't know (4)

Display This Question:

If word_preg = Yes, I use the word "pregnant".

Or word_preg = Prefer not to say

And If

ga_surg_3_u0 != Have had it

And ga_surg_3_u1 != Have had it

fert_4_preg0 Do you consider yourself to be at risk for getting pregnant at a time when you do not want to be pregnant (unintended pregnancy)?

- Yes (1)
- No (0)
- I don't know (2)

Display This Question:

If word_preg = No, I use a different word. The word I use instead of "pregnant" is:

And If

ga_surg_3_u0 != Have had it

And ga_surg_3_u1 != Have had it

fert_4_preg1 Do you consider yourself to be at risk for getting ${word_preg/ChoiceTextEntryValue/2} at a time when you do not want to be ${word_preg/ChoiceTextEntryValue/2} (unintended ${word_preg/ChoiceTextEntryValue/2})?

- Yes (1)
- No (0)
- I don't know (2)

Display This Question:

If word_preg = Yes, I use the word "pregnant".

And If

ga_surg_3_u0 != Have had it

And ga_surg_3_u1 != Have had it

fert_5_preg0 Please say a bit more about why you do or do not think you are at risk of getting pregnant when you are not trying/wanting to get pregnant.

____

____

____

____

____

Display This Question:

If word_preg = No, I use a different word. The word I use instead of "pregnant" is:

And If

ga_surg_3_u0 != Have had it

And ga_surg_3_u1 != Have had it

fert_5_preg0 Please say a bit more about why you do or do not think you are at risk of getting ${word_preg/ChoiceTextEntryValue/2} when you are not trying/wanting to get ${word_preg/ChoiceTextEntryValue/2}.

____

____

____

____

____

End of Block: Fertility desires

Start of Block: Pregnancy history

Display This Question:

If word_preg = Yes, I use the word "pregnant".

Or word_preg = Prefer not to say

JS X→

phx_1_preg0 Thinking back to your ${lm://Field/2} pregnancy, in what month and year did you become pregnant? (If you do not know the precise month and year, please estimate.)

	Month	Year
Please Select: (1)	▼ January (1 ... December (12)	▼ 1965 (1 ... 2019 (55)

Display This Question:

If word_preg = Yes, I use the word "pregnant".

Or word_preg = Prefer not to say

phx_2_preg0 Were you trying to get pregnant at that time?

- Yes (1)
- No (0)
- I don't remember (2)

Display This Question:

If word_preg = Yes, I use the word "pregnant".

And word_abortion = Yes, I use the word "abortion".

Or If

word_abortion = Yes, I use the word "abortion".

And word_preg = Prefer not to say

Or If

word_abortion = Prefer not to say

And word_preg = Yes, I use the word "pregnant".

Or If

word_preg = Prefer not to say

And word_abortion = Prefer not to say

phx_3_a0_preg0 What was the outcome of your ${lm://Field/2} pregnancy?

- I am still pregnant (1)
- Abortion (2)
- Ectopic or tubal pregnancy (3)
- Live birth (4)
- Miscarriage (5)
- Stillbirth (6)
- I don't know (7)

Display This Question:

If word_preg = Yes, I use the word "pregnant".

And word_abortion = No, I use a different word. The word(s) I use instead of "abortion" is:

Or If

word_abortion = No, I use a different word. The word(s) I use instead of "abortion" is:

And word_preg = Prefer not to say

phx_3_a1_preg0 What was the outcome of your ${lm://Field/2} pregnancy?

- I am still pregnant (1)
- ${word_abortion/ChoiceTextEntryValue/2} (2)
- Ectopic or tubal pregnancy (3)
- Live birth (4)
- Miscarriage (5)
- Stillbirth (6)
- I don't know (7)

Display This Question:

If Loop current: phx_3_a0_preg0 = Live birth

Or Loop current: phx_3_a1_preg0 = Live birth

phx_4_preg0 Was the baby born via cesarean section ("c"-section)?

◯ Yes (1)

◯ No (0)

End of Block: Pregnancy history

Start of Block: Pregnancy history w piped in language

Display This Question:

If word_preg = No, I use a different word. The word I use instead of "pregnant" is:

phx_1_preg1 Thinking back to your ${lm://Field/2} ${word_preg/ChoiceTextEntryValue/2}, in what month and year did you become ${word_preg/ChoiceTextEntryValue/2}? (If you do not know the precise month and year, please estimate.)

	Month	Year
Please Select: (1)	▼ January (1 ... December (12)	▼ 1965 (1 ... 2019 (55)

Display This Question:

If word_preg = No, I use a different word. The word I use instead of "pregnant" is:

phx_2_preg1 Were you trying to get ${word_preg/ChoiceTextEntryValue/2} at that time?

- Yes (1)
- No (0)
- I don't remember (2)

Display This Question:

If word_preg = No, I use a different word. The word I use instead of "pregnant" is:

And word_abortion = Yes, I use the word "abortion".

Or If

word_abortion = Prefer not to say

And word_preg = No, I use a different word. The word I use instead of "pregnant" is:

phx_3_a0_preg1 What was the outcome of your ${lm://Field/2} ${word_preg/ChoiceTextEntryValue/2}?

- I am still ${word_preg/ChoiceTextEntryValue/2} (1)
- Abortion (2)
- Ectopic or tubal ${word_preg/ChoiceTextEntryValue/2} (3)
- Live birth (4)
- Miscarriage (5)
- Stillbirth (6)
- I don't know (7)

Display This Question:

If word_preg = No, I use a different word. The word I use instead of "pregnant" is:

And word_abortion = No, I use a different word. The word(s) I use instead of "abortion" is:

phx_3_a1_preg1 What was the outcome of your ${lm://Field/2} ${word_preg/ChoiceTextEntryValue/2}?

- I am still ${word_preg/ChoiceTextEntryValue/2} (1)
- ${word_abortion/ChoiceTextEntryValue/2} (2)
- Ectopic or tubal ${word_preg/ChoiceTextEntryValue/2} (3)
- Live birth (4)
- Miscarriage (5)
- Stillbirth (6)
- I don't know (7)

Display This Question:

If Loop current: phx_3_a0_preg1 = Live birth

Or Loop current: phx_3_a1_preg1 = Live birth

X→

phx_4_preg1 Was the baby born via cesarean section ("c"-section)?

- Yes (1)
- No (0)

End of Block: Pregnancy history w piped in language

Start of Block: Pregnancy and health care

Display This Question:

If If How many times have you been pregnant? Text Response Is Greater Than 0

And If

ga_4 = Testosterone (any type in any formulation such as: gel, injection, patch)

Or ga_4 = Testosterone cypionate (a specific type of testosterone)

Or ga_4 = Testosterone enanthate (a specific type of testosterone)

Or ga_4 = Testosterone undecanoate (a specific type of testosterone)

phc_1_preg0 How many of your pregnancies occurred **AFTER** you started taking testosterone?

____

Display This Question:

If If How many times have you been ${q://QID28/ChoiceTextEntryValue/2}? Text Response Is Greater Than 0

And If

ga_4 = Testosterone (any type in any formulation such as: gel, injection, patch)

Or ga_4 = Testosterone cypionate (a specific type of testosterone)

Or ga_4 = Testosterone enanthate (a specific type of testosterone)

Or ga_4 = Testosterone undecanoate (a specific type of testosterone)

phc_1_preg1 How many of your ${word_preg/ChoiceTextEntryValue/2}s occurred **AFTER** you started taking testosterone?

____

Display This Question:

If If How many of your pregnancies occurred AFTER you started taking testosterone? Text Response Is Greater Than 0

phc_2_preg0 Did your healthcare provider discuss the potential interactions/complications of your testosterone use and pregnancy?

- ◯ Yes (1)
- ◯ No (0)
- ◯ I don't remember (2)

Display This Question:

If If How many of your ${q://QID28/ChoiceTextEntryValue/2}s occurred AFTER you started taking testoster... Text Response Is Greater Than 0

phc_2_preg1 Did your healthcare provider discuss the potential interactions/complications of your testosterone use and ${word_preg/ChoiceTextEntryValue/2}?

- ◯ Yes (1)
- ◯ No (0)
- ◯ I don't remember (2)

Display This Question:

If If How many of your pregnancies occurred AFTER you started taking testosterone? Text Response Is Greater Than 0

*

phc_3_preg0 For how many of these pregnancies were you taking testosterone AT THE TIME you got pregnant?

____

Display This Question:

If If How many of your ${q://QID28/ChoiceTextEntryValue/2}s occurred AFTER you started taking testoster... Text Response Is Greater Than 0

*

phc_3_preg1 For how many of these ${word_preg/ChoiceTextEntryValue/2}s were you taking testosterone AT THE TIME you got ${word_preg/ChoiceTextEntryValue/2}?

____

Display This Question:

If If For how many of these pregnancies were you taking testosterone AT THE TIME you got pregnant? Text Response Is Greater Than 0

phc_4_preg0 For the most recent pregnancy that occurred while you were on testosterone, please tell us how long you had been on testosterone at the time you got pregnant.

- <1 month (1)
- 1-3 months (2)
- 4-6 months (3)
- 7-12 months (4)
- 1-2 years (5)
- 3-5 years (6)
- More than 5 years (7)
- I don't remember (8)

Display This Question:

If If For how many of these ${q://QID28/ChoiceTextEntryValue/2}s were you taking testosterone AT THE TI... Text Response Is Greater Than 0

phc_4_preg1 For the most recent ${word_preg/ChoiceTextEntryValue/2} that occurred while you were on testosterone, please tell us for how many **MONTHS** you had been on testosterone at the time you got ${word_preg/ChoiceTextEntryValue/2}.

- <1 month (1)
- 1-3 months (2)
- 4-6 months (3)
- 7-12 months (4)
- 1-2 years (5)
- 3-5 years (6)
- More than 5 years (7)
- I don't remember (8)

Display This Question:

If word_period = Yes, I use the word "period".

And word_preg = Yes, I use the word "pregnant".

And For how many of these pregnancies were you taking testosterone AT THE TIME you got pregnant? Text Response Is Greater Than 0

Or If

word_preg = Prefer not to say

And word_period = Prefer not to say

And For how many of these pregnancies were you taking testosterone AT THE TIME you got pregnant? Text Response Is Greater Than 0

Or If

word_period = Prefer not to say

And word_preg = Yes, I use the word "pregnant".

And For how many of these pregnancies were you taking testosterone AT THE TIME you got pregnant? Text Response Is Greater Than 0

Or If

word_preg = Prefer not to say

And word_period = Yes, I use the word "period".

And For how many of these pregnancies were you taking testosterone AT THE TIME you got pregnant? Text Response Is Greater Than 0

phc_5_per0_preg0 For the most recent pregnancy that occurred while you were using testosterone, please tell us whether you were still having periods at the time that you got pregnant.

- ○ Yes, regular periods (1)
- ○ Yes, irregular periods (2)
- ○ No, I was not getting my period (0)
- ○ I don't remember (3)

Display This Question:

If If For how many of these ${q://QID28/ChoiceTextEntryValue/2}s were you taking testosterone AT THE TI... Text Response Is Greater Than 0

And If

word_period = Yes, I use the word "period".

Or word_period = Prefer not to say

phc_5_per0_preg1 For the most recent ${word_preg/ChoiceTextEntryValue/2} that occurred while you were using testosterone, please tell us whether you were still having periods at the time that you got ${word_preg/ChoiceTextEntryValue/2}.

- Yes, regular periods (1)
- Yes, irregular periods (2)
- No, I was not getting my period (0)
- I don't remember (3)

Display This Question:

If If For how many of these ${q://QID28/ChoiceTextEntryValue/2}s were you taking testosterone AT THE TI... Text Response Is Greater Than 0

And If

word_period = No, I use a different word. The word I use instead of "period" is:

phc_5_per1_preg1 For the most recent ${word_preg/ChoiceTextEntryValue/2} that occurred while you were using testosterone, please tell us whether you were still having ${word_period/ChoiceTextEntryValue/2}s at the time that you got ${word_preg/ChoiceTextEntryValue/2}.

- Yes, regular ${word_period/ChoiceTextEntryValue/2}s (1)
- Yes, irregular ${word_period/ChoiceTextEntryValue/2}s (2)
- No, I was not getting my ${word_period/ChoiceTextEntryValue/2} (0)
- I don't remember (3)

Display This Question:

If If For how many of these pregnancies were you taking testosterone AT THE TIME you got pregnant? Text Response Is Greater Than 0

And word_period = No, I use a different word. The word I use instead of "period" is:

phc_5_per1_preg0 For the most recent pregnancy that occurred while you were using testosterone, please tell us whether you were still having ${word_period/ChoiceTextEntryValue/2}s at the time that you got pregnant.

- Yes, regular ${word_period/ChoiceTextEntryValue/2}s (1)
- Yes, irregular ${word_period/ChoiceTextEntryValue/2}s (2)
- No, I was not getting my ${word_period/ChoiceTextEntryValue/2} (0)
- I don't remember (3)

Display This Question:

If If For how many of these pregnancies were you taking testosterone AT THE TIME you got pregnant? Text Response Is Equal to 0

phc_6_preg0 How many **MONTHS** before getting pregnant did you stop taking testosterone? (For your most recent pregnancy that occurred after starting testosterone).

- Months: (1) ____
- I don't remember (2)

Display This Question:

If If For how many of these ${q://QID28/ChoiceTextEntryValue/2}s were you taking testosterone AT THE TI... Text Response Is Equal to 0

phc_6_preg1 How many **MONTHS** before getting ${word_preg/ChoiceTextEntryValue/2} did you stop taking testosterone? (For your most recent ${word_preg/ChoiceTextEntryValue/2} that occurred after starting testosterone).

○ Months: (1) ____

○ I don't remember (2)

Display This Question:

If If For how many of these pregnancies were you taking testosterone AT THE TIME you got pregnant? Text Response Is Equal to 0

phc_7_preg0 Did you stop taking testosterone **specifically** so that you could get pregnant?

○ No, I stopped testosterone for another reason (please describe): (0)
____

○ Yes, I stopped taking testosterone so that I could try to get pregnant (1)

Display This Question:

If If For how many of these ${q://QID28/ChoiceTextEntryValue/2}s were you taking testosterone AT THE TI... Text Response Is Equal to 0

phc_7_preg1 Did you stop taking testosterone **specifically** so that you could get ${word_preg/ChoiceTextEntryValue/2}?

○ No, I stopped testosterone for another reason (please describe): (0)
____

○ Yes, I stopped taking testosterone so that I could try to get ${word_preg/ChoiceTextEntryValue/2} (1)

Display This Question:

If If How many times have you been pregnant? Text Response Is Greater Than 0

Or How many times have you been ${q://QID28/ChoiceTextEntryValue/2}? Text Response Is Greater Than 0

X→

psa_0 Now we will ask a set of questions that can be upsetting or triggering for some people. These questions relate to physical and sexual assault. We are asking these questions to better understand certain experiences with sexual partners, and to what extent people are at risk for negative outcomes. **You are free to skip these questions for any reason.** If you would like to skip these questions, please select the option to skip below. If you feel comfortable answering these questions, please select the option to continue to the questions.

If reading these questions upsets you, or for any reason you would like to speak to a trained sexual assault service provider in your area, you can contact the National Sexual Assault Telephone hotline at 800.656.HOPE (4673). You can also contact the NYC Anti-Violence Project hotline, a 24-hour, 365-day-a-year hotline that offers support to LGBTQ and HIV-affected survivors of any type of violence, at 212.714.1141. Their website is available here.

- Skip questions (0)
- Continue to questions (1)

Display This Question:

If word_preg = Yes, I use the word "pregnant".

Or word_preg = Prefer not to say

And If

psa_0 = Continue to questions

And How many times have you been pregnant? Text Response Is Greater Than 0

X→

psa_1_preg0 Has any person with whom you got pregnant ever hit, slapped, kicked, or otherwise physically hurt you?

- No (0)
- Yes (1)

Display This Question:

If word_preg = No, I use a different word. The word I use instead of "pregnant" is:

And If

psa_0 = Continue to questions

And How many times have you been ${q://QID28/ChoiceTextEntryValue/2}? Text Response Is Greater Than 0

psa_1_preg1 Has any person with whom you got ${word_preg/ChoiceTextEntryValue/2} ever hit, slapped, kicked, or otherwise physically hurt you?

- No (0)
- Yes (1)

Display This Question:

If If How many times have you been pregnant? Text Response Is Greater Than 0

And psa_0 = Continue to questions

psa_2_preg0 Has a person with whom you got pregnant ever forced you to do anything sexual when you did not want to?

- No (0)
- Yes (1)

Display This Question:

If If How many times have you been ${q://QID28/ChoiceTextEntryValue/2}? Text Response Is Greater Than 0

And psa_0 = Continue to questions

psa_2_preg1 Has a person with whom you got ${word_preg/ChoiceTextEntryValue/2} ever forced you to do anything sexual when you did not want to?

- No (0)
- Yes (1)

Display This Question:

If If How many times have you been pregnant? Text Response Is Greater Than 0

And psa_0 = Continue to questions

psa_3_preg0 Have you ever gotten pregnant as the result of someone forcing you to have sex when you did not want to have sex?

- No (0)
- Yes (1)

Display This Question:

If If How many times have you been ${q://QID28/ChoiceTextEntryValue/2}? Text Response Is Greater Than 0

And psa_0 = Continue to questions

psa_3_preg1 Have you ever gotten ${word_preg/ChoiceTextEntryValue/2} as the result of someone forcing you to have sex when you did not want to have sex?

- No (0)
- Yes (1)

End of Block: Pregnancy and health care

Start of Block: Sexual and reproductive health care

srh_0 Next, please share your opinions of and experiences with several sexual and reproductive health services.

Display This Question:

If word_abortion = Yes, I use the word "abortion".

And word_bc = Yes, I use the words "birth control".

And word_preg = Yes, I use the word "pregnant".

Or If

word_abortion = Yes, I use the word "abortion".

And word_bc = Yes, I use the words "birth control".

And word_preg = Prefer not to say

Or If

word_abortion = Yes, I use the word "abortion".

And word_bc = Prefer not to say

And word_preg = Prefer not to say

Or If

word_abortion = Prefer not to say

And word_bc = Prefer not to say

And word_preg = Prefer not to say

Or If

word_abortion = Prefer not to say

And word_bc = Yes, I use the words "birth control".

And word_preg = Yes, I use the word "pregnant".

Or If

word_abortion = Prefer not to say

And word_bc = Prefer not to say

And word_preg = Yes, I use the word "pregnant".

Or If

word_abortion = Prefer not to say

And word_bc = Yes, I use the words "birth control".

And word_preg = Prefer not to say

Or If

word_abortion = Yes, I use the word "abortion".

And word_bc = Prefer not to say

And word_preg = Yes, I use the word "pregnant".

srh_1_a0_bc0_preg0 From the below list of reproductive health issues facing people who share your sexual orientation and/or gender identity, please select the **three** that feel most important to you.

- ☐ Access to routine reproductive screenings and preventive care (e.g., screening for sexually transmitted infections, pap smears) (1)
- ☐ Fertility preservation (such as egg freezing) (2)
- ☐ Gaps in healthcare provider knowledge (3)
- ☐ Trans and gender expansive affirming abortion care (4)
- ☐ LGBQ+ affirming abortion care (5)
- ☐ Trans and gender expansive-affirming birth control options (6)
- ☐ LGBQ+ affirming birth control options (7)
- ☐ Trans and gender expansive-affirming pregnancy care (8)
- ☐ LGBQ+ affirming pregnancy care (9)
- ☐ Lack of affirming patient education materials (10)
- ☐ Lack of cultural competency from healthcare staff/providers (11)
- ☐ Lack of inclusive/affirming sexual education curricula for use in training health care professionals (12)
- ☐ Medically accurate counseling about pregnancy risk (13)
- ☐ Transgender and gender expansive individuals' knowledge of basic health & health care issues (14)
- ☐ LGBQ+ individuals' knowledge of basic health & health care issues (15)
- ☐ Not listed (please specify): (16) ____
- ☐ ☐ None of these (17)

Display This Question:

If word_abortion = Yes, I use the word "abortion".

And word_bc = No, I use a different word. The word(s) I use instead of "birth control" is:

And word_preg = Yes, I use the word "pregnant".

Or If

word_abortion = Yes, I use the word "abortion".

And word_bc = No, I use a different word. The word(s) I use instead of "birth control" is:

And word_preg = Prefer not to say

Or If

word_abortion = Prefer not to say

And word_bc = No, I use a different word. The word(s) I use instead of "birth control" is:

And word_preg = Yes, I use the word "pregnant".

Or If

word_abortion = Prefer not to say

And word_bc = No, I use a different word. The word(s) I use instead of "birth control" is:

And word_preg = Prefer not to say

srh_1_a0_bc1_preg0 From the below list of reproductive health issues facing people who share your sexual orientation and/or gender identity, please select the **three** that feel most important to you.

- ☐ Access to routine reproductive screenings and preventive care (e.g., screening for sexually transmitted infections, pap smears) (1)
- ☐ Fertility preservation (such as egg freezing) (2)
- ☐ Gaps in healthcare provider knowledge (3)
- ☐ Trans and gender-expansive affirming abortion care (4)
- ☐ LGBQ+ affirming abortion care (5)
- ☐ Trans and gender-expansive affirming ${word_bc/ChoiceTextEntryValue/2} options (6)
- ☐ LGBQ+ affirming ${word_bc/ChoiceTextEntryValue/2} options (7)
- ☐ Trans and gender-expansive affirming pregnancy care (8)
- ☐ LGBQ+ affirming pregnancy care (9)
- ☐ Lack of affirming patient education materials (10)
- ☐ Lack of cultural competency from healthcare staff/providers (11)
- ☐ Lack of inclusive/affirming sexual education curricula for use in training health care professionals (12)
- ☐ Medically accurate counseling about pregnancy risk (13)
- ☐ Transgender and gender expansive individuals’ knowledge of basic health & health care issues (14)
- ☐ LGBQ+ individuals’ knowledge of basic health & health care issues (15)
- ☐ Not listed (please specify): (16) ____
- ☐ ☐ None of these (17)

Display This Question:

If word_abortion = Yes, I use the word "abortion".

And word_bc = No, I use a different word. The word(s) I use instead of "birth control" is:

And word_preg = No, I use a different word. The word I use instead of "pregnant" is:

Or If

word_abortion = Prefer not to say

And word_bc = No, I use a different word. The word(s) I use instead of "birth control" is:

And word_preg = No, I use a different word. The word I use instead of "pregnant" is:

srh_1_a0_bc1_preg1 From the below list of reproductive health issues facing people who share your sexual orientation and/or gender identity, please select the **three** that feel most important to you.

- ☐ Access to routine reproductive screenings and preventive care (e.g., screening for sexually transmitted infections, pap smears) (1)
- ☐ Fertility preservation (such as egg freezing) (2)
- ☐ Gaps in healthcare provider knowledge (3)
- ☐ Trans and gender-expansive affirming abortion care (4)
- ☐ LGBQ+ affirming abortion care (5)
- ☐ Trans and gender-expansive affirming ${word_bc/ChoiceTextEntryValue/2} options (6)
- ☐ LGBQ+ affirming ${word_bc/ChoiceTextEntryValue/2} options (7)
- ☐ Trans and gender-expansive affirming ${word_preg/ChoiceTextEntryValue/2} care (8)
- ☐ LGBQ+ affirming ${word_preg/ChoiceTextEntryValue/2} care (9)
- ☐ Lack of affirming patient education materials (10)
- ☐ Lack of cultural competency from healthcare staff/providers (11)
- ☐ Lack of inclusive/affirming sexual education curricula for use in training health care professionals (12)
- ☐ Medically accurate counseling about ${word_preg/ChoiceTextEntryValue/2} risk (13)
- ☐ Transgender and gender expansive individuals' knowledge of basic health & health care issues (14)
- ☐ LGBQ+ individuals' knowledge of basic health & health care issues (15)
- ☐ Not listed (please specify): (16) ____
- ☐ ☐ None of these (17)

Display This Question:

If word_bc = No, I use a different word. The word(s) I use instead of "birth control" is:

And word_preg = No, I use a different word. The word I use instead of "pregnant" is:

And word_abortion = No, I use a different word. The word(s) I use instead of "abortion" is:

srh_1_a1_bc1_preg1 From the below list of reproductive health issues facing people who share your sexual orientation and/or gender identity, please select the **three** that feel most important to you.

- ☐ Access to routine reproductive screenings and preventive care (e.g., screening for sexually transmitted infections, pap smears) (1)
- ☐ Fertility preservation (such as egg freezing) (2)
- ☐ Gaps in healthcare provider knowledge (3)
- ☐ Trans and gender-expansive affirming ${word_abortion/ChoiceTextEntryValue/2} care (4)
- ☐ LGBQ+ affirming ${word_abortion/ChoiceTextEntryValue/2} care (5)
- ☐ Trans and gender-expansive affirming ${word_bc/ChoiceTextEntryValue/2} options (6)
- ☐ LGBQ+ affirming ${word_bc/ChoiceTextEntryValue/2} options (7)
- ☐ Trans and gender-expansive affirming ${word_preg/ChoiceTextEntryValue/2} care (8)
- ☐ LGBQ+ affirming ${word_preg/ChoiceTextEntryValue/2} care (9)
- ☐ Lack of affirming patient education materials (10)
- ☐ Lack of cultural competency from healthcare staff/providers (11)
- ☐ Lack of inclusive/affirming sexual education curricula for use in training health care professionals (12)
- ☐ Medically accurate counseling about ${word_preg/ChoiceTextEntryValue/2} risk (13)
- ☐ Transgender and gender expansive individuals’ knowledge of basic health & health care issues (14)
- ☐ LGBQ+ individuals’ knowledge of basic health & health care issues (15)
- ☐ Not listed (please specify): (16)

____

- ☐ ☐ None of these (17)

Display This Question:

If word_abortion = No, I use a different word. The word(s) I use instead of "abortion" is:

And word_bc = No, I use a different word. The word(s) I use instead of "birth control" is:

And word_preg = Yes, I use the word "pregnant".

Or If

word_abortion = No, I use a different word. The word(s) I use instead of "abortion" is:

And word_bc = No, I use a different word. The word(s) I use instead of "birth control" is:

And word_preg = Prefer not to say

srh_1_a1_bc1_preg0 From the below list of reproductive health issues facing people who share your sexual orientation and/or gender identity, please select the **three** that feel most important to you.

▢ Access to routine reproductive screenings and preventive care (e.g., screening for sexually transmitted infections, pap smears) (1)

▢ Gaps in healthcare provider knowledge (2)

▢ Trans and gender-expansive affirming ${word_abortion/ChoiceTextEntryValue/2} care (3)

▢ LGBQ+ ${word_abortion/ChoiceTextEntryValue/2} care (4)

▢ Trans and gender-expansive affirming ${word_bc/ChoiceTextEntryValue/2} options (5)

▢ LGBQ+ affirming ${word_bc/ChoiceTextEntryValue/2} options (6)

▢ Trans and gender-expansive affirming pregnancy care (7)

▢ LGBQ+ affirming pregnancy care (8)

▢ Fertility preservation (such as egg freezing) (9)

▢ Lack of affirming patient education materials (10)

▢ Lack of cultural competency from healthcare staff/providers (11)

▢ Lack of inclusive/affirming sexual education curricula for use in training health care professionals (12)

▢ Medically accurate counseling about pregnancy risk (13)

▢ Transgender and gender expansive individuals' knowledge of basic health & health care issues (14)

▢ LGBQ+ individuals' knowledge of basic health & health care issues (15)

▢ Not listed (please specify): (16) ____

▢ ▢ None of these (17)

Display This Question:

If word_abortion = No, I use a different word. The word(s) I use instead of "abortion" is:

And word_bc = Yes, I use the words "birth control".

And word_preg = Yes, I use the word "pregnant".

Or If

word_abortion = No, I use a different word. The word(s) I use instead of "abortion" is:

And word_bc = Prefer not to say

And word_preg = Yes, I use the word "pregnant".

Or If

word_abortion = No, I use a different word. The word(s) I use instead of "abortion" is:

And word_bc = Yes, I use the words "birth control".

And word_preg = Prefer not to say

Or If

word_abortion = No, I use a different word. The word(s) I use instead of "abortion" is:

And word_bc = Prefer not to say

And word_preg = Prefer not to say

srh_1_a1_bc0_preg0 From the below list of reproductive health issues facing people who share your sexual orientation and/or gender identity, please select the **three** that feel most important to you.

▢ Access to routine reproductive screenings and preventive care (e.g., screening for sexually transmitted infections, pap smears) (1)

▢ Fertility preservation (such as egg freezing) (2)

▢ Gaps in healthcare provider knowledge (3)

▢ Trans and gender-expansive affirming ${word_abortion/ChoiceTextEntryValue/2} care (4)

▢ LGBQ+ affirming ${word_abortion/ChoiceTextEntryValue/2} care (5)

▢ Trans and gender-expansive affirming birth control options (6)

▢ LGBQ+ affirming birth control options (7)

▢ Trans and gender-expansive affirming pregnancy care (8)

▢ LGBQ+ affirming pregnancy care (9)

▢ Lack of affirming patient education materials (10)

▢ Lack of cultural competency from healthcare staff/providers (11)

▢ Lack of inclusive/affirming sexual education curricula for use in training health care professionals (12)

▢ Medically accurate counseling about pregnancy risk (13)

▢ Transgender and gender expansive individuals' knowledge of basic health & health care issues (14)

▢ LGBQ+ individuals' knowledge of basic health & health care issues (15)

▢ Not listed (please specify): (16) ____

▢ ▢ None of these (17)

Display This Question:

If word_abortion = No, I use a different word. The word(s) I use instead of "abortion" is:

And word_bc = Yes, I use the words "birth control".

And word_preg = No, I use a different word. The word I use instead of "pregnant" is:

Or If

word_abortion = No, I use a different word. The word(s) I use instead of "abortion" is:

And word_bc = Prefer not to say

And word_preg = No, I use a different word. The word I use instead of "pregnant" is:

srh_1_a1_bc0_preg1 From the below list of reproductive health issues facing people who share your sexual orientation and/or gender identity, please select the **three** that feel most important to you.

- [ ] Access to routine reproductive screenings and preventive care (e.g., screening for sexually transmitted infections, pap smears) (1)
- [ ] Fertility preservation (such as egg freezing) (2)
- [ ] Gaps in healthcare provider knowledge (3)
- [ ] Trans and gender-expansive affirming ${word_abortion/ChoiceTextEntryValue/2} care (4)
- [ ] LGBQ+ affirming ${word_abortion/ChoiceTextEntryValue/2} care (5)
- [ ] Trans and gender-expansive affirming birth control options (6)
- [ ] LGBQ+ affirming birth control options (7)
- [ ] Trans and gender-expansive affirming ${word_preg/ChoiceTextEntryValue/2} care (8)
- [ ] LGBQ+ ${word_preg/ChoiceTextEntryValue/2} care (9)
- [ ] Lack of affirming patient education materials (10)
- [ ] Lack of cultural competency from healthcare staff/providers (11)
- [ ] Lack of inclusive/affirming sexual education curricula for use in training health care professionals (12)
- [ ] Medically accurate counseling about ${word_preg/ChoiceTextEntryValue/2} risk (13)
- [ ] Transgender and gender expansive individuals' knowledge of basic health & health care issues (14)
- [ ] LGBQ+ individuals' knowledge of basic health & health care issues (15)
- [ ] Not listed (please specify): (16) ____
- [ ] None of these (17)

Display This Question:

If word_abortion = Yes, I use the word "abortion".

And word_bc = Yes, I use the words "birth control".

And word_preg = No, I use a different word. The word I use instead of "pregnant" is:

Or If

word_abortion = Yes, I use the word "abortion".

And word_bc = Prefer not to say

And word_preg = No, I use a different word. The word I use instead of "pregnant" is:

Or If

word_abortion = Prefer not to say

And word_bc = Prefer not to say

And word_preg = No, I use a different word. The word I use instead of "pregnant" is:

Or If

word_abortion = Prefer not to say

And word_bc = Yes, I use the words "birth control".

And word_preg = No, I use a different word. The word I use instead of "pregnant" is:

srh_1_a0_bc0_preg1 From the below list of reproductive health issues facing people who share your sexual orientation and/or gender identity, please select the **three** that feel most important to you.

- ☐ Access to routine reproductive screenings and preventive care (e.g., screening for sexually transmitted infections, pap smears) (1)
- ☐ Fertility preservation (such as egg freezing) (2)
- ☐ Gaps in healthcare provider knowledge (3)
- ☐ Trans and gender-expansive affirming abortion care (4)
- ☐ LGBQ+ affirming abortion care (5)
- ☐ Trans and gender-expansive affirming birth control options (6)
- ☐ LGBQ+ affirming birth control options (7)
- ☐ Trans and gender-expansive affirming ${word_preg/ChoiceTextEntryValue/2} care (8)
- ☐ LGBQ+ affirming ${word_preg/ChoiceTextEntryValue/2} care (9)
- ☐ Lack of affirming patient education materials (10)
- ☐ Lack of cultural competency from healthcare staff/providers (11)
- ☐ Lack of inclusive/affirming sexual education curricula for use in training health care professionals (12)
- ☐ Medically accurate counseling about ${word_preg/ChoiceTextEntryValue/2} risk (13)
- ☐ Transgender and gender expansive individuals’ knowledge of basic health & health care issues (14)
- ☐ LGBQ+ individuals’ knowledge of basic health & health care issues (15)
- ☐ Not listed (please specify): (16) ____
- ☐ ☐ None of these (17)

Display This Question:

If word_uterus = Yes, I use the word "uterus".

And word_vagina = Yes, I use the word "vagina".

Or If

word_vagina = Yes, I use the word "vagina".

And word_uterus = Prefer not to say

Or If

word_uterus = Prefer not to say

And word_vagina = Prefer not to say

Or If

word_vagina = Prefer not to say

And word_uterus = Yes, I use the word "uterus".

srh_2_u0_v0 Have you ever been diagnosed by a health care provider with any of the following conditions? **Select all that apply.**

- ☐ Abnormal pap smear (1)
- ☐ Cancer of a reproductive organ (i.e., cervix, fallopian tube, ovary, vagina, vulva, or uterus) (2)
- ☐ Chlamydia (3)
- ☐ Endometriosis (4)
- ☐ Fibroids of the uterus (5)
- ☐ Gonorrhea (6)
- ☐ Herpes (7)
- ☐ Human Immunodeficiency Virus (HIV) (8)
- ☐ Human Papilloma Virus (HPV) (9)
- ☐ Polycystic ovarian syndrome (PCOS) or ovarian cysts (10)
- ☐ Recurrent vaginal infections, e.g. bacterial vaginosis or yeast infections (11)
- ☐ Syphillis (12)
- ☐ Not listed (please specify): (13) ____
- ☐ ☐ None of these (15)
- ☐ ☐ I don't know (14)

Display This Question:

If word_uterus = No, I use a different word. The word I use instead of "uterus" is:

And word_vagina = Yes, I use the word "vagina".

Or If

word_vagina = Prefer not to say

And word_uterus = No, I use a different word. The word I use instead of "uterus" is:

srh_2_u1_v0 Have you ever been diagnosed by a health care provider with any of the following conditions? Select all that apply.

- ☐ Abnormal pap smear (1)
- ☐ Cancer of a reproductive organ (i.e., cervix, fallopian tube, ovary, vagina, vulva, or ${word_uterus/ChoiceTextEntryValue/2}) (2)
- ☐ Chlamydia (3)
- ☐ Endometriosis (4)
- ☐ Fibroids of the ${word_uterus/ChoiceTextEntryValue/2} (5)
- ☐ Gonorrhea (6)
- ☐ Herpes (7)
- ☐ Human Immunodeficiency Virus (HIV) (8)
- ☐ Human Papilloma Virus (HPV) (9)
- ☐ Polycystic ovarian syndrome (PCOS) or ovarian cysts (10)
- ☐ Recurrent vaginal infections, e.g. bacterial vaginosis or yeast infections (11)
- ☐ Syphillis (12)
- ☐ Not listed (please specify): (13) ____
- ☐ ☐ None of these (15)
- ☐ ☐ I don't know (14)

Display This Question:

If word_uterus = No, I use a different word. The word I use instead of "uterus" is:

And word_vagina = No, I use a different word. The word I use instead of "vagina" is:

srh_2_u1_v1 Have you ever been diagnosed by a health care provider with any of the following conditions? Select all that apply.

☐ Abnormal pap smear (1)

☐ Cancer of a reproductive organ (i.e., cervix, fallopian tube, ovary, ${word_vagina/ChoiceTextEntryValue/2}, vulva, or ${word_uterus/ChoiceTextEntryValue/2}) (2)

☐ Chlamydia (3)

☐ Endometriosis (4)

☐ Fibroids of the ${word_uterus/ChoiceTextEntryValue/2} (5)

☐ Gonorrhea (6)

☐ Herpes (7)

☐ Human Immunodeficiency Virus (HIV) (8)

☐ Human Papilloma Virus (HPV) (9)

☐ Polycystic ovarian syndrom (PCOS) or ovarian cysts (10)

☐ Recurrent ${word_vagina/ChoiceTextEntryValue/2} infections, e.g. bacterial vaginosis or yeast infections (11)

☐ Syphillis (12)

☐ Not listed (please specify): (13) ____

☐ ☐ None of these (15)

☐ ☐ I don't know (14)

Display This Question:

If word_uterus = Yes, I use the word "uterus".

And word_vagina = No, I use a different word. The word I use instead of "vagina" is:

Or If

word_vagina = No, I use a different word. The word I use instead of "vagina" is:

And word_uterus = Prefer not to say

srh_2_u0_v1 Have you ever been diagnosed with any of the following conditions? Select all that apply.

- [ ] Abnormal pap smear (1)
- [ ] Cancer of a reproductive organ (i.e., cervix, fallopian tube, ovary, ${word_vagina/ChoiceTextEntryValue/2}, vulva, or uterus) (2)
- [ ] Chlamydia (3)
- [ ] Endometriosis (4)
- [ ] Fibroids of the uterus (5)
- [ ] Gonorrhea (6)
- [ ] Herpes (7)
- [ ] Human Immunodeficiency Virus (HIV) (8)
- [ ] Human Papilloma Virus (HPV) (9)
- [ ] Polycystic ovarian syndrom (PCOS) or ovarian cysts (10)
- [ ] Recurrent ${word_vagina/ChoiceTextEntryValue/2} infections, e.g. bacterial vaginosis or yeast infections (11)
- [ ] Syphillis (12)
- [ ] Not listed (please specify): (13) ____
- [ ] ☐ None of these (15)
- [ ] ☐ I don't know (14)

Display This Question:

If srh_2_u1_v1 != Human Immunodeficiency Virus (HIV)

Or srh_2_u0_v1 != Human Immunodeficiency Virus (HIV)

Or srh_2_u0_v0 != Human Immunodeficiency Virus (HIV)

Or srh_2_u1_v0 != Human Immunodeficiency Virus (HIV)

srh_prep Are you currently using PrEP? (PrEP is short for "pre-exposure prophylaxis", a once-daily pill that can help you stay HIV-negative, sold under the brand name Truvada.)

- Yes, I am currently taking PrEP (1)
- I am not currently taking PrEP, but have used it in the past (2)
- No, I have never taken PrEP (0)

Page Break

Display This Question:

If word_bc = Yes, I use the words "birth control".

And word_preg = Yes, I use the word "pregnant".

Or If

word_bc = Prefer not to say

And word_preg = Yes, I use the word "pregnant".

Or If

word_preg = Prefer not to say

And word_bc = Prefer not to say

Or If

word_bc = Yes, I use the words "birth control".

And word_preg = Prefer not to say

bc_9_bc0_preg0 Has a provider ever discussed birth control methods with you for the purposes of pregnancy prevention? **Select all that apply.**

- Yes, I brought it up (1)
- Yes, my provider brought it up (2)
- No (0)
- I don't remember (3)

Display This Question:

If word_bc = No, I use a different word. The word(s) I use instead of "birth control" is:

And word_preg = Yes, I use the word "pregnant".

Or If

word_preg = Prefer not to say

And word_bc = No, I use a different word. The word(s) I use instead of "birth control" is:

bc_9_bc1_preg0 Has a provider ever discussed ${word_bc/ChoiceTextEntryValue/2} methods with you for the purposes of pregnancy prevention? **Select all that apply.**

- Yes, I brought it up (1)
- Yes, my provider brought it up (2)
- No (0)
- I don't remember (3)

Display This Question:

If word_bc = Prefer not to say

And word_preg = No, I use a different word. The word I use instead of "pregnant" is:

Or If

word_preg = No, I use a different word. The word I use instead of "pregnant" is:

And word_bc = Yes, I use the words "birth control".

bc_9_bc0_preg1 Has a provider ever discussed birth control methods with you for the purposes of ${word_preg/ChoiceTextEntryValue/2} prevention? **Select all that apply.**

- Yes, I brought it up (1)
- Yes, my provider brought it up (2)
- No (0)
- I don't remember (3)

Display This Question:

If word_bc = No, I use a different word. The word(s) I use instead of "birth control" is:

And word_preg = No, I use a different word. The word I use instead of "pregnant" is:

bc_9_bc1_preg1 Has a provider ever discussed ${word_bc/ChoiceTextEntryValue/2} methods with you for the purposes of ${word_preg/ChoiceTextEntryValue/2} prevention? **Select all that apply.**

- ☐ Yes, I brought it up (1)
- ☐ Yes, my provider brought it up (2)
- ☐ ☐ No (0)
- ☐ ☐ I don't remember (3)

Display This Question:

If bc_9_bc0_preg0 = Yes, I brought it up

Or bc_9_bc0_preg0 = Yes, my provider brought it up

Or bc_9_bc0_preg1 = Yes, I brought it up

Or bc_9_bc0_preg1 = Yes, my provider brought it up

And If

ga_1 = Hormone Treatment / Hormone Replacement Therapy (HRT)

Or ga_1 = Gender affirming genital surger(ies) / reconstructive surger(ies) or bottom surger(ies)

Or ga_1 = Medications to stop or delay the onset of puberty (often called "puberty blockers", usually used by youth)

X→

bc_10_bc0 When did you and your provider discuss these birth control methods? **Select all that apply.**

- ☐ Before I began to pursue gender-affirming hormone therapy and/or surgery (1)
- ☐ After I began to pursue gender-affirming hormone therapy and/or surgery (2)
- ☐ ☐ I don't remember (3)

Display This Question:

If bc_9_bc1_preg0 = Yes, I brought it up

Or bc_9_bc1_preg0 = Yes, my provider brought it up

Or bc_9_bc1_preg1 = Yes, I brought it up

Or bc_9_bc1_preg1 = Yes, my provider brought it up

And If

ga_1 = Gender affirming genital surger(ies) / reconstructive surger(ies) or bottom surger(ies)

Or ga_1 = Hormone Treatment / Hormone Replacement Therapy (HRT)

Or ga_1 = Medications to stop or delay the onset of puberty (often called "puberty blockers", usually used by youth)

bc_10_bc1 When did you and your provider discuss these ${word_bc/ChoiceTextEntryValue/2} methods? **Select all that apply.**

- ☐ Before I began to pursue gender-affirming hormone therapy and/or surgery (1)
- ☐ After I began to pursue gender-affirming hormone therapy and/or surgery (2)
- ☐ ☐ I don't remember (3)

Display This Question:

If bc_9_bc0_preg0 = Yes, I brought it up

Or bc_9_bc0_preg0 = Yes, my provider brought it up

bc_11_bc0 How comfortable did you feel asking your provider all of the questions you had about birth control?

- ○ Very comfortable (1)
- ○ Somewhat comfortable (2)
- ○ A little comfortable (3)
- ○ Not at all comfortable (4)
- ○ I did not have any questions about birth control (5)

Display This Question:

If bc_9_bc1_preg0 = Yes, I brought it up

Or bc_9_bc1_preg0 = Yes, my provider brought it up

bc_11_bc1 How comfortable did you feel asking your provider all of the questions you had about ${word_bc/ChoiceTextEntryValue/2}?

- Very comfortable (1)
- Somewhat comfortable (2)
- A little comfortable (3)
- Not at all comfortable (4)
- I did not have any questions about ${word_bc/ChoiceTextEntryValue/2} (5)

Display This Question:

If word_bc = Yes, I use the words "birth control".

Or word_bc = Prefer not to say

bc_12_bc0 Has a provider ever recommended specific type(s) of birth control for you based on your body size?

- Yes (1)
- No (0)
- I don't know (2)

Display This Question:

If word_bc = No, I use a different word. The word(s) I use instead of "birth control" is:

bc_12_bc1 Has a provider ever recommended specific type(s) of ${word_bc/ChoiceTextEntryValue/2} for you based on your body size?

○ Yes (1)

○ No (0)

○ I don't know (2)

Display This Question:

If word_bc = Yes, I use the words "birth control".

Or word_bc = Prefer not to say

bc_13_bc0 Some birth control methods work differently depending on a person's body size. In the next two questions, we will ask you about your height and weight so that we can better understand your experience with birth control.

What is your height?

Please enter a number in each box, between 3 - 7 feet and 0 - 12 inches.

○ Feet (1) ____

○ Inches (2) ____

Display This Question:

If word_bc = No, I use a different word. The word(s) I use instead of "birth control" is:

bc_13_bc1 Some ${word_bc/ChoiceTextEntryValue/2} methods work differently depending on a person's body size. In the next two questions, we will ask you about your height and weight so that we can better understand your experience with ${word_bc/ChoiceTextEntryValue/2}.

What is your height?

Please enter a number in each box, between 3 - 7 feet and 0 - 12 inches.

- ○ Feet (1) ____
- ○ Inches (2) ____

* * *

*

bc_14 What is your weight in pounds?

____

* * *

Page Break

Display This Question:

If bc_1_bc0_preg0 = Yes

Or bc_1_bc0_preg1 = Yes

bc_15_bc0 What factors have made it **EASIER** for you to access prescription birth control care? Select all that apply.

- ▢ Affirming and inclusive language used by birth control providers (1)
- ▢ Gender-neutral patient materials and intake forms (2)
- ▢ My insurance covers birth control (3)
- ▢ Sliding scale fees make the cost affordable (4)
- ▢ There is a birth control provider near me (5)
- ▢ Not listed (please specify): (6) ____
- ▢ ⊗None of these (7)
- ▢ ⊗I have not tried to access prescription birth control (8)

Skip To: bc_17 If bc_15_bc0 = I have not tried to access prescription birth control

Display This Question:

If bc_1_bc1_preg0 = Yes

Or bc_1_bc1_preg1 = Yes

bc_15_bc1 What factors have made it **EASIER** for you to access prescription ${word_bc/ChoiceTextEntryValue/2} care? Select all that apply.

- ▢ Affirming and inclusive language used by ${word_bc/ChoiceTextEntryValue/2} providers (1)
- ▢ Gender-neutral patient materials and intake forms (2)
- ▢ My insurance covers ${word_bc/ChoiceTextEntryValue/2} (3)
- ▢ Sliding scale fees make the cost affordable (4)
- ▢ There is a ${word_bc/ChoiceTextEntryValue/2} provider near me (5)
- ▢ Not listed (please specify): (6) ____
- ▢ ▢ None of these (7)
- ▢ ▢ I have not tried to access prescription ${word_bc/ChoiceTextEntryValue/2} (8)

Skip To: bc_17 If bc_15_bc1 = I have not tried to access prescription ${q://QID30/ChoiceTextEntryValue/2}

Display This Question:

If bc_1_bc0_preg0 = Yes

Or bc_1_bc0_preg1 = Yes

bc_16_bc0 Is there anything **YOU WOULD RECOMMEND** to improve the birth control care that you received? Select all that apply.

- ☐ Ability to request birth control through an online portal (1)
- ☐ Birth control prescription delivery by mail is made available (2)
- ☐ Clinic/office is accessible by public transportation (3)
- ☐ Closer clinic/office location (4)
- ☐ Gender-neutral language used by staff (5)
- ☐ Intake forms that are gender-neutral or gender-affirming (6)
- ☐ More support from my provider (7)
- ☐ More support from the clinic staff (8)
- ☐ More privacy outside of the clinic (less obvious signage, more discrete entrance, etc.) (9)
- ☐ More privacy within the clinic (10)
- ☐ Not listed (please specify): (11) ____
- ☐ ☐ None of these (12)

Display This Question:

If bc_1_bc1_preg1 = Yes

And bc_1_bc1_preg0 = Yes

bc_16_bc1 Is there anything **YOU WOULD RECOMMEND** to improve the ${word_bc/ChoiceTextEntryValue/2} care that you received? Select all that apply.

- ☐ Ability to request ${word_bc/ChoiceTextEntryValue/2} through an online portal (1)
- ☐ ${word_bc/ChoiceTextEntryValue/2} prescription delivery by mail is made available (2)
- ☐ Closer clinic/office location (3)
- ☐ Clinic/office is accessible by public transportation (4)
- ☐ Gender-neutral language used by staff (5)
- ☐ Intake forms that are gender-neutral or gender-affirming (6)
- ☐ More support from my provider (7)
- ☐ More support from the clinic staff (8)
- ☐ More privacy outside of the clinic (less obvious signage, more discrete entrance, etc.) (9)
- ☐ More privacy within the clinic (10)
- ☐ Not listed (please specify): (11) ____
- ☐ ☐ None of these (12)

Page Break

Display This Question:

If bc_15_bc0 = I have not tried to access prescription birth control

Or bc_15_bc1 = I have not tried to access prescription ${q://QID30/ChoiceTextEntryValue/2}

bc_17 What are the reasons why you have not used birth control? **Select all that apply.**

Due to the limitations in electronic survey design and the many options below, we are unable to use your preferred words for this question and will instead use medical terms.

- ☐ Birth control is not available near me. (1)
- ☐ I am afraid of discrimination I might face in asking for birth control. (2)
- ☐ I am concerned about the side effects of birth control. (3)
- ☐ I do not want to use estrogen or feminizing hormones/am concerned birth control will interfere with my gender affirmation process. (4)
- ☐ I am not capable of getting pregnant. (5)
- ☐ I am not worried about sexually transmitted infections (STIs). (6)
- ☐ I am trying to/wouldn't mind getting pregnant. (7)
- ☐ I cannot afford birth control. (8)
- ☐ I do not engage in penis-in-vagina sex. (9)
- ☐ I do not engage in sex with people who produce sperm. (22)
- ☐ I do not want others to know I am using birth control. (10)
- ☐ I don't have a health care provider. (11)
- ☐ I don't know what my birth control options are. (12)
- ☐ I don't have health insurance. (13)
- ☐ My insurance does not cover birth control. (23)
- ☐ My partner(s) does/do not want us to use birth control during sex. (14)
- ☐ Use of birth control makes me feel disconnected from my gender identity. (15)

☐ I **do not** have any symptoms such as chest tenderness, bloating, acne, pain from cramping, heavy bleeding (sometimes referred to as pre-menstrual syndrome or PMS) that birth control is sometimes used to help treat. (16)

☐ I **do** have any symptoms such as chest tenderness, bloating, acne, pain from cramping, heavy bleeding (sometimes referred to as pre-menstrual syndrome or PMS) <u>but I did not know or was not offered birth control to treat those</u>. (17)

☐ I **do not** have any medical conditions that would benefit from using birth control (acne, chronic pelvic pain, facial hair growth). (18)

☐ I **do** have medical conditions that would benefit from using birth control (acne, chronic pelvic pain, facial hair growth) but I did not know or was not offered birth control to treat those. (19)

☐ I did not want to alter or stop my period. (20)

☐ Other (please describe): (21)
____

Page Break

Display This Question:

If word_bc = Yes, I use the words "birth control".

Or word_bc = Prefer not to say

bc_18_bc0 Have any of the below difficulties ever made it **HARDER** for you to get birth control? Select all that apply.

- [ ] Cost of birth control (1)
- [ ] Cost of health care visit for birth control (2)
- [ ] Difficulty of finding an affirming and knowledgeable health care provider (3)
- [ ] The time required to get birth control (4)
- [ ] The travel required to get birth control (5)
- [ ] Not listed (please specify): (6) ____
- [ ] None of these (7)

Display This Question:

If word_bc = No, I use a different word. The word(s) I use instead of "birth control" is:

bc_18_bc1 Have any of the below difficulties ever made it **HARDER** for you to get ${word_bc/ChoiceTextEntryValue/2}? Select all that apply.

- ☐ Cost of ${word_bc/ChoiceTextEntryValue/2} (1)
- ☐ Cost of health care visit for ${word_bc/ChoiceTextEntryValue/2} (2)
- ☐ Difficulty of finding an affirming and knowledgeable health care provider (3)
- ☐ The time required to get ${word_bc/ChoiceTextEntryValue/2} (4)
- ☐ The travel required to get ${word_bc/ChoiceTextEntryValue/2} (5)
- ☐ Not listed (please specify): (6) ____
- ☐ ☐ None of these (7)

Display This Question:

If word_bc = Yes, I use the words "birth control".

Or word_bc = Prefer not to say

And If

legal = My health insurance

X→

bc_19_bc0 Has your legal gender on your health insurance ever prevented you from having birth control covered by your insurance?

- ○ Yes (1)
- ○ No (0)
- ○ I have not tried to use my health insurance to cover this service (3)
- ○ My insurance does not cover this service (4)
- ○ I don't know (2)

Display This Question:

If word_bc = No, I use a different word. The word(s) I use instead of "birth control" is:

And legal = My health insurance

bc_19_bc1 Has your "legal" gender on your health insurance ever prevented you from having ${word_bc/ChoiceTextEntryValue/2} covered by your insurance?

- Yes (1)
- No (0)
- I have not tried to use my health insurance to cover this service (3)
- My insurance does not cover this service (4)
- I don't know (2)

Display This Question:

If word_bc = Yes, I use the words "birth control".

Or word_bc = Prefer not to say

bc_20_bc0 Does whether or not a birth control method releases estrogen in your body matter to you?

- Yes (1)
- No (0)
- I don't know (2)

Display This Question:

If word_bc = No, I use a different word. The word(s) I use instead of "birth control" is:

bc_20_bc1 Does whether or not a ${word_bc/ChoiceTextEntryValue/2} method releases estrogen in your body matter to you?

◯ Yes (1)

◯ No (0)

◯ I don't know (2)

Display This Question:

If bc_20_bc0 = Yes

Or bc_20_bc1 = Yes

bc_21 Why does it matter to you if estrogen is released in your body? Select all that apply.

▢ I do not want to use estrogen because it is a "female" hormone (1)

▢ I do not want to use estrogen because of its "feminizing" effects (2)

▢ Another reason that is not listed (please describe): (3) ____

▢ ▢ I don't know (4)

Display This Question:

If word_bc = Yes, I use the words "birth control".

And word_uterus = Yes, I use the word "uterus".

And ga_surg_3_u0 != Have had it

Or If

word_bc = Yes, I use the words "birth control".

And word_uterus = Prefer not to say

And ga_surg_3_u0 != Have had it

Or If

word_bc = Prefer not to say

And word_uterus = Yes, I use the word "uterus".

And ga_surg_3_u0 != Have had it

Or If

word_uterus = Prefer not to say

And word_bc = Prefer not to say

And ga_surg_3_u0 != Have had it

bc_22_bc0_u0 Would you use a birth control pill that only had progestin that you could buy over the counter (without a prescription)?

Progestin-only pills (also called the "mini-pill") are a pill taken daily by the mouth that do not contain estrogen, and instead release only one type of hormone, progestin. This hormone works by stopping the ovaries from releasing eggs and by thickening the cervical mucus and thinning the lining of the uterus. Progestin is not a feminizing hormone and does not interfere with gender affirmation/HRT/medical transition.

- Yes (1)
- No (0)
- I don't know (2)

Display This Question:

If word_uterus = Yes, I use the word "uterus".

And word_bc = No, I use a different word. The word(s) I use instead of "birth control" is:

And ga_surg_3_u0 != Have had it

Or If

word_bc = No, I use a different word. The word(s) I use instead of "birth control" is:

And word_uterus = Prefer not to say

And ga_surg_3_u0 != Have had it

X→

bc_22_bc1_u0 Would you use a ${word_bc/ChoiceTextEntryValue/2} pill that only had progestin that you could buy over the counter (without a prescription)?

Progestin-only pills (also called the "mini-pill") are a pill taken daily by the mouth that do not contain estrogen, and instead release only one type of hormone, progestin. This hormone works by stopping the ovaries from releasing eggs and by thickening the cervical mucus and thinning the lining of the uterus. Progestin is not a feminizing hormone and does not interfere with gender affirmation/HRT/medical transition.

- Yes (1)
- No (0)
- I don't know (2)

Display This Question:

If word_uterus = No, I use a different word. The word I use instead of "uterus" is:

And word_bc = Yes, I use the words "birth control".

And ga_surg_3_u1 != Have had it

Or If

word_bc = Prefer not to say

And word_uterus = No, I use a different word. The word I use instead of "uterus" is:

And ga_surg_3_u1 != Have had it

X→

bc_22_bc0_u1 Would you use a birth control pill that only had progestin that you could buy over the counter (without a prescription)?

Progestin-only pills (also called the "mini-pill") are a pill taken daily by the mouth that do not

contain estrogen, and instead release only one type of hormone, progestin. This hormone works by stopping the ovaries from releasing eggs and by thickening the cervical mucus and thinning the lining of the ${word_uterus/ChoiceTextEntryValue/2}. Progestin is not a feminizing hormone and does not interfere with gender affirmation/HRT/medical transition.

○ Yes (1)

○ No (0)

○ I don't know (2)

Display This Question:

If word_uterus = No, I use a different word. The word I use instead of "uterus" is:

And word_bc = No, I use a different word. The word(s) I use instead of "birth control" is:

And ga_surg_3_u1 != Have had it

X→

bc_22_bc1_u1 Would you use a ${word_bc/ChoiceTextEntryValue/2} pill that only had progestin that you could buy over the counter (without a prescription)?

Progestin-only pills (also called the "mini-pill") are a pill taken daily by the mouth that do not contain estrogen, and instead release only one type of hormone, progestin. This hormone works by stopping the ovaries from releasing eggs and by thickening the cervical mucus and thinning the lining of the ${word_uterus/ChoiceTextEntryValue/2}. Progestin is not a feminizing hormone and does not interfere with gender affirmation/HRT/medical transition.

○ Yes (1)

○ No (0)

○ I don't know (2)

Page Break

Display This Question:

If word_abortion = Yes, I use the word "abortion".

Or word_abortion = Prefer not to say

ab_1_a0 Whether or not you have had an abortion(s), has a provider ever discussed abortion with you? **Select all that apply.**

- Yes, I brought it up (1)
- Yes, my provider brought it up (2)
- No (0)
- I don't remember (3)

Skip To: ab_4_a0 If ab_1_a0 = No

Skip To: ab_4_a0 If ab_1_a0 = I don't remember

Display This Question:

If word_abortion = No, I use a different word. The word(s) I use instead of "abortion" is:

ab_1_a1 Whether or not you have had an ${word_abortion/ChoiceTextEntryValue/2}(s), has a provider ever discussed ${word_abortion/ChoiceTextEntryValue/2} with you? **Select all that apply.**

- Yes, I brought it up (1)
- Yes, my provider brought it up (2)
- No (0)
- I don't remember (3)

Skip To: ab_4_a1 If ab_1_a1 = No

Skip To: ab_4_a1 If ab_1_a1 = I don't remember

Display This Question:

If ab_1_a0 = Yes, I brought it up

Or ab_1_a0 = Yes, my provider brought it up

And If

ga_1 = Gender affirming genital surger(ies) / reconstructive surger(ies) or bottom surger(ies)

Or ga_1 = Hormone Treatment / Hormone Replacement Therapy (HRT)

Or ga_1 = Medications to stop or delay the onset of puberty (often called "puberty blockers", usually used by youth)

X→

ab_2_a0 When did you and your provider discuss abortion? **Select all that apply.**

- ▢ Before I began to pursue gender-affirming hormone therapy and/or surgery (0)
- ▢ After I began to pursue gender-affirming hormone therapy and/or surgery (1)
- ▢ ▢ I don't remember (2)

Display This Question:

If ab_1_a1 = Yes, I brought it up

Or ab_1_a1 = Yes, my provider brought it up

And If

ga_1 = Gender affirming genital surger(ies) / reconstructive surger(ies) or bottom surger(ies)

Or ga_1 = Hormone Treatment / Hormone Replacement Therapy (HRT)

Or ga_1 = Medications to stop or delay the onset of puberty (often called "puberty blockers", usually used by youth)

X→

ab_2_a1 When did you and your provider discuss ${word_abortion/ChoiceTextEntryValue/2}? **Select all that apply.**

- ▢ Before I began to pursue gender-affirming hormone therapy and/or surgery (0)
- ▢ After I began to pursue gender-affirming hormone therapy and/or surgery (1)
- ▢ ▢ I don't remember (2)

Display This Question:

If ab_1_a0 = Yes, I brought it up

Or ab_1_a0 = Yes, my provider brought it up

ab_3_a0 How comfortable were you asking your provider all of the questions you had about abortion care?

- Very comfortable (1)
- Somewhat comfortable (2)
- A little comfortable (3)
- Not at all comfortable (4)
- I don't remember (5)

Display This Question:

If ab_1_a1 = Yes, I brought it up

Or ab_1_a1 = Yes, my provider brought it up

ab_3_a1 How comfortable were you asking your provider all of the questions you had about ${word_abortion/ChoiceTextEntryValue/2} care?

- Very comfortable (1)
- Somewhat comfortable (2)
- A little comfortable (3)
- Not at all comfortable (4)
- I don't remember (5)

Display This Question:

If word_abortion = Yes, I use the word "abortion".

Or word_abortion = Prefer not to say

And If

ga_surg_3_u0 != Have had it

And ga_surg_3_u1 != Have had it

ab_4_a0 If you needed an abortion now, what type of abortion would you prefer?

- Medication abortion (abortion with pills) (1)
- Surgical abortion (a short procedure performed by a health care provider) (2)
- Not listed (please specify): (3) ____
- I don't know (4)

Display This Question:

If word_abortion = No, I use a different word. The word(s) I use instead of "abortion" is:

And If

ga_surg_3_u0 != Have had it

And ga_surg_3_u1 != Have had it

ab_4_a1 If you needed an ${word_abortion/ChoiceTextEntryValue/2} now, what type of ${word_abortion/ChoiceTextEntryValue/2} would you prefer?

- Medication ${word_abortion/ChoiceTextEntryValue/2} (${word_abortion/ChoiceTextEntryValue/2} with pills) (1)
- Surgical ${word_abortion/ChoiceTextEntryValue/2} (a short procedure performed by a health care provider) (2)
- Not listed (please specify): (3) ____
- I don't know (4)

Display This Question:

If ab_4_a0 = Medication abortion (abortion with pills)

Or ab_4_a0 = Surgical abortion (a short procedure performed by a health care provider)

Or ab_4_a0 = Not listed (please specify):

ab_5_a0 What are the main reasons that this is your preferred method of abortion? **Please select up to three responses.**

- ☐ I feel most comfortable with the type and number of medical staff present for this option (1)
- ☐ I have had this type of abortion before and know what to expect (2)
- ☐ I know somebody who has had this type of abortion (3)
- ☐ This is the only method available in my area (4)
- ☐ This method costs the least amount of money (5)
- ☐ This method does not require anesthesia (6)
- ☐ This method does require anesthesia (7)
- ☐ This method feels the most private (8)
- ☐ This method is easier to schedule (9)
- ☐ This method is the least invasive (10)
- ☐ This method is the least painful (11)
- ☐ This method is the only method with which I am familiar (12)
- ☐ This method requires the fewest visits (13)
- ☐ This method would take the least amount of time (is fastest) (14)
- ☐ Not listed (please specify): (15) ____
- ☐ ☐ None of these (16)

Display This Question:

If ab_4_a1 = Medication ${q://QID31/ChoiceTextEntryValue/2} (${q://QID31/ChoiceTextEntryValue/2} with pills)

Or ab_4_a1 = Surgical ${q://QID31/ChoiceTextEntryValue/2} (a short procedure performed by a health care provider)

Or ab_4_a1 = Not listed (please specify):

ab_5_a1 What are the main reasons that this is your preferred method of ${word_abortion/ChoiceTextEntryValue/2}? **Please select up to three responses.**

- I feel most comfortable with the type and number of medical staff present for this option (1)
- I have had this type of ${word_abortion/ChoiceTextEntryValue/2} before and know what to expect (2)
- I know somebody who has had this type of ${word_abortion/ChoiceTextEntryValue/2} (3)
- This method is the only method with which I am familiar (4)
- This is the only method available in my area (5)
- This method costs the least amount of money (6)
- This method feels the most private (7)
- This method is easier to schedule (8)
- This method is the least invasive (9)
- This method is the least painful (10)
- This method does not require anesthesia (11)
- This method does require anesthesia (12)
- This method requires the fewest visits (13)
- This method would take the least amount of time (is fastest) (14)
- Not listed (please specify): (15) ____
- None of these (16)

Page Break

Display This Question:

If Loop any: phx_3_a0_preg0 = Abortion

Or Loop any: phx_3_a0_preg1 = Abortion

ab_6_a0 What types of abortions have you had? Select all that apply.

- ▢ Medication abortion (abortion with pills) (1)
- ▢ Surgical abortion (a short procedure performed by a health care provider) (2)
- ▢ Not listed (please specify): (3) ____

Display This Question:

If Loop any: phx_3_a1_preg1 = ${q://QID31/ChoiceTextEntryValue/2}

Or Loop any: phx_3_a1_preg0 = ${q://QID31/ChoiceTextEntryValue/2}

ab_6_a1 What types of ${word_abortion/ChoiceTextEntryValue/2}s have you had? Select all that apply.

- ▢ Medication ${word_abortion/ChoiceTextEntryValue/2} (${word_abortion/ChoiceTextEntryValue/2} with pills) (1)
- ▢ Surgical ${word_abortion/ChoiceTextEntryValue/2} (a short procedure performed by a health care provider) (2)
- ▢ Not listed (please specify): (3) ____

Display This Question:

If Loop any: phx_3_a0_preg0 = Abortion

Or Loop any: phx_3_a0_preg1 = Abortion

ab_7_a0 For your most recent abortion, what type of abortion procedure did you have?

◯ Medication abortion (abortion with pills) (1)

◯ Surgical abortion (a short procedure performed by a health care provider) (2)

◯ Not listed (please specify): (3) ____

Display This Question:

If Loop any: phx_3_a1_preg1 = ${q://QID31/ChoiceTextEntryValue/2}

Or Loop any: phx_3_a1_preg0 = ${q://QID31/ChoiceTextEntryValue/2}

ab_7_a1 For your most recent ${word_abortion/ChoiceTextEntryValue/2}, what type of ${word_abortion/ChoiceTextEntryValue/2} procedure did you have?

◯ Medication ${word_abortion/ChoiceTextEntryValue/2} (${word_abortion/ChoiceTextEntryValue/2} with pills) (1)

◯ Surgical ${word_abortion/ChoiceTextEntryValue/2} (a short procedure performed by a health care provider) (2)

◯ Not listed (please specify): (3) ____

Display This Question:

If Loop any: phx_3_a0_preg0 = Abortion

Or Loop any: phx_3_a0_preg1 = Abortion

JS X→

ab_8_a0 In what month and year did you have your most recent abortion? (It is ok if you can't remember the exact date, please just do your best to estimate.)

	Month	Year
Please Select: (1)	▼ January (1 ... December (12)	▼ 1965 (1 ... 2019 (55)

Display This Question:

If Loop any: phx_3_a1_preg1 = ${q://QID31/ChoiceTextEntryValue/2}

Or Loop any: phx_3_a1_preg0 = ${q://QID31/ChoiceTextEntryValue/2}

JS X→

ab_8_a1 In what month and year did you have your most recent ${word_abortion/ChoiceTextEntryValue/2}? (It is ok if you can't remember the exact date, please just do your best to estimate.)

	Month	Year
Please Select: (1)	▼ January (1 ... December (12)	▼ 1965 (1 ... 2019 (55)

Display This Question:

If word_abortion = Yes, I use the word "abortion".

And word_period = Yes, I use the word "period".

And word_preg = Prefer not to say

And Loop any: phx_3_a0_preg0 = Abortion

Or If

word_abortion = Yes, I use the word "abortion".

And word_period = Prefer not to say

And word_preg = Prefer not to say

And Loop any: phx_3_a0_preg0 = Abortion

Or If

word_abortion = Prefer not to say

And word_period = Prefer not to say

And word_preg = Prefer not to say

And Loop any: phx_3_a0_preg0 = Abortion

Or If

word_abortion = Prefer not to say

And word_period = Yes, I use the word "period".

And word_preg = Yes, I use the word "pregnant".

And Loop any: phx_3_a0_preg0 = Abortion

Or If

word_abortion = Prefer not to say

And word_period = Prefer not to say

And word_preg = Yes, I use the word "pregnant".

And Loop any: phx_3_a0_preg0 = Abortion

Or If

word_abortion = Prefer not to say

And word_period = Yes, I use the word "period".

And word_preg = Prefer not to say

And Loop any: phx_3_a0_preg0 = Abortion

Or If

word_abortion = Yes, I use the word "abortion".

And word_period = Prefer not to say

And word_preg = Prefer not to say

And Loop any: phx_3_a0_preg0 = Abortion

Or If

word_abortion = Yes, I use the word "abortion".

And word_period = Yes, I use the word "period".

And word_preg = Yes, I use the word "pregnant".

And Loop any: phx_3_a0_preg0 = Abortion

ab_9_a0_per0_preg0 Approximately how many weeks pregnant were you at the time of this most recent abortion? (measured from the date of your last period)

○ Number of weeks: (1) ____

○ I don't know (2)

Display This Question:

If word_abortion = Yes, I use the word "abortion".

And word_period = Prefer not to say

And word_preg = No, I use a different word. The word I use instead of "pregnant" is:

And Loop any: phx_3_a0_preg1 = Abortion

Or If

word_abortion = Prefer not to say

And word_period = Prefer not to say

And word_preg = No, I use a different word. The word I use instead of "pregnant" is:

And Loop any: phx_3_a0_preg1 = Abortion

Or If

word_abortion = Prefer not to say

And word_period = Yes, I use the word "period".

And word_preg = No, I use a different word. The word I use instead of "pregnant" is:

And Loop any: phx_3_a0_preg1 = Abortion

Or If

word_abortion = Yes, I use the word "abortion".

And word_period = Yes, I use the word "period".

And word_preg = No, I use a different word. The word I use instead of "pregnant" is:

And Loop any: phx_3_a0_preg1 = Abortion

ab_9_a0_per0_preg1 Approximately how many weeks ${word_preg/ChoiceTextEntryValue/2} were you at the time of this most recent abortion? (measured from the date of your last period)

○ Number of weeks: (1) ____

○ I don't know (2)

Display This Question:

If word_preg = No, I use a different word. The word I use instead of "pregnant" is:

And word_abortion = No, I use a different word. The word(s) I use instead of "abortion" is:

And word_period = Yes, I use the word "period".

And Loop any: phx_3_a1_preg1 = ${q://QID31/ChoiceTextEntryValue/2}

Or If

word_abortion = No, I use a different word. The word(s) I use instead of "abortion" is:

And word_period = Prefer not to say

And word_preg = No, I use a different word. The word I use instead of "pregnant" is:

And Loop any: phx_3_a1_preg1 = ${q://QID31/ChoiceTextEntryValue/2}

X→

ab_9_a1_per0_preg1 Approximately how many weeks ${word_preg/ChoiceTextEntryValue/2} were you at the time of this most recent ${word_abortion/ChoiceTextEntryValue/2}?

○ Number of weeks: (1) ____

○ I don't know (2)

Display This Question:

If word_preg = No, I use a different word. The word I use instead of "pregnant" is:

And word_abortion = No, I use a different word. The word(s) I use instead of "abortion" is:

And word_period = No, I use a different word. The word I use instead of "period" is:

And Loop any: phx_3_a1_preg1 = ${q://QID31/ChoiceTextEntryValue/2}

X→

ab_9_a1_per1_preg1 Approximately how many weeks ${word_preg/ChoiceTextEntryValue/2} were you at the time of this most recent ${word_abortion/ChoiceTextEntryValue/2}? (measured from the date of your last ${word_period/ChoiceTextEntryValue/2})

○ Number of weeks: (1) ____

○ I don't know (2)

Display This Question:

If word_preg = No, I use a different word. The word I use instead of "pregnant" is:

And word_abortion = Yes, I use the word "abortion".

And word_period = No, I use a different word. The word I use instead of "period" is:

And Loop any: phx_3_a0_preg1 = Abortion

Or If

word_abortion = Prefer not to say

And word_period = No, I use a different word. The word I use instead of "period" is:

And word_preg = No, I use a different word. The word I use instead of "pregnant" is:

And Loop any: phx_3_a0_preg1 = Abortion

X→

ab_9_a0_per1_preg1 Approximately how many weeks ${word_preg/ChoiceTextEntryValue/2} were you at the time of this most recent abortion? (measured from the date of your last ${word_period/ChoiceTextEntryValue/2})

○ Number of weeks: (1) ____

○ I don't know (2)

Display This Question:

If word_abortion = Yes, I use the word "abortion".

And word_period = No, I use a different word. The word I use instead of "period" is:

And word_preg = Yes, I use the word "pregnant".

And Loop any: phx_3_a0_preg0 = Abortion

Or If

word_abortion = Yes, I use the word "abortion".

And word_period = No, I use a different word. The word I use instead of "period" is:

And word_preg = Prefer not to say

And Loop any: phx_3_a0_preg0 = Abortion

Or If

word_abortion = Prefer not to say

And word_period = No, I use a different word. The word I use instead of "period" is:

And word_preg = Yes, I use the word "pregnant".

And Loop any: phx_3_a0_preg0 = Abortion

Or If

word_abortion = Prefer not to say

And word_period = No, I use a different word. The word I use instead of "period" is:

And word_preg = Prefer not to say

And Loop any: phx_3_a0_preg0 = Abortion

ab_9_a0_per1_preg0 Approximately how many weeks pregnant were you at the time of this most recent abortion? (measured from the date of your last ${word_period/ChoiceTextEntryValue/2})

◯ Number of weeks: (1) ____

◯ I don't know (2)

Display This Question:

If word_preg = Yes, I use the word "pregnant".

And word_abortion = No, I use a different word. The word(s) I use instead of "abortion" is:

And word_period = No, I use a different word. The word I use instead of "period" is:

And Loop any: phx_3_a1_preg0 = ${q://QID31/ChoiceTextEntryValue/2}

Or If

word_preg = Prefer not to say

And word_period = No, I use a different word. The word I use instead of "period" is:

And word_abortion = No, I use a different word. The word(s) I use instead of "abortion" is:

And Loop any: phx_3_a1_preg0 = ${q://QID31/ChoiceTextEntryValue/2}

X→

ab_9_a1_per1_preg0 Approximately how many weeks pregnant were you at the time of this most recent ${word_abortion/ChoiceTextEntryValue/2}? (measured from the date of your last ${word_period/ChoiceTextEntryValue/2})

- ○ Number of weeks: (1) ____
- ○ I don't know (2)

Display This Question:

If word_abortion = No, I use a different word. The word(s) I use instead of "abortion" is:

And word_period = Yes, I use the word "period".

And word_preg = Yes, I use the word "pregnant".

And Loop any: phx_3_a1_preg0 = ${q://QID31/ChoiceTextEntryValue/2}

Or If

word_abortion = No, I use a different word. The word(s) I use instead of "abortion" is:

And word_period = Prefer not to say

And word_preg = Yes, I use the word "pregnant".

And Loop any: phx_3_a1_preg0 = ${q://QID31/ChoiceTextEntryValue/2}

Or If

word_abortion = No, I use a different word. The word(s) I use instead of "abortion" is:

And word_period = Yes, I use the word "period".

And word_preg = Prefer not to say

And Loop any: phx_3_a1_preg0 = ${q://QID31/ChoiceTextEntryValue/2}

Or If

word_abortion = No, I use a different word. The word(s) I use instead of "abortion" is:

And word_period = Prefer not to say

And word_preg = Prefer not to say

And Loop any: phx_3_a1_preg0 = ${q://QID31/ChoiceTextEntryValue/2}

X→

ab_9_a1_per0_preg0 Approximately how many weeks pregnant were you at the time of this most recent ${word_abortion/ChoiceTextEntryValue/2}? (measured from the date of your last period)

○ Number of weeks: (1) ____

○ I don't know (2)

Display This Question:

If word_abortion = Yes, I use the word "abortion".

Or word_abortion = Prefer not to say

And If

Loop any: phx_3_a0_preg0 = Abortion

Or Loop any: phx_3_a0_preg1 = Abortion

ab_10_a0 Is there anything you would recommend to improve the abortion care that you received? Select all that apply.

- ☐ Closer clinic/office location (1)
- ☐ Gender-neutral language used by staff (2)
- ☐ Intake forms that are gender-neutral or gender-affirming (3)
- ☐ Intake forms that are affirming of all sexual orientations (4)
- ☐ More privacy outside of the clinic (5)
- ☐ More privacy within the clinic (6)
- ☐ More support from my provider (7)
- ☐ More support from the clinic staff (8)
- ☐ Not listed (please specify): (9) ____
- ☐ ☐ None of these (10)

Display This Question:

If word_abortion = No, I use a different word. The word(s) I use instead of "abortion" is:

And If

Loop any: phx_3_a1_preg1 = ${q://QID31/ChoiceTextEntryValue/2}

Or Loop any: phx_3_a1_preg0 = ${q://QID31/ChoiceTextEntryValue/2}

ab_10_a1 Is there anything you would recommend to improve the ${word_abortion/ChoiceTextEntryValue/2} care that you received? Select all that apply.

- ▢ Closer clinic/office location (1)
- ▢ Gender-neutral language used by staff (2)
- ▢ Intake forms that are gender-neutral or gender-affirming (3)
- ▢ Intake forms that are affirming of all sexual orientations (4)
- ▢ More privacy outside of the clinic (5)
- ▢ More privacy within the clinic (6)
- ▢ More support from my provider (7)
- ▢ More support from the clinic staff (8)
- ▢ Not listed (please specify): (9) ____
- ▢ ⊠None of these (10)

Page Break

Display This Question:

If word_abortion = Yes, I use the word "abortion".

And word_preg = Prefer not to say

And Loop any: phx_3_a0_preg0 = Abortion

Or If

word_abortion = Prefer not to say

And word_preg = Prefer not to say

And Loop any: phx_3_a0_preg0 = Abortion

Or If

word_abortion = Prefer not to say

And word_preg = Yes, I use the word "pregnant".

And Loop all: phx_3_a0_preg0 = Abortion

Or If

word_abortion = Yes, I use the word "abortion".

And word_preg = Yes, I use the word "pregnant".

And Loop any: phx_3_a0_preg0 = Abortion

ab_11_a0_preg0 How much time passed from when you found out you were pregnant to when you **decided** to have an abortion?

- ◯ Less than one day (I knew almost immediately) (1)
- ◯ Several days (2)
- ◯ One week (3)
- ◯ Two weeks (4)
- ◯ Three weeks (5)
- ◯ One month or more (6)
- ◯ I don't remember (7)
- ◯ The abortion was not my decision - someone else decided for me (8)

Display This Question:

If word_abortion = No, I use a different word. The word(s) I use instead of "abortion" is:

And word_preg = Prefer not to say

And Loop any: phx_3_a1_preg0 = ${q://QID31/ChoiceTextEntryValue/2}

Or If

word_abortion = No, I use a different word. The word(s) I use instead of "abortion" is:

And word_preg = Yes, I use the word "pregnant".

And Loop any: phx_3_a1_preg0 = ${q://QID31/ChoiceTextEntryValue/2}

ab_11_a1_preg0 How much time passed from when you found out you were pregnant to when you **decided** to have an ${word_abortion/ChoiceTextEntryValue/2}?

- Less than one day (I knew almost immediately) (1)
- Several days (2)
- One week (3)
- Two weeks (4)
- Three weeks (5)
- One month or more (6)
- I don't remember (7)
- The ${word_abortion/ChoiceTextEntryValue/2} was not my decision - someone else decided for me (8)

Display This Question:

If word_preg = No, I use a different word. The word I use instead of "pregnant" is:

And word_abortion = No, I use a different word. The word(s) I use instead of "abortion" is:

And Loop any: phx_3_a1_preg1 = ${q://QID31/ChoiceTextEntryValue/2}

ab_11_a1_preg1 How much time passed from when you found out you were ${word_preg/ChoiceTextEntryValue/2} to when you **decided** to have an ${word_abortion/ChoiceTextEntryValue/2}?

◯ Less than one day (I knew almost immediately) (1)

◯ Several days (2)

◯ One week (3)

◯ Two weeks (4)

◯ Three weeks (5)

◯ One month or more (6)

◯ I don't remember (7)

◯ The ${word_abortion/ChoiceTextEntryValue/2} was not my decision - someone else decided for me (8)

Display This Question:

If word_abortion = Prefer not to say

And word_preg = No, I use a different word. The word I use instead of "pregnant" is:

And Loop any: phx_3_a0_preg1 = Abortion

Or If

word_abortion = Yes, I use the word "abortion".

And word_preg = No, I use a different word. The word I use instead of "pregnant" is:

And Loop any: phx_3_a0_preg1 = Abortion

ab_11_a0_preg1 How much time passed from when you found out you were ${word_preg/ChoiceTextEntryValue/2} to when you **decided** to have an abortion?

- Less than one day (I knew almost immediately) (1)
- Several days (2)
- One week (3)
- Two weeks (4)
- Three weeks (5)
- One month or more (6)
- I don't remember (7)
- The abortion was not my decision - someone else decided for me (8)

Display This Question:

If Loop any: phx_3_a0_preg0 = Abortion

Or Loop any: phx_3_a0_preg1 = Abortion

And If

word_abortion = Yes, I use the word "abortion".

Or word_abortion = Prefer not to say

ab_12_a0 How much time passed from when you decided to have an abortion to when you **obtained** the abortion?

- Less than one day (1)
- Several days (2)
- One week (3)
- Two weeks (4)
- Three weeks (5)
- One month or more (6)
- I don't remember (7)

Display This Question:

If Loop any: phx_3_a1_preg1 = ${q://QID31/ChoiceTextEntryValue/2}

Or Loop any: phx_3_a1_preg0 = ${q://QID31/ChoiceTextEntryValue/2}

And If

word_abortion = No, I use a different word. The word(s) I use instead of "abortion" is:

ab_12_a1 How much time passed from when you decided to have an ${word_abortion/ChoiceTextEntryValue/2} to when you **obtained** the ${word_abortion/ChoiceTextEntryValue/2}?

- Less than one day (1)
- Several days (2)
- One week (3)
- Two weeks (4)
- Three weeks (5)
- One month or more (6)
- I don't remember (7)

Display This Question:

If Loop any: phx_3_a0_preg0 = Abortion

And If

word_abortion = Yes, I use the word "abortion".

Or word_abortion = Prefer not to say

ab_13_a0_preg0 Would you have preferred to obtain an abortion earlier in your pregnancy?

- Yes (1)
- No (0)
- I don't know (2)

Display This Question:

If Loop any: phx_3_a0_preg1 = Abortion

And If

word_abortion = Yes, I use the word "abortion".

Or word_abortion = Prefer not to say

ab_13_a0_preg1 Would you have preferred to obtain an abortion earlier in your ${word_preg/ChoiceTextEntryValue/2}?

- Yes (1)
- No (0)
- I don't know (2)

Display This Question:

If Loop any: phx_3_a1_preg0 = ${q://QID31/ChoiceTextEntryValue/2}

ab_13_a1_preg0 Would you have preferred to obtain an ${word_abortion/ChoiceTextEntryValue/2} earlier in your pregnancy?

- Yes (1)
- No (0)
- I don't know (2)

Display This Question:

If Loop any: phx_3_a1_preg1 = ${q://QID31/ChoiceTextEntryValue/2}

ab_13_a1_preg1 Would you have preferred to obtain an ${word_abortion/ChoiceTextEntryValue/2} earlier in your ${word_preg/ChoiceTextEntryValue/2}?

- Yes (1)
- No (0)
- I don't know (2)

Page Break

Display This Question:

If word_abortion = Yes, I use the word "abortion".

Or word_abortion = Prefer not to say

And If

Loop any: phx_3_a0_preg0 = Abortion

Or Loop any: phx_3_a0_preg1 = Abortion

ab_14_a0 What factors have made it **EASIER** for you to access abortion care? Select all that apply.

- Affirming and inclusive language used by abortion providers (1)
- Gender-neutral patient materials and intake forms (2)
- Patient materials and intake forms that are affirming of all sexual orientations (3)
- I have a friend(s), partner(s), and/or other people that support me (4)
- My insurance covers abortion (5)
- Sliding scale fees make the cost affordable (6)
- There is an abortion provider near me (7)
- Not listed (please specify): (8) ____
- None of these (9)

Display This Question:

If word_abortion = No, I use a different word. The word(s) I use instead of "abortion" is:

And If

Loop any: phx_3_a1_preg1 = ${q://QID31/ChoiceTextEntryValue/2}

Or Loop any: phx_3_a1_preg0 = ${q://QID31/ChoiceTextEntryValue/2}

ab_14_a1 What factors have made it **EASIER** for you to access ${word_abortion/ChoiceTextEntryValue/2} care? Select all that apply.

- ☐ Affirming and inclusive language used by ${word_abortion/ChoiceTextEntryValue/2} providers (1)
- ☐ Gender-neutral patient materials and intake forms (2)
- ☐ Patient materials and intake forms that are affirming of all sexual orientations (3)
- ☐ I have a friend(s), partner(s), and/or other people that support me (4)
- ☐ My insurance covers ${word_abortion/ChoiceTextEntryValue/2} (5)
- ☐ Sliding scale fees make the cost affordable (6)
- ☐ There is an ${word_abortion/ChoiceTextEntryValue/2} provider near me (7)
- ☐ Not listed (please specify): (8) ____
- ☐ ☐ None of these (9)

Display This Question:

If word_abortion = Yes, I use the word "abortion".

And word_preg = Yes, I use the word "pregnant".

And Loop any: phx_3_a0_preg0 = Abortion

Or If

word_abortion = Yes, I use the word "abortion".

And word_preg = Prefer not to say

And Loop any: phx_3_a0_preg0 = Abortion

Or If

word_abortion = Prefer not to say

And word_preg = Prefer not to say

And Loop any: phx_3_a0_preg0 = Abortion

Or If

word_abortion = Prefer not to say

And word_preg = Yes, I use the word "pregnant".

And Loop any: phx_3_a0_preg0 = Abortion

X→

ab_15_a0_preg0 What factors made it HARDER for you to access abortion care? Select all that apply.

- ☐ I didn't know I was pregnant (1)
- ☐ Difficulty finding an abortion provider (2)
- ☐ I found out/it was confirmed late in the pregnancy that I had a medical issue that affected my ability to have a healthy pregnancy (3)
- ☐ I found out/it was confirmed late in the pregnancy that the fetus has a malformation (4)
- ☐ I had medical concerns regarding the safety of having an abortion (5)
- ☐ I had religious or moral concerns (6)
- ☐ It was a difficult decision for me to make (7)
- ☐ I was worried about the treatment I would receive as a transgender/gender expansive person (8)
- ☐ I was worried about the treatment I would receive as a LGBQ+ person (9)
- ☐ Travel required to get the abortion (10)
- ☐ The cost of the abortion (11)
- ☐ Objections to abortion from family/friends/partner (12)
- ☐ Something changed in my relationship with the person involved in the pregnancy (13)
- ☐ There is a legally required waiting period for abortion (14)
- ☐ Taking time off from work or school (15)
- ☐ Another reason (please specify): (16) ____
- ☐ ☐ None of these (17)

Display This Question:

If word_abortion = No, I use a different word. The word(s) I use instead of "abortion" is:

And word_preg = Yes, I use the word "pregnant".

And Loop any: phx_3_a1_preg0 = ${q://QID31/ChoiceTextEntryValue/2}

Or If

word_preg = Prefer not to say

And word_abortion = No, I use a different word. The word(s) I use instead of "abortion" is:

And Loop any: phx_3_a1_preg0 = ${q://QID31/ChoiceTextEntryValue/2}

ab_15_a1_preg0 What factors made it **HARDER** for you to access ${word_abortion/ChoiceTextEntryValue/2} care? Select all that apply.

- ☐ I didn't know I was pregnant (1)
- ☐ Difficulty finding an ${word_abortion/ChoiceTextEntryValue/2} provider (2)
- ☐ I found out/it was confirmed late in the pregnancy that I had a medical issue that affected my ability to have a healthy pregnancy (3)
- ☐ I found out/it was confirmed late in the pregnancy that the fetus has a malformation (4)
- ☐ I had medical concerns regarding the safety of having an ${word_abortion/ChoiceTextEntryValue/2} (5)
- ☐ I had religious or moral concerns (6)
- ☐ It was a difficult decision for me to make (7)
- ☐ I was worried about the treatment I would receive as a transgender/gender expansive person (8)
- ☐ I was worried about the treatment I would receive as a LGBQ+ person (9)
- ☐ Travel required to get the ${word_abortion/ChoiceTextEntryValue/2} (10)
- ☐ The cost of the ${word_abortion/ChoiceTextEntryValue/2} (11)
- ☐ Objections to abortion from family/friends/partner (12)
- ☐ Something changed in my relationship with the person involved in the pregnancy (13)
- ☐ There is a legally required waiting period for ${word_abortion/ChoiceTextEntryValue/2} (14)
- ☐ Taking time off for work or school (15)
- ☐ Another reason (please specify): (16) ____
- ☐ ☐ None of these (17)

Display This Question:

If word_abortion = Yes, I use the word "abortion".

And word_preg = No, I use a different word. The word I use instead of "pregnant" is:

And Loop any: phx_3_a0_preg1 = Abortion

Or If

word_preg = No, I use a different word. The word I use instead of "pregnant" is:

And word_abortion = Prefer not to say

And Loop any: phx_3_a0_preg1 = Abortion

ab_15_a0_preg1 What factors made it **HARDER** for you to access abortion care? Select all that apply.

- [ ] I didn't know I was ${word_preg/ChoiceTextEntryValue/2} (1)
- [ ] Difficulty finding an abortion provider (2)
- [ ] I found out/it was confirmed late in the ${word_preg/ChoiceTextEntryValue/2} that I had a medical issue that affected my ability to have a healthy ${word_preg/ChoiceTextEntryValue/2} (3)
- [ ] I found out/it was confirmed late in the ${word_preg/ChoiceTextEntryValue/2} that the fetus has a malformation (4)
- [ ] I had medical concerns regarding the safety of having an abortion (5)
- [ ] I had religious or moral concerns (6)
- [ ] It was a difficult decision for me to make (7)
- [ ] I was worried about the treatment I would receive as a transgender/gender expansive person (8)
- [ ] I was worried about the treatment I would receive as a LGBQ+ person (9)
- [ ] Travel required to get the abortion (10)
- [ ] The cost of the abortion (11)
- [ ] Objections to abortion from family/friends/partner (12)
- [ ] Something changed in my relationship with the person involved in the ${word_preg/ChoiceTextEntryValue/2} (13)
- [ ] There is a legally required waiting period for abortion (14)
- [ ] Taking time off from work or school (15)
- [ ] Another reason (please specify): (16) ____
- [ ] None of these (17)

Display This Question:

If word_abortion = No, I use a different word. The word(s) I use instead of "abortion" is:

And word_preg = No, I use a different word. The word I use instead of "pregnant" is:

And Loop any: phx_3_a1_preg1 = ${q://QID31/ChoiceTextEntryValue/2}

ab_15_a1_preg1 What factors made it **HARDER** for you to access ${word_abortion/ChoiceTextEntryValue/2} care? Select all that apply.

- ☐ I didn't know I was ${word_preg/ChoiceTextEntryValue/2} (1)
- ☐ Difficulty finding an ${word_abortion/ChoiceTextEntryValue/2} provider (2)
- ☐ I found out/it was confirmed late in the ${word_preg/ChoiceTextEntryValue/2} that I had a medical issue that affected my ability to have a healthy ${word_preg/ChoiceTextEntryValue/2} (3)
- ☐ I found out/it was confirmed late in the ${word_preg/ChoiceTextEntryValue/2} that the fetus has a malformation (4)
- ☐ I had medical concerns regarding the safety of having an ${word_abortion/ChoiceTextEntryValue/2} (5)
- ☐ I had religious or moral concerns (6)
- ☐ It was a difficult decision for me to make (7)
- ☐ I was worried about the treatment I would receive as a transgender/gender expansive person (8)
- ☐ I was worried about the treatment I would receive as a LGBQ+ person (9)
- ☐ Travel required to get the ${word_abortion/ChoiceTextEntryValue/2} (10)
- ☐ The cost of the ${word_abortion/ChoiceTextEntryValue/2} (11)
- ☐ Objections to ${word_abortion/ChoiceTextEntryValue/2} from family/friends/partner (12)
- ☐ Something changed in my relationship with the person involved in the ${word_preg/ChoiceTextEntryValue/2} (13)
- ☐ There is a legally required waiting period for ${word_abortion/ChoiceTextEntryValue/2} (14)
- ☐ Taking time off from work or school (15)
- ☐ Another reason (please specify): (16)

____

None of these (17)

Display This Question:

If word_abortion = Yes, I use the word "abortion".

Or word_abortion = Prefer not to say

And If

legal = My health insurance

And If

If How many times have you been pregnant? Text Response Is Greater Than or Equal to 1

Or How many times have you been ${q://QID28/ChoiceTextEntryValue/2}? Text Response Is Greater Than or Equal to 1

ab_16_a0 Has your legal gender on your health insurance prevented you from having an abortion covered by your insurance?

- Yes (1)
- No (0)
- I have not tried to use my insurance to cover this service (3)
- My insurance does not cover this service (4)
- I don't know (2)

Display This Question:

If word_abortion = No, I use a different word. The word(s) I use instead of "abortion" is:

And legal = My health insurance

And If

If How many times have you been pregnant? Text Response Is Greater Than or Equal to 1

Or How many times have you been ${q://QID28/ChoiceTextEntryValue/2}? Text Response Is Greater Than or Equal to 1

ab_16_a1 Has your legal gender on your health insurance prevented you from having an ${word_abortion/ChoiceTextEntryValue/2} covered by your insurance?

- Yes (1)
- No (0)
- I have not tried to use my insurance to cover this service (3)
- My insurance does not cover this service (4)
- I don't know (2)

Page Break

Display This Question:

If word_abortion = Yes, I use the word "abortion".

And word_preg = Prefer not to say

And How many times have you been pregnant? Text Response Is Greater Than or Equal to 1

Or If

word_abortion = Prefer not to say

And word_preg = Prefer not to say

And How many times have you been pregnant? Text Response Is Greater Than or Equal to 1

Or If

word_abortion = Prefer not to say

And word_preg = Yes, I use the word "pregnant".

And How many times have you been pregnant? Text Response Is Greater Than or Equal to 1

Or If

word_abortion = Yes, I use the word "abortion".

And word_preg = Yes, I use the word "pregnant".

And How many times have you been pregnant? Text Response Is Greater Than or Equal to 1

sma_1_a0_preg0 People make different choices about how to end an unwanted pregnancy. Some people may go to a hospital, clinic, or doctor's office to have an abortion, while other people may get information from the internet, a friend, or a family member about medicines or herbs they can take on their own, or they may do something else to try to end a pregnancy on their own.

Have you ever **CONSIDERED** trying to end a pregnancy on your own, without medical supervision?

- Yes (1)
- No (0)

Display This Question:

If word_preg = Prefer not to say

And word_abortion = No, I use a different word. The word(s) I use instead of "abortion" is:

And How many times have you been pregnant? Text Response Is Greater Than or Equal to 1

Or If

word_preg = Yes, I use the word "pregnant".

And word_abortion = No, I use a different word. The word(s) I use instead of "abortion" is:

And How many times have you been pregnant? Text Response Is Greater Than or Equal to 1

sma_1_a1_preg0 People make different choices about how to end an unwanted pregnancy. Some people may go to a hospital, clinic, or doctor's office to have an ${word_abortion/ChoiceTextEntryValue/2}, while other people may get information from the internet, a friend, or a family member about medicines or herbs they can take on their own, or they may do something else to try to end a pregnancy on their own.

Have you ever **CONSIDERED** trying to end a pregnancy on your own, without medical supervision?

- Yes (1)
- No (0)

Display This Question:

If word_abortion = No, I use a different word. The word(s) I use instead of "abortion" is:

And word_preg = No, I use a different word. The word I use instead of "pregnant" is:

And If

If How many times have you been ${q://QID28/ChoiceTextEntryValue/2}? Text Response Is Greater Than or Equal to 1

sma_1_a1_preg1 People make different choices about how to end an unwanted ${word_preg/ChoiceTextEntryValue/2}. Some people may go to a hospital, clinic, or doctor's office to have an ${word_abortion/ChoiceTextEntryValue/2}, while other people may get information from the internet, a friend, or a family member about medicines or herbs they can take on their own, or they may do something else to try to end a ${word_preg/ChoiceTextEntryValue/2} on their own.

Have you ever **CONSIDERED** trying to end a ${word_preg/ChoiceTextEntryValue/2} on your own, without medical supervision?

- Yes (1)
- No (0)

Display This Question:

If word_preg = No, I use a different word. The word I use instead of "pregnant" is:

And word_abortion = Prefer not to say

And How many times have you been ${q://QID28/ChoiceTextEntryValue/2}? Text Response Is Greater Than or Equal to 1

Or If

word_preg = No, I use a different word. The word I use instead of "pregnant" is:

And word_abortion = Yes, I use the word "abortion".

And How many times have you been ${q://QID28/ChoiceTextEntryValue/2}? Text Response Is Greater Than or Equal to 1

sma_1_a0_preg1 People make different choices about how to end an unwanted ${word_preg/ChoiceTextEntryValue/2}. Some people may go to a hospital, clinic, or doctor's office to have an abortion, while other people may get information from the internet, a friend, or a family member about medicines or herbs they can take on their own, or they may do something else to try to end a ${word_preg/ChoiceTextEntryValue/2} on their own.

Have you ever **CONSIDERED** trying to end a ${word_preg/ChoiceTextEntryValue/2} on your own, without medical supervision?

- Yes (1)
- No (0)

Display This Question:

If sma_1_a0_preg0 = Yes

Or sma_1_a1_preg0 = Yes

sma_2_preg0 Have you ever **ATTEMPTED** to end a pregnancy on your own, without medical supervision?

◯ Yes (1)

◯ No (0)

Display This Question:

If sma_1_a1_preg1 = Yes

Or sma_1_a0_preg1 = Yes

sma_2_preg1 Have you ever **ATTEMPTED** to end a ${word_preg/ChoiceTextEntryValue/2} on your own, without medical supervision?

◯ Yes (1)

◯ No (0)

Display This Question:

If sma_2_preg0 = Yes

sma_3_preg0 Please tell us in your own words about how you attempted to end your pregnancy without medical supervision.

____

____

____

____

____

Display This Question:

If sma_2_preg1 = Yes

sma_3_preg1 Please tell us in your own words about how you attempted to end your ${word_preg/ChoiceTextEntryValue/2} without medical supervision.

____

____

____

____

____

End of Block: Sexual and reproductive health care

Start of Block: Quality of health care

qoc_0 In this section, we will ask you a set of questions about your experiences with health care providers, particularly related to sexual and/or reproductive healthcare.

qoc_1 For people in your life who do not know you, what sexual orientation do they USUALLY think you are? **(Choose one.)**

- Asexual (1)
- Bisexual (2)
- Gay (3)
- Heterosexual or Straight (4)
- Lesbian (5)
- Queer (6)
- Another sexual orientation (7)
- They cannot tell (8)
- It varies (9)
- I don't know what they think (10)

qoc_2 What percent of your HEALTH CARE PROVIDERS do you think are aware of your sexual orientation (meaning they are aware of whether you consider yourself straight, gay, etc.)?

- 0% (1)
- 10% (2)
- 20% (3)
- 30% (4)
- 40% (5)
- 50% (6)
- 60% (7)
- 70% (8)
- 80% (9)
- 90% (10)
- 100% (11)
- I don't know (12)

qoc_3 For people in your life who do not know you, what gender do they USUALLY think you are? **(Choose one.)**

- ◯ Cisgender Man (1)
- ◯ Cisgender Woman (2)
- ◯ Transgender Man (3)
- ◯ Transgender Woman (4)
- ◯ Man (10)
- ◯ Woman (11)
- ◯ Non-binary/Genderqueer (5)
- ◯ They cannot tell (6)
- ◯ It varies (7)
- ◯ I don't know what they think (8)
- ◯ Another gender (9) ____

qoc_4 What percent of your HEALTH CARE PROVIDERS do you think are aware of your gender identity?

- 0% (1)
- 10% (2)
- 20% (3)
- 30% (4)
- 40% (5)
- 50% (6)
- 60% (7)
- 70% (8)
- 80% (9)
- 90% (10)
- 100% (11)
- I don't know (12)

qoc_5 In a health care setting, have you ever felt that opinions about your gender identity and/or sexual orientation from health care staff have negatively impacted you (whether these opinions were said out loud or suggested with body language)?

- ◯ Yes - my gender identity (1)
- ◯ Yes - my sexual orientation (2)
- ◯ Yes - my gender identity AND my sexual orientation (3)
- ◯ ☐ No (0)
- ◯ ☐ I don't know (4)

Display This Question:

If ga_1 = Gender affirming genital surger(ies) / reconstructive surger(ies) or bottom surger(ies)

Or ga_1 = Hormone Treatment / Hormone Replacement Therapy (HRT)

Or ga_1 = Medications to stop or delay the onset of puberty (often called "puberty blockers", usually used by youth)

And If

qoc_5 = Yes - my gender identity

X→

qoc_6 When did these negative interactions with health care staff take place in relation to your hormonal and/or surgical gender-affirmation process?

- ◯ Before I began my hormonal and/or surgical gender affirmation process (1)
- ◯ After I began my hormonal and/or surgical gender affirmation process (3)

X→

qoc_7 In the past year, did any of these things happen to you when you went to see a doctor or health care provider?

A warning that some of these experiences can be upsetting, and may trigger difficult memories/emotions.

Select all that apply.

- ☐ A doctor/health care provider refused to give me health care (1)
- ☐ A doctor/health care provider refused to give me trans-related care (2)
- ☐ A doctor/health care provider refused to give me LGBQ+ related care (18)
- ☐ A doctor/health care provider used harsh or abusive language when treating me (3)
- ☐ A doctor/health care provider was physically rough or abusive when treating me (4)
- ☐ I experienced unwanted sexual contact (such as fondling, sexual assault, or rape) in a health care setting (5)
- ☐ I had to teach my doctor/health care provider about LGBQ+ people so that I could get appropriate care (15)
- ☐ I had to teach my doctor/health care provider about transgender and gender expansive people so that I could get appropriate care (6)
- ☐ I received varied levels of care from different providers and staff within the same visit (7)
- ☐ I was physically attacked by someone during my visit in a health care setting (8)
- ☐ I was referred to specialists, labs, pharmacies, etc. who/that were not gender-affirming or inclusive (9)
- ☐ I was referred to specialists, labs, pharmacies, etc. who/that were not LGBQ+ affirming or inclusive (19)
- ☐ I was verbally harassed in a health care setting (such as a hospital, office, clinic, etc.) (10)
- ☐ My doctor/health care provider asked me unnecessary/invasive questions about my gender status that were not related to the reason for my visit (11)

My doctor/healthcare provider asked me unnecessary/invasive questions about my sexual orientation that were not related to the reason for my visit (16)

My doctor/health care provider knew I was transgender or gender expansive and treated me with respect (12)

My doctor/health care provider knew I was LGBQ+ and treated me with respect (20)

Not listed (please describe): (13)
____

None of the above (14)

End of Block: Quality of health care

Start of Block: Sociodemographic characteristics

sd_0 Finally, in this last section of the survey, we would like to ask you a few questions about yourself so we can better understand how experiences vary by individual characteristics.

sd_1 Which category(ies) best describe you? Select all that apply.

- ▢ American Indian or Alaska Native - What tribe(s) are you affiliated with? (1) ____
- ▢ Black or African American (2)
- ▢ Central Asian (3)
- ▢ East Asian (4)
- ▢ Hispanic or Latinx (5)
- ▢ Middle Eastern or North African (6)
- ▢ Native Hawaiian and Pacific Islander (7)
- ▢ South Asian (8)
- ▢ South East Asian (9)
- ▢ White (10)
- ▢ Unknown (11)
- ▢ Not listed, please tell us: (12) ____
- ▢ ▢ None of these (13)

sd_2 What is your current relationship status? Select all that apply.

- ☐ Not in a relationship (1)
- ☐ In a relationship with one person, not living with partner (2)
- ☐ In a relationship with one person, living with partner (3)
- ☐ In a relationship with more than one person, not living with partner(s) (4)
- ☐ In a relationship with more than one person, living with partner(s) (5)
- ☐ Not listed (please describe): (6) ____

---

sd_3 What is your current legal marital status?

- ○ Divorced (1)
- ○ Legally recognized civil union (2)
- ○ Married (3)
- ○ Registered domestic partnership (4)
- ○ Separated (5)
- ○ Single, never married (6)
- ○ Widowed (7)
- ○ Not listed (please specify): (8) ____
- ○ None of these (9)

---

sd_4 Are you a parent? This includes parenting children who are now adults, are deceased, or are not biologically related to you.

- Yes (1)
- No (0)

Display This Question:

If sd_4 = Yes

sd_5 To how many people are you/have you been a parent? This includes children who are now adults, are deceased, or are not biologically related to you.

▼ 1 (1) ... 10 or more children (10)

Display This Question:

If word_sperm = No, I use a different word. The word I use instead of "sperm" is:

And word_preg = No, I use a different word. The word I use instead of "pregnant" is:

And sd_4 = Yes

sd_6_preg1_s1 Please indicate how you became a parent. **Select all that apply.**

- The egg was fertilized through sexual activity with another parent of the child (1)
- I carried the child through a ${word_preg/ChoiceTextEntryValue/2} and was also the egg source for the child (2)
- I carried the child through a ${word_preg/ChoiceTextEntryValue/2} but was NOT the egg source for the child (3)
- I provided the egg for the child that another person carried through the ${word_preg/ChoiceTextEntryValue/2} (4)
- I provided the ${word_sperm/ChoiceTextEntryValue/2} for the child (5)
- I adopted the child (6)
- I used donor (anonymous) ${word_sperm/ChoiceTextEntryValue/2} for the child (7)
- I used donor (known) ${word_sperm/ChoiceTextEntryValue/2} for the child (8)
- I underwent a second parent adoption of my partner's biological child (9)
- I worked with a surrogate to carry the child (10)
- I worked with an egg donor to provide the egg source for the child (11)
- I am a step parent to the child (12)
- I am a foster parent to the child (13)
- I became a parent through another method (please specify): (14) ____
- None of these (15)

Display This Question:

If word_sperm = No, I use a different word. The word I use instead of "sperm" is:

And word_preg = Yes, I use the word "pregnant".

And sd_4 = Yes

Or If

word_sperm = No, I use a different word. The word I use instead of "sperm" is:

And word_preg = Prefer not to say

And sd_4 = Yes

X→

sd_6_preg0_s1 Please indicate how you became a parent. **Select all that apply.**

- ☐ The egg was fertilized through sexual activity with another parent of the child (1)
- ☐ I carried the child through a pregnancy and was also the egg source for the child (2)
- ☐ I carried the child through a pregnancy but was NOT the egg source for the child (3)
- ☐ I provided the egg for the child that another person carried through the pregnancy (4)
- ☐ I provided the ${word_sperm/ChoiceTextEntryValue/2} for the child (5)
- ☐ I adopted the child (6)
- ☐ I used donor (anonymous) ${word_sperm/ChoiceTextEntryValue/2} for the child (7)
- ☐ I used donor (known) ${word_sperm/ChoiceTextEntryValue/2} for the child (8)
- ☐ I underwent a second parent adoption of my partner's biological child (9)
- ☐ I worked with a surrogate to carry the child (10)
- ☐ I worked with an egg donor to provide the egg source for the child (11)
- ☐ I am a step parent to the child (12)
- ☐ I am a foster parent to the child (13)
- ☐ I became a parent through another method (please specify): (14) ____
- ☐ ☐ None of these (15)

Display This Question:

If word_sperm = Prefer not to say

And word_preg = No, I use a different word. The word I use instead of "pregnant" is:

And sd_4 = Yes

Or If

word_sperm = Yes, I use the word "sperm".

And word_preg = No, I use a different word. The word I use instead of "pregnant" is:

And sd_4 = Yes

sd_6_preg1_s0 Please indicate how you became a parent. **Select all that apply.**

- ☐ The egg was fertilized through sexual activity with another parent of the child (1)
- ☐ I carried the child through a ${word_preg/ChoiceTextEntryValue/2} and was also the egg source for the child (2)
- ☐ I carried the child through a ${word_preg/ChoiceTextEntryValue/2} but was NOT the egg source for the child (3)
- ☐ I provided the egg for the child that another person carried through the ${word_preg/ChoiceTextEntryValue/2} (4)
- ☐ I provided the sperm for the child (5)
- ☐ I adopted the child (6)
- ☐ I used donor (anonymous) sperm for the child (7)
- ☐ I used donor (known) sperm for the child (8)
- ☐ I underwent a second parent adoption of my partner's biological child (9)
- ☐ I worked with a surrogate to carry the child (10)
- ☐ I worked with an egg donor to provide the egg source for the child (11)
- ☐ I am a step parent to the child (12)
- ☐ I am a foster parent to the child (13)
- ☐ I became a parent through another method (please specify): (14) ____
- ☐ ☐ None of these (15)

Display This Question:

If word_sperm = Yes, I use the word "sperm".

And word_preg = Prefer not to say

And sd_4 = Yes

Or If

word_sperm = Prefer not to say

And word_preg = Yes, I use the word "pregnant".

And sd_4 = Yes

Or If

word_sperm = Prefer not to say

And word_preg = Prefer not to say

And sd_4 = Yes

Or If

word_sperm = Yes, I use the word "sperm".

And word_preg = Yes, I use the word "pregnant".

And sd_4 = Yes

X→

sd_6_preg0_s0 Please indicate how you became a parent. **Select all that apply.**

- [ ] The egg was fertilized through sexual activity with another parent of the child (1)
- [ ] I carried the child through a pregnancy and was also the egg source for the child (2)
- [ ] I carried the child through a pregnancy but was NOT the egg source for the child (3)
- [ ] I provided the egg for the child that another person carried through the pregnancy (4)
- [ ] I provided the sperm for the child (5)
- [ ] I adopted the child (6)
- [ ] I used donor (anonymous) sperm for the child (7)
- [ ] I used donor (known) sperm for the child (8)
- [ ] I underwent a second parent adoption of my partner's biological child (9)
- [ ] I worked with a surrogate to carry the child (10)
- [ ] I worked with an egg donor to provide the egg source for the child (11)
- [ ] I am a step parent to the child (12)
- [ ] I am a foster parent to the child (13)
- [ ] I became a parent through another method (please specify): (14) ____
- [ ] None of these (15)

sd_7 What are your current living arrangements? **Select all that apply.**

- ☐ Living in a foster group home or other foster care (1)
- ☐ Living in a hospital (2)
- ☐ Living in a hotel or motel that you pay for (3)
- ☐ Living in a house, apartment, or condo that you rent (alone or with others) (4)
- ☐ Living in a house, apartment, or condo that you own (alone or with others) (5)
- ☐ Living in a nursing home or other adult care facility (6)
- ☐ Living in a shelter (including homeless, domestic violence, or other type of emergency shelter) or in a hotel or motel with an emergency shelter voucher (7)
- ☐ Living in campus or university housing (8)
- ☐ Living in military barracks (9)
- ☐ Living in transitional housing or a halfway house (10)
- ☐ Living on the street, in a car, in an abandoned building, in a park, or a place that is NOT a house, apartment, shelter, or other housing (11)
- ☐ Living temporarily with friends or family because you cannot afford your own housing (12)
- ☐ Living with parents or family you grew up with because you have not yet left home (13)
- ☐ Living with a partner, spouse, or other person who pays for the housing (14)
- ☐ Psychiatric hospital or other psychiatric facility (15)
- ☐ Not listed (please describe): (16) ____
- ☐ None of these (17)

sd_8 What is your zip code? (This is the 5-digit code that helps direct U.S. mail to you.)

____

sd_9 What is the highest level of school that you have completed?

- ◯ Some high school or less (1)
- ◯ High school degree or GED (2)
- ◯ Trade or technical school and no degree (3)
- ◯ Trade or technical school degree (4)
- ◯ Some college and no degree (5)
- ◯ College degree (6)
- ◯ Graduate or professional study and no graduate degree (7)
- ◯ Graduate or professional degree (8)

sd_10 How would you describe your work status right now?

- Employed full time (40 hours or more per week) and not a student (1)
- Employed part-time (Up to 39 hours per week) and not a student (2)
- Employed full-time (40 hours or more per week) and in school (3)
- Employed part-time (Up to 39 hours per week) and in school (4)
- Full-time or part-time student, not employed (5)
- Self-employed (6)
- Unemployed (7)
- Retired (8)
- Unable to work (9)
- Not listed (please specify): (10) ____
- None of these (11)

sd_11 What is your approximate ANNUAL **household** income? (This would include the total of any income you make, and that of others in your household with whom you share money)

- Specify amount in US dollars: (1) ____
- Unsure (2)

sd_12 How many individuals are supported by this household income (including yourself)?

▼ 1 (1) ... 15 (15)

sd_13 What are your current sources of income? Select all that apply.

- ☐ Cash assistance from welfare (such as TANF) or other public cash assistance program (DO NOT include food stamps (SNAP) or WIC) (1)
- ☐ Child support or alimony (2)
- ☐ Income from dividends, estates or trusts, royalties, or rental income (3)
- ☐ Interest income (on savings or bonds) (4)
- ☐ Pay from sex work, selling drugs, or other work that is currently considered illegal (5)
- ☐ Pay from your full-time or part-time job (6)
- ☐ Pay from your partner's/spouse's full-time or part-time job (7)
- ☐ Private pension or government employee pension (8)
- ☐ Regular contributions from people who don't live in the household (9)
- ☐ Self-employment income from your own business, profession or trade, or farm (not including underground economy) (10)
- ☐ Social security disability benefits (SSDI) (11)
- ☐ Social security retirement or railroad retirement income (12)
- ☐ Student loans (13)
- ☐ Unemployment benefits (14)
- ☐ Other retirement income (15)
- ☐ Supplemental security income (SSI) (16)
- ☐ Veteran's disability benefits and other Veteran's benefits (17)
- ☐ Workers' comp or other disability (18)

Income not listed above (please specify): (19)

____

None of these (20)

---

sd_14 Do you currently receive assistance from food stamps (SNAP) or the Special Supplemental Nutrition Program for Women, Infants, and Children (WIC)? Select all that apply.

No (0)

Yes, assistance from food stamps (SNAP) (1)

Yes, assistance from WIC (2)

---

sd_15 Are you currently covered by any health insurance or health coverage plan?

No (0)

Yes (1)

I don't know (2)

Skip To: sd_17 If sd_15 = No

Skip To: sd_17 If sd_15 = I don't know

---

sd_16 What type of health insurance or health coverage plan do you have? Select all that apply.

- Indian Health Service (1)
- Insurance through my current or former employer or union (2)
- Insurance through my school or university (3)
- Insurance through someone else's current or former employer or union (4)
- Insurance I or someone else purchased through HealthCare.Gov or a Health Insurance Marketplace (sometimes called "Obamacare") (5)
- Insurance I or someone else purchased directly from an insurance company (6)
- Medicare (for people 65 and older, or people with certain disabilities) (7)
- Medicaid (government assistance plan for those with low incomes or a disability, sometimes referred to by another name depending on your state, for instance "MediCal" in California, "TennCare" in Tennessee, or "MassHealth" in Massachusetts.) (8)
- TRICARE or other military health care VA (including those who have ever used or enrolled for VA health care) (9)
- Any other type of health insurance or health coverage plan (please specify): (10) ____
- None of these (11)

sd_17 Are you a current or former member of the United States Armed Forces?

- Yes (1)
- No (0)

end_qual Is there anything else about your sexual/reproductive health or health care that you would like to tell us?

____

____

____

____

____

End of Block: Sociodemographic characteristics

Start of Block: Email Block

email Thank you so much for taking the time to complete this survey!

If you know of people that may be interested in participating in this survey, please feel free to share this link widely: [INSERT LINK}

This survey is anonymous; no individually-identifying information is connected with your responses. You may elect to enter a lottery for prizes ($50 Amazon gift cards), to sign up to receive a summary about the study results, and/or participate in a follow-up study. You can also receive a copy of the informed consent document and suggest areas or questions for us to investigate. Clicking "Yes" below will result in your leaving the survey and going to a separate webpage, away from your survey submission (with no connection between pages), where you may submit your email address. Clicking "No" will end the survey; you will not have a chance to enter the lottery for prizes or receive additional information.

Would you like to share your email with us, separate from your survey submission?

- ○ Yes, I want to share my email with the study team (1)
- ○ No, I do NOT want to share my email (2)

End of Block: Email Block
