# Supplementary material for: Development of an affirming and customizable electronic survey of sexual and reproductive health experiences for transgender and gender nonbinary people
Source: PLoS One. 2020 May 4;15(5):e0232154. doi: 10.1371/journal.pone.0232154 (PMC7197812; doi:10.1371/journal.pone.0232154)
Supplement: S4 File — (DOCX) [file pone.0232154.s004.docx]

**S3 File. Sample Stata code for collapsing questions corresponding to combinations of customizable words for candidate medical terms.**

**************************************************************************************

*** Collapse responses w/ medical vs customizable words for “abortion” (a0 or a1), “birth control” (bc0 or bc1) and “pregnancy” (preg0 or preg 1): Sample code

**************************************************************************************

/// A simple example: the below question asked respondents “How many times have you been pregnant?” There were two versions of this question – one for people who said that they used the medical term “pregnant”, and another for those who said they used their own write-in word. The below code collapses responses from these two questions into a single new variable (preg_1_new), and labels it as “Number of pregnancies”.

gen preg_1_new=.

foreach var of varlist preg_1_* {

replace preg_1_new=`var' if preg_1_new==.

}

label var preg_1_new "Number of pregnancies"

/// A second, slightly more complex example: This particular question asked participants “From the below list of reproductive health issues facing people who share your sexual orientation and/or gender identity, please select the three that feel most important to you.” There were 17 answer choices, each containing a different combination of three candidate medical terms (abortion, birth control, and pregnancy). The below loop creates 17 new variables, and then pools responses from multiple copies of each answer choice resulting from customizable word combinations into a single variable for each of the 17 answer choices.

foreach x in 1 2 3 4 5 6 7 8 9 10 11 12 13 14 15 16 17 {

gen srh_1_new_`x'=.

replace srh_1_new_`x'=srh_1_a0_bc0_preg0_`x' if srh_1_new_`x'==.

replace srh_1_new_`x'=srh_1_a0_bc1_preg0_`x' if srh_1_new_`x'==.

replace srh_1_new_`x'=srh_1_a0_bc1_preg1_`x' if srh_1_new_`x'==.

replace srh_1_new_`x'=srh_1_a1_bc1_preg1_`x' if srh_1_new_`x'==.

replace srh_1_new_`x'=srh_1_a1_bc1_preg0_`x' if srh_1_new_`x'==.

replace srh_1_new_`x'=srh_1_a1_bc0_preg0_`x' if srh_1_new_`x'==.

replace srh_1_new_`x'=srh_1_a1_bc0_preg1_`x' if srh_1_new_`x'==.

replace srh_1_new_`x'=srh_1_a0_bc0_preg1_`x' if srh_1_new_`x'==.

}

/// The above steps were repeated for all questions in the survey that allowed the substitution of customizable words in place of candidate medical terms.
